# Probiotics Modulate Mouse Gut Microbiota and Influence Intestinal Immune and Serotonergic Gene Expression in a Site-Specific Fashion

**DOI:** 10.3389/fmicb.2021.706135

**Published:** 2021-09-01

**Authors:** Valentina Taverniti, Valentina Cesari, Giorgio Gargari, Umberto Rossi, Cristina Biddau, Cristina Lecchi, Walter Fiore, Stefania Arioli, Ivan Toschi, Simone Guglielmetti

**Affiliations:** ^1^Department of Food, Environmental and Nutritional Sciences, Università degli Studi di Milano, Milan, Italy; ^2^Department of Agricultural and Environmental Sciences, Università degli Studi di Milano, Milan, Italy; ^3^Department of Veterinary Medicine, Università degli Studi di Milano, Milan, Italy; ^4^Sofar S.p.A., Trezzano Rosa, Italy

**Keywords:** S24-7, *in vivo*, SERT, tryptophan hydroxylase, IL-10, zonulin, bifidobacteria

## Abstract

Probiotic microorganisms may benefit the host by influencing diverse physiological processes, whose nature and underlying mechanisms are still largely unexplored. Animal models are a unique tool to understand the complexity of the interactions between probiotic microorganisms, the intestinal microbiota, and the host. In this regard, in this pilot study, we compared the effects of 5-day administration of three different probiotic bacterial strains (*Bifidobacterium bifidum* MIMBb23sg, *Lactobacillus helveticus* MIMLh5, and *Lacticaseibacillus paracasei* DG) on three distinct murine intestinal sites (ileum, cecum, and colon). All probiotics preferentially colonized the cecum and colon. In addition, probiotics reduced in the ileum and increased in the cecum and colon the relative abundance of numerous bacterial taxonomic units. MIMBb23sg and DG increased the inducible nitric oxide synthase (iNOS) in the ileum, which is involved in epithelial homeostasis. In addition, MIMBb23sg upregulated cytokine IL-10 in the ileum and downregulated the cyclooxygenase COX-2 in the colon, suggesting an anti-inflammatory/regulatory activity. MIMBb23sg significantly affected the expression of the main gene involved in serotonin synthesis (TPH1) and the gene coding for the serotonin reuptake protein (SERT) in the ileum and colon, suggesting a potential propulsive effect toward the distal part of the gut, whereas the impact of MIMLh5 and DG on serotonergic genes suggested an effect toward motility control. The three probiotics decreased the expression of the permeability marker zonulin in gut distal sites. This preliminary *in vivo* study demonstrated the safety of the tested probiotic strains and their common ability to modulate the intestinal microbiota. The probiotics affected host gene expression in a strain-specific manner. Notably, the observed effects in the gut were site dependent. This study provides a rationale for investigating the effects of probiotics on the serotonergic system, which is a topic still widely unexplored.

## Introduction

Probiotics are “live microorganisms that, when administered in adequate amounts, confer a health benefit on the host” (Hill et al., 2014). Their consumption has gained wide popularity worldwide, as demonstrated by the constantly increasing number of foods and supplements on the market containing billions of viable microbial cells. Most commercial probiotic products include cells ascribed to three taxonomic groups: bifidobacteria (e.g., *Bifidobacterium animalis* subsp. *lactis*, *B. bifidum*, and *B. longum*), lactobacilli *sensum strictum* (e.g., *Lactobacillus acidophilus* and *Lactobacillus helveticus*), and the *L. casei* group of species (i.e., *Lacticaseibacillus casei*, *Lacticaseibacillus paracasei*, and *Lacticaseibacillus rhamnosus*). Numerous human trials have demonstrated the ability of these bacteria to promote health by preventing infections, improving gastro-intestinal conditions, or ameliorating immune disorders (Wilkins and Sequoia, 2017; Wang et al., 2019). Possible mechanisms have been proposed for the ability of probiotics to benefit host health, including the preservation of intestinal integrity (Khailova et al., 2010), the modification of intestinal butyrate levels through the modulation of the gut microbial taxonomic structure (Ferrario et al., 2014; Gargari et al., 2016), and the stimulation of the immune system mediated by microbe-associated molecular patterns (MAMPs) (Taverniti et al., 2013; Guglielmetti et al., 2014; Balzaretti et al., 2017). Nonetheless, a comprehensive understanding of probiotics’ host-interaction properties is yet to be obtained.

The main target of the probiotic action is the intestinal tract, a complex environment whose functioning depends on the contribution of the resident microbiota, the mucosa-associated lymphoid tissue (MALT), and the neuro-endocrine system. These factors, which can all be affected by probiotics, vary along the intestinal tract, and variably interact with each other to determine gut homeostasis (Farzi et al., 2018). Consequently, the mechanisms supporting probiotic effects on such complex interactions within the gut cannot be exhaustively investigated *in vitro*, whereas *in vivo* models should preferably be adopted.

In the context described above, here we set out to preliminarily define *in vivo* the impact of the intake of three bacterial strains on the host intestine through a pilot study performed in a mouse model. Specifically, we gavaged wild type mice with the viable cells of three bacterial strains representative of the taxonomic groups most commonly used as probiotics, including *L. paracasei* DG, a strain employed in commercial preparations available in Europe, America, and Asia, which has been demonstrated to efficiently survive the gastrointestinal (GI) transit in adult and pediatric populations (Arioli et al., 2018; Radicioni et al., 2019), affect the gut microbiota taxonomic composition and butyrate levels in healthy adults and irritable bowel syndrome (IBS) patients (Ferrario et al., 2014; Cremon et al., 2018), modulate the immune response in IBS and ulcerative colitis patients (D’Inca et al., 2011; Cremon et al., 2018), and maintain remission of diverticular disease (Tursi et al., 2008, 2013). In addition, we tested *Lactobacillus helveticus* MIMLh5, a bacterial strain of dairy origin used in commercial paraprobiotic formulations available in Europe, which was shown *in vitro* to display antagonistic activity against pathogens and immunomodulatory properties on epithelial and antigen presenting cells (Guglielmetti et al., 2010; Taverniti et al., 2012, 2013, 2019); in addition, MIMLh5 was shown during a clinical trial to contribute to the prevention of recurrent upper respiratory tract infections in adults and children with recurrent rhinopharyngitis (Cavaliere et al., 2020). Finally, *B. bifidum* strain MIMBb23sg has been studied here as a representative member of its species, which possesses marked *in vitro* ability to efficiently adhere to Caco-2 enterocytes, utilize mucin as its sole carbon source, catabolize human milk oligosaccharides, and modulate cytokine expression in bone marrow derived dendritic cells and Caco-2 enterocytes. The biomass of the three selected bacterial strains was administered to mice for 5 days; afterward, the microbiota and host’s gene expression in different intestinal sites were analyzed to gain preliminary information for the elaboration of hypotheses on the mechanisms that support the probiotic-host crosstalk, to be further investigated in subsequent studies.

## Materials and Methods

### Bacterial Strains and Growth Conditions

*Lactobacillus helveticus* MIMLh5 (DSM 32422), *Lacticaseibacillus paracasei* (formerly *Lactobacillus paracasei*) DG (CNCM I-1572), and *Bifidobacterium bifidum* MIMBb23sg (DSM 32708) were grown in de Man-Rogosa-Sharpe (MRS) broth (Difco Laboratories Inc., Detroit, MI, United States), with the addition of 0.05% L-cysteine hydrochloride (Sigma-Aldrich, Merck Millipore, Darmstadt, Germany) for *B. bifidum* (cMRS). Bacterial strains were inoculated from frozen glycerol stocks and sub-cultured twice in MRS or cMRS using a 1:100 inoculum. Incubation temperature was 37°C in microaerophilic conditions for *L. helveticus* and *L. paracasei*, whereas *B. bifidum* was grown in anaerobic jars with the use of Anaercocult A^®^ strips (Merck Millipore). Mice were gavaged with bacterial cells that were freshly prepared from an overnight culture; to this aim, cells were collected, washed twice with sterile phosphate buffered saline (PBS, pH 7.4), and then resuspended in PBS at the concentration of 5 × 10^9^ cells/ml, calculated by using a Neubauer Improved counting chamber (Marienfeld, Lauda-Königshofen, Germany).

### Mice Treatment With Probiotic Strains

Animal experiments were carried out in accordance with the guidelines established by the European Community (n. 2007/526/CE) and by the Italian regulation DL 26/2014 on the accommodation and care of animals used for scientific purposes. Two-month-old female C57BL/6 mice (Charles River, Lecco, Italy) were housed in the conventional animal facility of the Department of Agricultural and Environmental Sciences of the University of Milan. Animals were bred with a regular 12 h light/dark cycle at a constant room temperature of 21 ± 2°C and relative humidity of approximately 55 ± 10%. Animals were housed in standard mouse cages (*n* = 5 per group). After 1-week adaptation, each group of mice received either bacterial suspensions or the vehicle (sterile PBS) for 5 days. Bacterial cells of *L. helveticus* MIMLh5, *L. paracasei* DG, or *B. bifidum* MIMBb23sg were administered *via* oral gavage as a 200 μl suspension. Sterile PBS, used as vehicle in the control groups, was administered with the same procedure. Mice were euthanized 4 h after the fifth gavage. Following sacrifice, two biopsies of the distal ileum, cecum, and proximal colon were collected from each mouse and stored at −80°C for DNA extraction (microbiomic analyses) or preserved in 1 ml of RNA*later* (Qiagen, Hilden, Germany) immediately after cleaning from the intestinal content by flushing the tissue with a syringe containing sterile PBS and stored at −80°C. All steps have been performed on cold trays. After 24 h storage at 4°C, RNA*later* was removed from tubes containing biopsies, which were then stored at −80°C until RNA extraction (gene expression analyses).

### Nucleic Acid Isolation From Intestinal Biopsies

DNA was obtained from mouse biopsies by means of a PowerFecal^®^ DNA Isolation Kit (MO BIO Laboratories Inc., San Diego, CA, United States). The homogenization of mouse biopsies was performed by using a Precellys bead beater (three times for 30 s at 6,800 rpm; Advanced Biotech Italia s.r.l., Seveso, Italy). After that, DNA isolation was conducted following manufacturer’s instructions. For RNA isolation, flushed biopsies stored at −80°C were immediately resuspended in Qiazol (Qiagen) and homogenized by using an IKA T10 basic Ultraturrax (IKA^®^-Werke GmbH & Co. KG, Staufen, Germany; 30,000 rpm for 30 s) while keeping the tubes on ice. Following steps of RNA extraction were performed by using RNeasy Lipid Tissue Mini Kit (Qiagen), in accordance with manufacturer’s instructions. Concentration and purity of nucleic acids were determined with the Take3 Micro-Volume (BioTek Instruments GmbH, Bad Friedrichshall Germany).

### Microbiomic Analyses

To define the bacterial community structure in different intestinal sites, the total DNA extracted from intestinal biopsies was used for 16S ribosomal RNA gene profiling with Illumina MiSeq System at the Center for life—Nanoscience, Istituto Italiano di Tecnologia (Roma, Italy). Briefly, a DNA fragment encompassing the V3 and V4 regions of the 16S rRNA gene was amplified with the primer pair described in Klindworth et al. (2013) creating an amplicon of approximately 460 bp. The library preparation followed the Illumina protocol, adding Illumina adapters and dual-index barcodes (Nextera XT indices) to the amplicon target. Sequence reads (in total 18,361,216 reads were generated, with an average of 155,603 per sample) were analyzed by means of the pipeline Quantitative Insights Into Microbial Ecology (QIIME) version 1.7.0. The QIIME pipeline was adopted, starting from the multiple_join_paired_ends.py until the pick_closed_reference_otus.py script using GreenGene (gg_13_5) as reference taxonomic database. The quality check, and the filtering and trimming were carried out following the default values eventually obtaining 1,951,246 reads with average of 33,072 and median of 29,975 reads per sample. Bacterial abundances in each sample were analyzed until the operational taxonomic unit (OTU) level. Sequence reads from 16S rRNA gene profiling have been deposited in the European Nucleotide Archive (ENA) of the European Bioinformatics Institute under the accession codes PRJEB25821 and PRJEB44459.

### Quantification of Bacterial Cells in Mouse Biopsies by Quantitative PCR (qPCR)

The DNA isolated from the intestinal tracts of mice was employed to quantify bacterial cell number through qPCR by using specific primer pairs. For *L. helveticus* MIMLh5, species-specific primers designed on a portion of β-galactosidase gene were used (Muyzer et al., 1993) (HELV primer; Supplementary Table 1). For the quantification of *L. paracasei* DG, we used primers targeting a region of the DG’s major plasmid (8F-8R1 oligos; Supplementary Table 1). For the quantification of *B. bifidum* MIMBb23sg, we used primers targeting the *bopA* gene (Guglielmetti et al., 2008) (BopA oligos; Supplementary Table 1). To validate primer specificity, we tested HELV, 8F-8R1, and BopA oligos on bioptic DNA isolated from the group of mice that received only PBS; as we did not find any amplification, we employed HELV, 8F-8R1, and BopA primers to specifically detect the presence of MIMLh5, DG, and MIMBb23sg bacterial strains in treatment groups. The total number of bacteria was also quantified by using panbacterial primers 357F-907R targeting the V3-V5 region of the 16S rRNA gene (Muyzer et al., 1993) (EUB oligos; Supplementary Table 1). Calibration curves were set up by extracting DNA from a known number of bacterial cells (namely, 5 × 10^9^ cells from *L. helveticus* MIMLh5 for HELV oligos, *L. paracasei* DG for 8F-8R1 oligos, and *B. bifidum* MIMBb23sg for BopA oligos, and from a mixture of cells of *Escherichia coli*, *L. helveticus*, *L. paracasei*, *B. bifidum*, and *Streptococcus thermophilus* for panbacterial primers). Bacterial DNA isolation was carried out with the same kit employed to extract DNA from mouse biopsies. Afterward, serial decimal dilutions were prepared from each bacterial DNA (starting from a total amount of 150 ng in reaction), and the *C*_*T*_ obtained in qPCR in correspondence of each dilution was correlated with a bacterial cell number, starting from the known amount employed for the extraction. The equation obtained from the calibration curve was then employed to calculate the number of bacterial cells in the DNA isolated from the biopsies based on the obtained *C*_*T*_ value. The cycling parameters used for HELV, 8F-8R1, BopA, and EUB were initiated by 3 min at 95°C, followed by 44 cycles of 10 s at 95°C, 30 s at 58°C, and 30 s at 72°C using the Bio-Rad CFX96 system.

### Gene Expression Analyses

Total RNA concentration was quantified by NanoDrop ND-1000 UV–vis spectrophotometer (NanoDrop Technologies Inc.). The purity of RNA (A_260_/A_280_) was ∼2, and the integrity was checked by loading 100 ng of RNA on a 1% agarose gel in non-denaturing conditions. Genomic DNA was eliminated using DNase I (Sigma-Aldrich), and the reverse transcription reaction was carried out on 1μg RNA using iSCRIPT cDNA SYNTESIS Kit (BioRad). Sufficient cDNA was prepared to run all selected genes. In detail, quantitative reactions were performed in 15 μl of Eva Green mix (BioRad) and 300 nM of ZONU, 5HTR3, and 5HTR4 primers and 500 nM of other primers (Supplementary Table 2) on Bio-Rad CFX96 qPCR instrument. In order to assess PCR efficiency using a relative standard curve, dilution series were prepared by performing threefold serial dilutions starting from one control and one treatment sample. Each sample was tested in duplicate. GAPDH was used as reference gene (Tomas et al., 2016). The thermal profile was the same for each target gene, as follows: 95°C for 3 min, 44 cycles of 95°C for 10 s, 58°C (55.5°C for TPH1 primers) for 30 s and 72°C for 5 s; for melting curve construction, 55°C for 60 s and 80 cycles starting to 55°C and increasing 0.5°C each 10 s. The relative quantification of genes of interest was carried out after sample normalization using the reference gene; tissues of control samples were used as reference samples.

### Statistical Analysis

Statistical calculations were performed using the software program GraphPadPrism 5. The significance of the results was analyzed by unpaired Mann-Whitney test with two-tailed distribution. *P-*value < 0.05 was significant. Differences in microbiota composition between the groups of mice have been determined using Wald test following DESeq2 read counts normalization, considering padj (i.e., *p*-value adjusted with the Benjamini-Hochberg method) < 0.05 for statistical significance. Microbial composition differences between groups have been also defined through LDA Effect Size (LEfSe) considering alpha of 0.5 and log_10_ LDA score > 2 (Segata et al., 2011).

### Ethics Statement

The experimental protocol was approved by the Committee on the Ethics of Animal Experiments of the University of Milan (authorization n. 68/14) and by the Italian Ministry of Health.

## Results

### Probiotics Modified the Bacterial Load in Different Mouse Intestinal Sites

**FIGURE 1:**
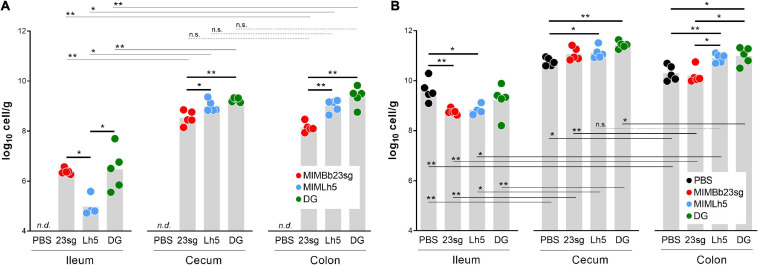
Bacterial quantification by qPCR in mouse intestinal sites. **(A)** qPCR experiments performed with the respective strain-specific primers. **(B)** qPCR experiments performed with pan-bacterial primers. PBS, control mice gavaged with phosphate buffered saline; 23sg, mice gavaged with *B. bifidum* MIMBb23sg; Lh5, mice gavaged with *L. helveticus* MIMLh5; DG, mice gavaged with *L. paracasei* DG. *n.d.*, target not detected with any of the strain-specific primer pairs. Statistics is according to Mann-Whitney *U*-test; **P < 0.01; *P < 0.05; n.s., not significant.

qPCR was used to quantify *B. bifidum* MIMBb23sg, *L. helveticus* MIMLh5, and *L. paracasei* DG in the ileum, cecum, and colon of mice gavaged once daily for 5 days with the bacterial cells or PBS. As expected, qPCR resulted negative in samples from PBS-gavaged mice. Conversely, we quantified 6.4, 8.5, and 8.1 log_10_ cells/g (median values), respectively, in the ileum, cecum, and colon of MIMBb23sg-gavaged mice, 4.8, 8.9, and 9.2 log_10_ cells/g, respectively, in the ileum, cecum, and colon of MIMLh5-gavaged mice, and 6.5, 9.3, and 9.5 log_10_ cells/g, respectively, in the ileum, cecum, and colon of DG-gavaged mice (Figure 1A). All strains colonized more the cecum and colon than the ileum. Furthermore, strains MIMBb23sg and DG were significantly more abundant than MIMLh5 in the ileum, whereas strains DG and MIMLh5 were more abundant than MIMBb23sg in the cecum and colon (Figure 1A).

Subsequently, we performed qPCR with panbacterial primers targeting the 16S rRNA gene to quantify the total bacterial cells in the same mouse intestinal samples used for the quantification of probiotic cells. Total bacterial cell concentration was higher in the cecum and colon than in the ileum (Figure 1B). Significant higher concentrations were also found in the cecum compared to the colon for PBS-, DG-, and MIMBb23sg-gavaged mice (Figure 1B). In addition, we found a significant reduction of the bacterial concentration in the ileum of mice that received MIMLh5 and MIMBb23sg (8.7 and 8.8 log_10_ cells/g, respectively) compared to the PBS-gavaged mice (9.6 log_10_ cells/g); conversely, the administration of MIMLh5 and DG induced a significant increase of the bacterial load in the cecum compared to PBS- and in the colon compared to both PBS- and MIMBb23sg-gavaged mice (Figure 1B).

### Probiotics Differently Modulated the Microbiota of Ileum, Cecum, and Colon

To better elucidate the impact of MIMBb23sg, MIMLh5, and DG intake on the gut microbiota composition, we performed 16S rRNA gene profiling on the same intestinal samples used in qPCR experiments. The analysis of intra-sample (α) diversity revealed a significantly higher taxonomic richness in the cecum and colon compared to the ileum in all mouse groups (Supplementary Figure 1). Bacterial richness in the cecum was also higher than that in the colon for all groups except the group of mice supplemented with strain MIMLh5. In addition, exclusively in the cecum, the Faith’s Phylogenetic Diversity and the Shannon indexes were higher than control mice for MIMLh5- and MIMBb23sg-gavaged mice, whereas Chao1 index was higher than control for MIMLh5-gavaged mice (Supplementary Figure 1).

**FIGURE 2:**
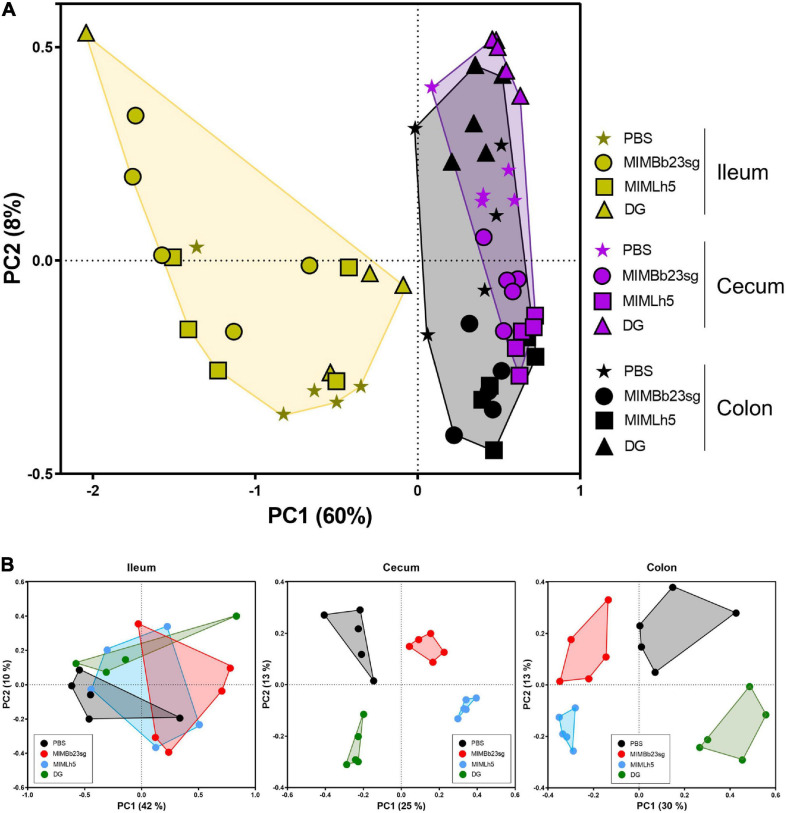
Inter sample (β-) diversity of the microbiota in mouse intestinal sites shown as principal coordinates analysis of generalized UniFrac distances (α = 0.5) based on OTU abundances. Every symbol in the graphs refers to a single mouse. The first two coordinates (PC1 and PC2) are displayed with the percentage of explained variance in brackets. The analysis was performed with sample considered all together **(A)** or separated by intestinal site **(B)**.

Subsequently, we carried out inter-sample (β) diversity analysis through generalized UniFrac (alpha = 0.5), which revealed that ileum samples clustered separately from cecum and colon samples (Figure 2A). In addition, whereas in the ileum the samples did not group according to the treatment, in the cecum and colon the samples of each specific treatment group segregated distinctly from those of the other groups (Figure 2B).

Next, LEfSe analysis was used to compare the abundance of bacterial taxa of PBS- and probiotic-treated mice in the three different intestinal sites. Cumulatively, the results showed that most of the differences in bacterial taxonomic composition between probiotic- and PBS-treated mice were in the cecum (Supplementary Figure 2). Considering the bacterial strains individually, the bacterial taxonomic structure of MIMLh5- and DG-gavaged mice differed from control mice mostly in the cecum (Supplementary Figure 3). On the contrary, MIMBb23sg-treated mice displayed marked differences from control mice also in the ileum (Supplementary Figure 3). The most evident difference between the microbiota of mice gavaged with probiotics and controls referred to taxa of the *Bacteroidetes* family S24-7, which were significantly enriched in the cecum of all probiotic-treated mice (Supplementary Figure 3). The S24-7 bacteria resulted significantly increased also in the colon of MIMLh5- and DG-gavaged mice, and significantly decreased in the ileum of MIMBb23sg- and MIMLh5-treated mice (Supplementary Figure 3).

**FIGURE 3:**
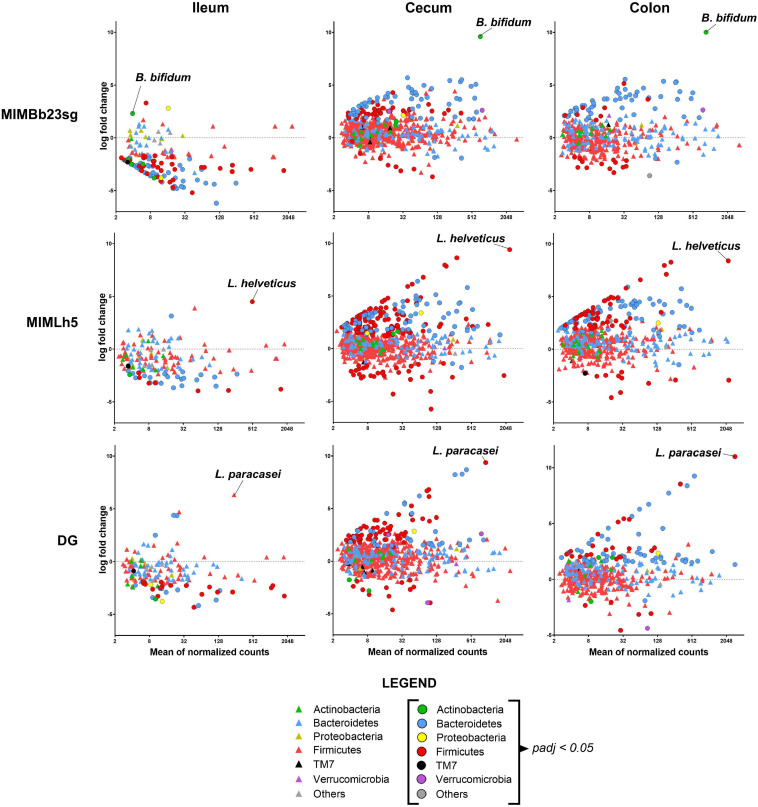
Differences in microbiota composition between PBS-treated mice and the three groups of mice gavaged with a probiotic strain in the different intestinal sites. Each symbol (circle or triangle) refers to a single OTU. Symbols’ color indicates OTU’s phylum, as described in the legend. OTUs with a significantly different abundance between groups (i.e., padj < 0.05) are indicated with circles.

Subsequently, we adopted the DESeq2 negative binomial distribution method in the analyses of microbiomic data to infer differential relative abundances at the OTU level between PBS- and probiotic-gavaged mice. More specifically, we found that OTUs’ normalized abundance was mostly decreased in the ileum and increased in cecum and colon in all groups of mice gavaged with the probiotic strains (Figure 3). In detail, 82 OTUs resulted significantly reduced in the ileum of MIMBb23sg- compared to PBS-treated mice, mostly belonging to the *Bacteroidetes* family S24-7 and to the Firmicutes genus *Lactobacillus*, whereas only three OTUs were overrepresented, including the OTU putatively corresponding to the strain MIMBb23sg (Supplementary Figure 4A). Conversely, 32 OTUs were significantly modified by strain MIMLh5 in the ileum; specifically, 22 S24-7 and 7 *Lactobacillus* OTUs decreased, while only two increased, including the OTU putatively corresponding to the strain MIMLh5 (Supplementary Figure 4B). Concerning the strain DG, 30 OTUs were decreased in the ileum mostly belonging to the Bacteroidetes family S24-7 and to the Firmicutes genus *Lactobacillus*, whereas only four OTUs were overrepresented (Supplementary Figure 4C).

In the cecum and colon of MIMBb23sg-treated mice, 101 and 59 OTUs were significantly higher, and 8 and 15 OTUs lower, respectively. Mice gavaged with strain MIMLh5 had 178 and 112 OTUs significantly increased, and 32 and 13 significantly decreased OTUs, in the cecum and colon, respectively. DG-gavaged mice had 93 and 76 significantly increased OTUs, and 14 and 8 significantly decreased OTUs, in the cecum and colon, respectively (Figure 3 and Supplementary Figure 4). Most of the OTUs increased in the cecum and colon of all probiotic-treated mice belonging to the S24-7 family. In the cecum of probiotic-treated mice, also numerous OTUs of the order Clostridiales were significantly higher than control mice (Supplementary Figure 4). Furthermore, several *Lactobacillus* OTUs were changed in MIMLh5-gavaged mice, whereas two OTUs ascribed to the species *Akkermansia muciniphila* were increased in MIMBb23sg-treated mice (Supplementary Figure 4). Notably, several overrepresented OTUs in the cecum and colon of probiotic-treated mice were also found to be reduced in the ileum of probiotic-treated mice (Supplementary Figure 4), suggesting a possible relocation of these bacteria from the ileum toward distal bowel districts.

### Probiotics Differently Influenced the Mucosal Gene Expression in the Ileum, Cecum, and Colon

Probiotics may affect the host’s gene expression in the gut both directly and indirectly through the modulation of the microbiota composition. In this study, we observed significant differences in microbiota composition between probiotic- and PBS-gavaged mice in all three intestinal sites investigated. Therefore, we instigated the expression of several genes in biopsies of mouse mucosa from ileum, cecum, and colon. In particular, we assessed the local immune response, epithelial permeability, and intestinal motility by RT-qPCR targeting the genes coding several cytokines (IL-10, IL-1β, IL-6, TGF-β, and TNF-α), cyclooxygenase (COX)-2, inducible nitric oxide synthase (iNOS), and zonulin. In addition, we investigated the expression of genes involved in serotonin metabolism: the *TPH1* gene coding for tryptophan hydroxylase-1, which is the rate-limiting step of serotonin biosynthesis (Lovenberg et al., 1967), the gene coding for serotonin reuptake transporter SERT, which is widespread in all intestinal epithelial cells of intestinal mucosa (Chen et al., 1998), and the genes coding the functional receptors for serotonin 5HTR3 and 5HTR4.

**FIGURE 4:**
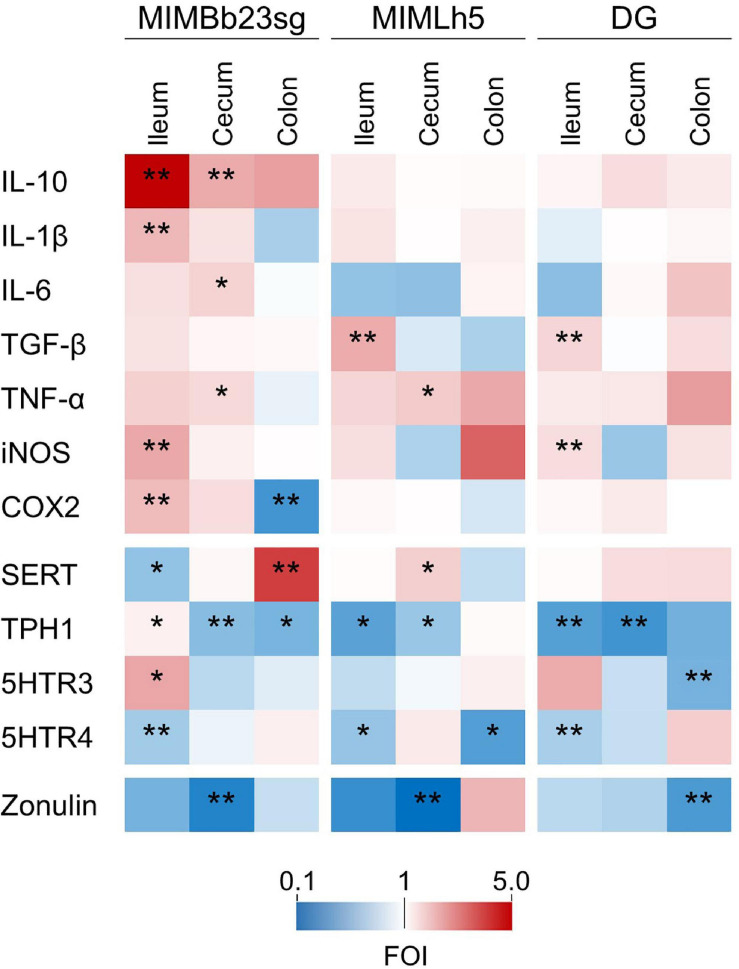
Heatmaps summarizing the results of gene expression analysis performed by RT-qPCR to identify genes whose expression was significantly modulated by the treatment with probiotic strains compared to the control condition (gavage with PBS). The fold of induction (FOI) for each gene is displayed as color ranging from blue to red as shown in the key. Asterisks indicate statistically significant differences according to Mann-Whitney *U*-test; **P < 0.01; *P < 0.05.

Concerning the genes directly involved in the mucosal immune response, most of the significant differences were observed in the ileum, where MIMBb23sg-gavaged mice displayed a significant increase in the expression of IL-10 (median FOI = 5.0), IL-1β (FOI = 2.1), iNOS (FOI = 2.4), and COX2 (FOI = 2.1) compared to control mice (Figure 4 and Supplementary Figure 5). In addition, the expression of TGF-β was increased in the ileum of MIMLh5- and DG-treated mice (FOIs 2.3 and 1.9, respectively). DG-gavaged mice were also associated with an significant ileal overexpression of iNOS compared to control (FOI 1.5). In the cecum, MIMBb23sg-gavaged mice were characterized by a significant increased expression of IL-10 (FOI = 2.3), IL-6 (FOI = 1.7), and TNF-α (FOI = 1.6). On the other hand, in the cecum, MIMLh5-treated mice had a significantly higher expression of TNF-α (FOI = 1.8), whereas the gene expression in DG-gavaged mice was not significantly different from control mice (Figure 4 and Supplementary Figure 5).

Compared to control mice, probiotic-treated mice also displayed a significant modification in the expression of genes involved in the serotonergic metabolism. In particular, in MIMBb23sg-gavaged mice, SERT gene expression was downregulated in the ileum (FOI = 0.6) and upregulated in the colon (FOI = 4.0); TPH1 was upregulated in the ileum (FOI = 1.2) and downregulated in the cecum and colon (FOIs 0.6 and 0.5, respectively); moreover, in the ileum, the expression of genes coding the serotonin receptors 5HTR3 and 5HTR4 was, respectively, increased (FOI = 2.4) and decreased (FOI = 0.7) (Figure 4 and Supplementary Figure 5). Compared to controls, mice gavaged with strain MIMLh5 displayed a significant reduction of TPH1 gene expression in the ileum (FOI = 0.4) and the cecum (FOI = 0.6), 5HTR4 in the ileum (FOI = 0.6) and the colon (FOI = 0.4), and a significant increase of SERT in the cecum (FOI = 1.8). Similarly to MIMLh5, mice gavaged with strain DG displayed a significant downregulation of genes TPH1 in the ileum (FOI = 0.4) and cecum (FOI = 0.3), and 5HTR4 in the ileum (FOI = 0.7), in addition to 5HTR3 (downregulated in the colon; FOI = 0.5) (Figure 4 and Supplementary Figure 5).

Finally, the mucosal gene expression of zonulin, a potential marker of epithelial barrier integrity, was significant reduced in all probiotic-treated mice in the cecum (for strains MIMBb23sg and MIMLh5; FOI 0.24 and 0.11, respectively) and colon (strain DG; FOI 0.38) (Figure 4 and Supplementary Figure 5).

**FIGURE 5:**
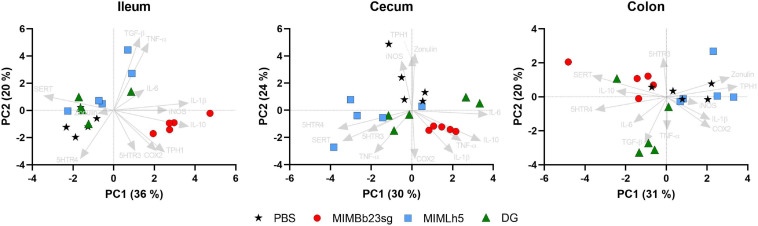
Bi-plot of the principal components analysis (PCA) showing the loading of gene expression data (arrows) and the scores of each mouse (symbols).

Subsequently, gene expression data were used for principal component analyses (PCAs) in each intestinal site. The most evident result emerging from the obtained PCA biplots was that MIMBb23sg-gavaged mice clustered separately in ileum and cecum, according to the direction of the IL-10 vector (Figure 5). In addition, the SERT vector direction was opposite to MIMBb23sg-treated mice in the ileum, whereas it was in the same direction as MIMBb23sg-gavaged mice in the colon (Figure 5).

## Discussion

Probiotic microorganisms may affect the host’s health through diverse mechanisms that can be roughly categorized in two main classes: (i) interaction with the intestinal microbial ecosystem (Singh et al., 2013; Gargari et al., 2016) and (ii) contribution to the host’s mucosal homeostasis in the gut, especially in terms of immunomodulation (Guglielmetti et al., 2014; Kawahara et al., 2017; Taibi et al., 2017; Tanaka et al., 2017) and preservation of barrier function (Seth et al., 2008; Khailova et al., 2009; Karczewski et al., 2010). Here, we investigated both aspects *in vivo* in a mouse model.

It is commonly accepted that the ability of a probiotic to influence host health first stems from its capacity to affect the microbial ecology of the gut (Hill et al., 2014). Accordingly, the initial step of this study consisted in characterizing the impact of the administration of three probiotic strains on the microbiota composition in different murine intestinal districts. All bacterial strains investigated affected the microbiota of the three enteric sites in terms of both bacterial composition and load. Of note, we found that the bacterial cell concentration in the ileum was significantly lower in mice that received treatment with *L. helveticus* MIMLh5 and *B. bifidum* MIMBb23sg than mice gavaged with PBS. This trait, if confirmed in humans, might be of interest, as the amount of bacterial cells in the small intestine has to be constantly controlled by the host and its excessive expansion leads to detrimental health consequences that are collectively defined as “small intestine bacterial overgrowth” (SIBO) syndrome (Sherman and Lichtman, 1987). A meta-analysis of 18 clinical trials suggested that probiotic supplementation may be effective in the treatment of SIBO, reducing H_2_ production and abdominal pain (Zhong et al., 2017).

Most of the observed differences between probiotic-treated and control mice referred to OTUs ascribed to the Bacteroidales family S24-7. The taxon S24-7 includes largely uncultured and poorly characterized bacteria that are highly specific for the intestine of animals, including humans (Ormerod et al., 2016; Thompson et al., 2017). S24-7 bacteria have been often found as dominant members of the intestinal microbiota of laboratory mice, where these microorganisms were reported to be lost during osmotic diarrhea (Tropini et al., 2018) and significantly modulated by dietary interventions (Serino et al., 2012; Garcia-Mazcorro et al., 2018). In our study, numerous S24-7 OTUs were lower in the ileum and overrepresented in the cecum and colon of probiotic-supplemented mice, confirming that these bacteria easily respond to external perturbations. S24-7 bacteria have been also associated with treatment-induced remission of experimental colitis in mice (Rooks et al., 2014), suggesting that members of this family may plausibly impact on gut function and health (Ormerod et al., 2016); nonetheless, the consequences of changes in the intestinal abundance and localization of S24-7 bacteria are at present unknown.

In this study, we separately analyzed three intestinal sites, which possess different anatomical and functional properties: the colonic and cecal epithelium is much more densely populated by bacteria and is covered by a thick double mucus layer that protects from lumen bacteria and antigens, whereas the small intestine harbors much less bacteria and is covered by a thinner and looser mucus layer that allows a continuous contact with food antigens and enteric commensal microorganisms (Santaolalla et al., 2011). Accordingly, it was shown that the gut microbiota modulates the gene expression mostly in the mucosa of the small intestine (Larsson et al., 2012). Also in our study, the expression of genes involved in immune response was mostly modulated in the ileum (especially in mice gavaged with the human intestinal symbiont *B. bifidum*), suggesting that the small bowel is plausibly the privileged site where probiotics may exert the immunomodulation and, in general, direct crosstalk with the host (Derrien and van Hylckama Vlieg, 2015).

We found that *B. bifidum* MIMBb23sg could exert a potential anti-inflammatory effect, as suggested by the marked overexpression in the ileum of the gene coding the regulatory (anti-inflammatory) cytokine IL-10. Furthermore, in the colon of *B. bifidum*-treated mice, we observed an evident downregulation of cyclooxygenase (COX)-2, which is known to contribute to colonic inflammation in inflammatory bowel disease (IBD) (Singer et al., 1998).

Other significant modulations of the expression of genes involved in intestinal immune responses were observed. *L. helveticus* MIMLh5 induced an increase of TNF-α in the cecum, which partly confirms the immunostimulatory attitude of this bacterium observed *in vitro* (Taverniti et al., 2012, 2013, 2019). In addition, *B. bifidum* MIMBb23sg and *L. paracasei* DG impacted on the gene expression of iNOS in the ileum, an effect already observed for DG in *ex vivo* investigations on colonic biopsies (Compare et al., 2017). This enzyme is involved in the synthesis of nitric oxide, whose production has both beneficial and detrimental consequences, depending on the physiologic environment and magnitude of expression (Lind et al., 2017). Nonetheless, the constitutive presence of iNOS in normal ileal epithelium indicates a role in maintaining intestinal homeostasis for this enzyme (Hoffman et al., 1997), which was also demonstrated to be involved in villous re-epithelialization of mucosa upon injuries (Gookin et al., 2002).

In our model, we observed that the bacterial load of mice gavaged with probiotics, in addition to being decreased in the ileum, was greater in the distal enteric sites. Several OTUs whose abundance was significantly decreased in the ileum, in contrast, were significantly increased in the cecum and colon, and this was observed for all three probiotic strains. To assess the hypothesis that the observed re-location of these bacterial taxa along the intestinal tract may derive from the stimulation of small bowel motility, we analyzed the modulation of serotonergic gene expression. Mucosal release of serotonin stimulates both intrinsic sensory neurons (most likely *via* 5-HT4 receptors) affecting peristalsis, secretion and vasodilation, and extrinsic sensory neurons (*via* 5-HT3 receptors), affecting gastric emptying, pancreatic secretion, satiation, pain, discomfort, and nausea (Mawe and Hoffman, 2013). In our study, *L. helveticus* MIMLh5 and *L. paracasei* DG decreased the expression of genes coding TPH1 (in the ileum and cecum) and the serotonin receptors (reduction of 5HTR3 in the colon and reduction of 5HTR4 in the ileum by DG, and reduction of 5HTR4 in the ileum and colon by MIMLh5), while inducing an increased expression of the SERT-coding gene. These data suggest that MIMLh5 and DG supplementation may reduce serotonin levels and/or activity in the gut. Similar results were reported for LAB species such as *Limosilactobacillus reuteri* and *Lacticaseibacillus casei* in dysbiotic mice (Beck et al., 2019). Since a role for serotonin has been proposed in the pathophysiology of different GI disorders (Manocha and Khan, 2012; Mawe and Hoffman, 2013), pharmacological interventions have been based on the use of agonists and antagonists of serotonin receptors, particularly 5HTR4 and 5HTR3 (Coates et al., 2017). The 5HTR4 antagonist SB-207266-A was shown to inhibit serotonin-evoked contraction of human isolated terminal ileum circular muscle while reducing the serotonin-induced inhibition of spontaneous contractions of human sigmoid colon circular muscle (Sanger et al., 1998). Another study based on the use of the same 5HTR4 antagonist in subjects with diarrhea-predominant IBS demonstrated an improvement in small bowel transit to a normal time and reduction of rectal sensitivity (Houghton et al., 1999). However, since a single receptor acts in different systems due to its multiple sites of action, the use of pharmaceutical intervention may often cause off-target or anti-target effects, which result in side-effects, as observed for some molecules targeting the blockade of 5-HTR4 and 5-HTR3 (De Ponti, 2013). In the context of milder dysfunctions, specific probiotic treatments may be evaluated for a potential action in the relapse of symptoms connected to altered motility (Dimidi et al., 2017).

Differently from LAB, in our study, the administration of *B. bifidum* MIMBb23sg induced a significant increase of TPH1 and the downregulation of the gene coding the serotonin reuptake transporter SERT in the ileum, both events potentially resulting in a greater availability of serotonin that may lead to enhanced peristalsis. The effect of enhanced peristalsis through serotonin release was also shown in a Zebrafish model by employing *Bifidobacterium animalis* (Lu et al., 2019). Furthermore, in the ileum, *B. bifidum* MIMBb23sg-gavaged mice were characterized by the downregulation of 5HTR4 and the overexpression of 5HTR3. Yamano et al. (1997) found that the stimulation of 5HTR3 causes a contractile effect in the mouse ileum. Therefore, the induction of 5HTR3 by MIMBb23sg seems to also be in accordance with the potential propulsive effect in the small bowel, which turns in a more regulatory effect in the colon. The results here presented show, in fact, that *B. bifidum* MIMBb23sg administration induced an overexpression of SERT- and a significant downregulation of TPH1-coding genes in the colon, plausibly leading to decreased serotonin levels, which might be a positive outcome in the colonic environment. Reportedly, high levels of serotonin due to SERT downregulation enhance the severity of inflammation (Haub et al., 2010); furthermore, the cytokines TNF-α and IFN-γ, and intestinal inflammation in general, have been reported to keep colonic SERT levels low (te Velde et al., 2007; Tada et al., 2016). SERT has also been proposed to play a role in IBS, since IBS patients have attenuated SERT expression in the gut, associated to decreased serotonin reuptake capacity by enterocytes (Faure et al., 2010; Jin et al., 2016). Accordingly, it was reported that IBS patients are characterized by increased serotonin availability in the colonic mucosa, which, notably, correlates with mast cell counts and abdominal pain severity (Cremon et al., 2011). Therefore, it has been proposed that elevated mucosal serotonin in the colon, which is involved in visceral hypersensitivity (Keszthelyi et al., 2015), may contribute to the generation of IBS symptoms through mechanisms that include mesenteric sensory fiber stimulation and immune activation (Cremon et al., 2011). The observed modulation of SERT and TPH1 gene expression toward a decrease of serotonin availability in the colon may provide a potential mechanistic explanation for the reported ability of *B. bifidum* to alleviate symptoms in IBS patients (Guglielmetti et al., 2011).

We lastly evaluated the impact of probiotics administration on the expression of zonulin, which is a protein responsible for the disengagement of the zonula occludens proteins of the tight junction complex, the major component of epithelial barrier function (Fasano, 2001). Uncontrolled zonulin activity promotes increased permeability [potentially causing leaky gut syndrome (Mu et al., 2017)] that allows the entrance of luminal factors contributing to the onset of chronic inflammatory diseases (Sturgeon and Fasano, 2016). Therefore, the fact that zonulin did not increase in the intestine of mice gavaged daily with a billion viable probiotic cells may suggest the safety of these microorganisms. Furthermore, interestingly, the three probiotic strains here investigated significantly reduced zonulin expression, with a decrease in the colon upon *L. paracasei* DG administration, and in the cecum following administration of *L. helveticus* MIMLh5 and *B. bifidum* MIMBb23sg. The positive effects of probiotic administration on zonulin levels were also reported in human trials (Lamprecht et al., 2012; Liu et al., 2013).

In conclusion, our study suggests that probiotic administration for 5 days to healthy mice of strains *B. bifidum* MIMBb23sg, *L. helveticus* MIMLh5, and *L. paracasei* DG can induce significant effects on diverse compartments of the gut system. The investigated probiotic microorganisms, which belong to three different bacterial taxonomic groups, may share some common features, such as the preferential site of colonization in the gut and the general modulatory effects on the intestinal microbiota (e.g., reduction in the ileum and increase in the cecum and colon of the abundance of several bacterial taxa). On the contrary, the modulation of host’s gene expression was confirmed to be strictly strain-specific (Hill et al., 2014).

This study has several limits, particularly because it is based on a limited number of mice per treatment group (*n* = 5) and because the analyses of host cells’ responses to probiotics have been carried out only at gene expression level. This pilot *in vivo* trial represents only the initial step in the process of understanding the mechanisms supporting probiotic-host crosstalk, and the data here presented cannot permit to draw conclusions. Nonetheless, even though preliminary, this study lays the foundations for subsequent *ad hoc* investigations in humans. In particular, this study provides a rationale for designing trials focusing on the investigation of the effects of probiotics on the serotonergic system, which is a topic still widely unexplored.

## Data Availability Statement

Metataxonomic raw data have been deposited in the ENA online repository under the accession numbers PRJEB25821 and PRJEB44459. The other original contributions presented in the study are included in the article/Supplementary Material, further inquiries can be directed to the corresponding author.

## Ethics Statement

The experimental protocol was approved by the Committee on the Ethics of Animal Experiments of the University of Milan (authorization n. 68/14) and by the Italian Ministry of Health.

## Author Contributions

SG and VT conceived, designed the study, and wrote the manuscript. WF contributed to the design of the study. IT, SA, VC, and VT developed and carried out *in vivo* experiments and including sample collection. CB, CL, VT, and UR performed the qPCR experiments. GG and SG performed the bioinformatic analysis of microbiomic data and the statistical analyses. All authors critically reviewed the manuscript.

## Conflict of Interest

SG was consultant of Sofar S.p.A., the private company that commercializes strains *L. paracasei* DG and *B. bifidum* MIMBb23g. WF was an employee of Sofar S.p.A. SG and VT received royalties from the sale of the strain *B. bifidum* MIMBb23sg. SG and VT received royalties from the sale of the strain *L. helveticus* MIMLh5. The remanining authors declare that the research was conducted in the absence of any commercial or financial relationships that could be construed as a potential conflict of interest.

## Publisher’s Note

All claims expressed in this article are solely those of the authors and do not necessarily represent those of their affiliated organizations, or those of the publisher, the editors and the reviewers. Any product that may be evaluated in this article, or claim that may be made by its manufacturer, is not guaranteed or endorsed by the publisher.
