# Supplementary material for: Probiotics Modulate Mouse Gut Microbiota and Influence Intestinal Immune and Serotonergic Gene Expression in a Site-Specific Fashion
Source: Front Microbiol. 2021 Sep 1;12:706135. doi: 10.3389/fmicb.2021.706135 (PMC8441017; doi:10.3389/fmicb.2021.706135)
Supplement: Supplementary file 1 [file Data_Sheet_1.PDF]

Supplementary Tables

Table S1. Primers used in qPCR experiments for the quantification of bacteria. BopA F/R, *Bifidobacterium bifidum*-specific primers; 357 F/907 R, panbacterial primers. *, primers were designed using Primer3 Tool and checked with OligoAnalyzer 3.1 Tool.

**Primer**	**Sequence** (5' → 3')	**Target**	**Source**
BopA-F	GTGTTCCCACCAACTACA	*bopA* gene of *B. bifidum* (GenBank code: AM710395)	This study*
BopA-R	GATCTGGTCGTAACCAGT		
HELV-F	AAACGGGCATTTTGTGGGCTAT	β-galactosidase gene of *L. helveticus*	This study*
HELV-R	GAAGCTTAAGGTTGAAGATGCC		
8F	GAAGCACTATGACTATGGTG	Major plasmid of strain *L. paracasei* DG	This study*
8R1	CAACTAAGGCCCCTAAATCA		
357F	CCTACGGGAGGCAGCAG	16S rRNA gene (panbacterial)	(Muyzer *et al.*, 1993)
907R	CCGTCAATTCMTTTRAGTTT		

Table S2. Primer used in RT-qPCR to study the gene expression in mouse mucosa biopsies collected from ileum, cecum, and colon. *, primers were designed using Primer3 Tool and checked with OligoAnalyzer 3.1 Tool.

**Primer**	**Sequence (5' → 3')**	**Source**
GAPDH F	ATGACCACAGTCCATGCCATC	This study*
GAPDH R	GGTCCTCAGTGTAGCCCAAG	
SERT F	CAA AACCAAGAACCAAGAG	(Haub et al., 2010)
SERT R	CATAGCCAATGACAGACAG	
5-HTR3 F	GTGATAAGCCTCGCTGAGACC	Adapted from (Chetty et al., 2006)
5-HTR3 R	CGCATCTCATCCCGCTTCT	
5-HTR4 F	GATGCCCTTTGGTGCCAT	This study*
5-HTR4 R	CAGCAGATGGCGTAATACCTG	
TPH-1 F	ATGAGAGAATTTGCCAAGACC	Adapted from (Sato et al., 2004)
TPH-1 R	CGTGAACTATATTTCCCTCAG	
IL10 F	CCCTTTGCTATGGTGTCCTT	(Montbarbon et al., 2013)
IL10 R	TGGTTTCTCTTCCCAAGACC	
IL1β F	CAACCAACAAGTGATATTCTCCATG	
IL1β R	GATCCACACTCTCCAGCTGCA	
TNFa F	TGGGAGTAGACAAGGTACAACCC	
TNFa R	CATCTTCTCAAAATTCGAGTGACAA	
IL6 F	TGTGCAATGGCAATTCTGAT	This study*
IL6 R	CTCTGAAGGACTCTGGCTTTG	
iNOs F	GTGAAGGGACTGAGCTGTTAG	
iNOs R	CACTTCTGCTCCAAATCCAAC	
COX-2 F	CCTTGCTGTTCCATGT	(Taverniti et al., 2013)
COX-2 R	CAGGAAGGGGGTTCAC	
ZONU-F	GCTATGTGGATTGGTT	This study*
ZONU-R	TCCATAGAGCGATGAT	

Supplementary Figures

Fig. S1. Analyses of the intra-sample (α-) diversity of microbiota community structure in mouse intestinal sites, carried out by using four different indexes. Statistics is according to Mann-Whitney U (unpaired) test; **, P<0.01; *, P<0.05; n.s., not significant.

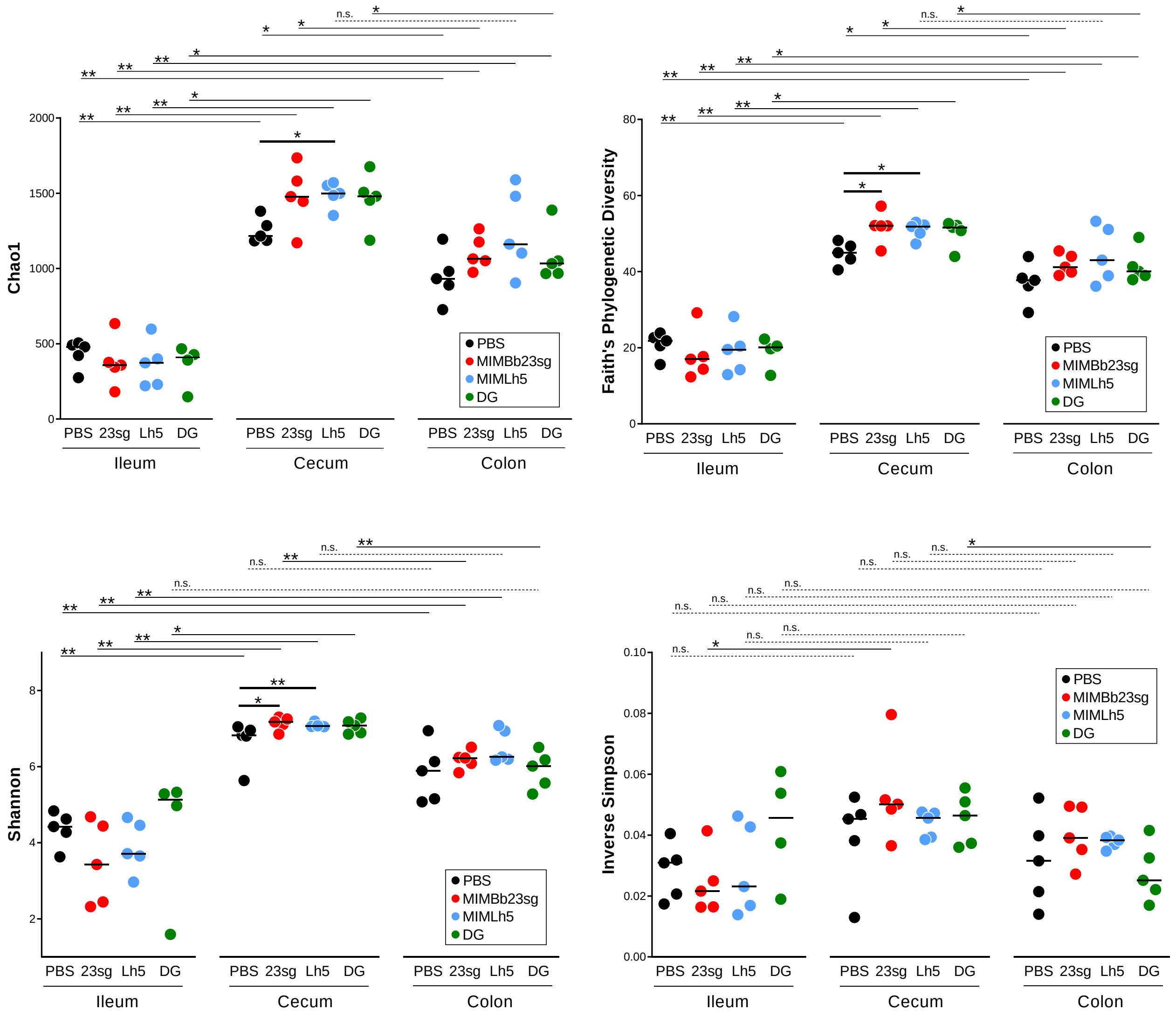

Fig. S2. Most differentially abundant taxa in the three intestinal sites between PBS- and probiotic-gavaged mice identified through Linear discriminant analysis (LDA) effect size (LEfSe) (p<0.05).

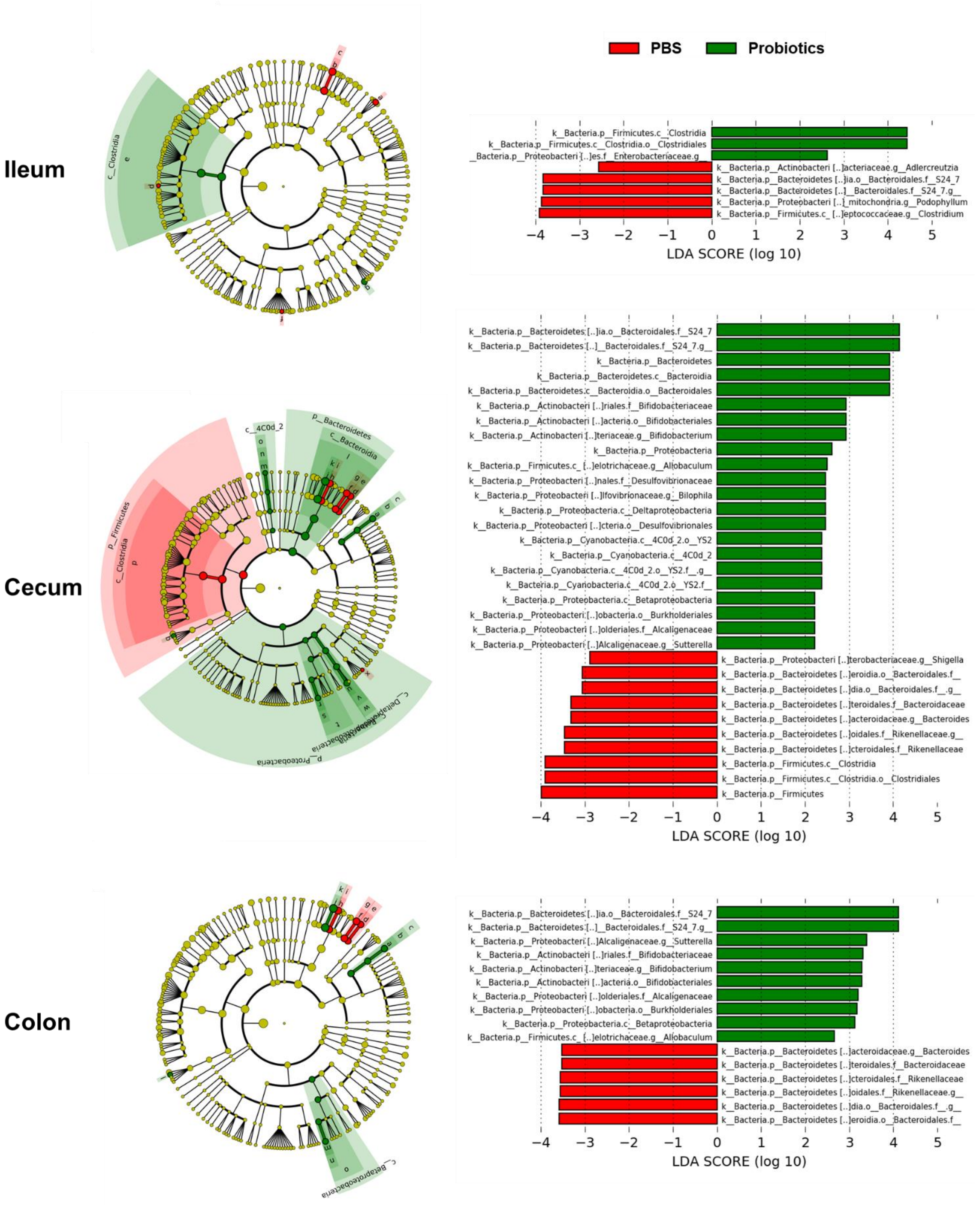

Fig. S3. Graphics of Linear discriminant analysis (LDA) effect size (LEfSe) for mice gavaged with *B. bifidum* MIMBb23sg (panel **A**), *L. helveticus* MIMLh5 (panel **B**) and *L. paracasei* DG (panel **C**) compared to control mice (i.e. mice gavaged with PBS) in three different intestinal sites (ileum, cecum and colon). Positive LDA scores represent taxa significantly ($p < 0.05$) higher in the probiotic-treated mice; negative LDA scores represent taxa significantly ($p < 0.05$) higher in the control mice. The name of the taxon levels are abbreviated as follows: p, phylum; c, class; o, order; f, family; g, genus; s, species.

A

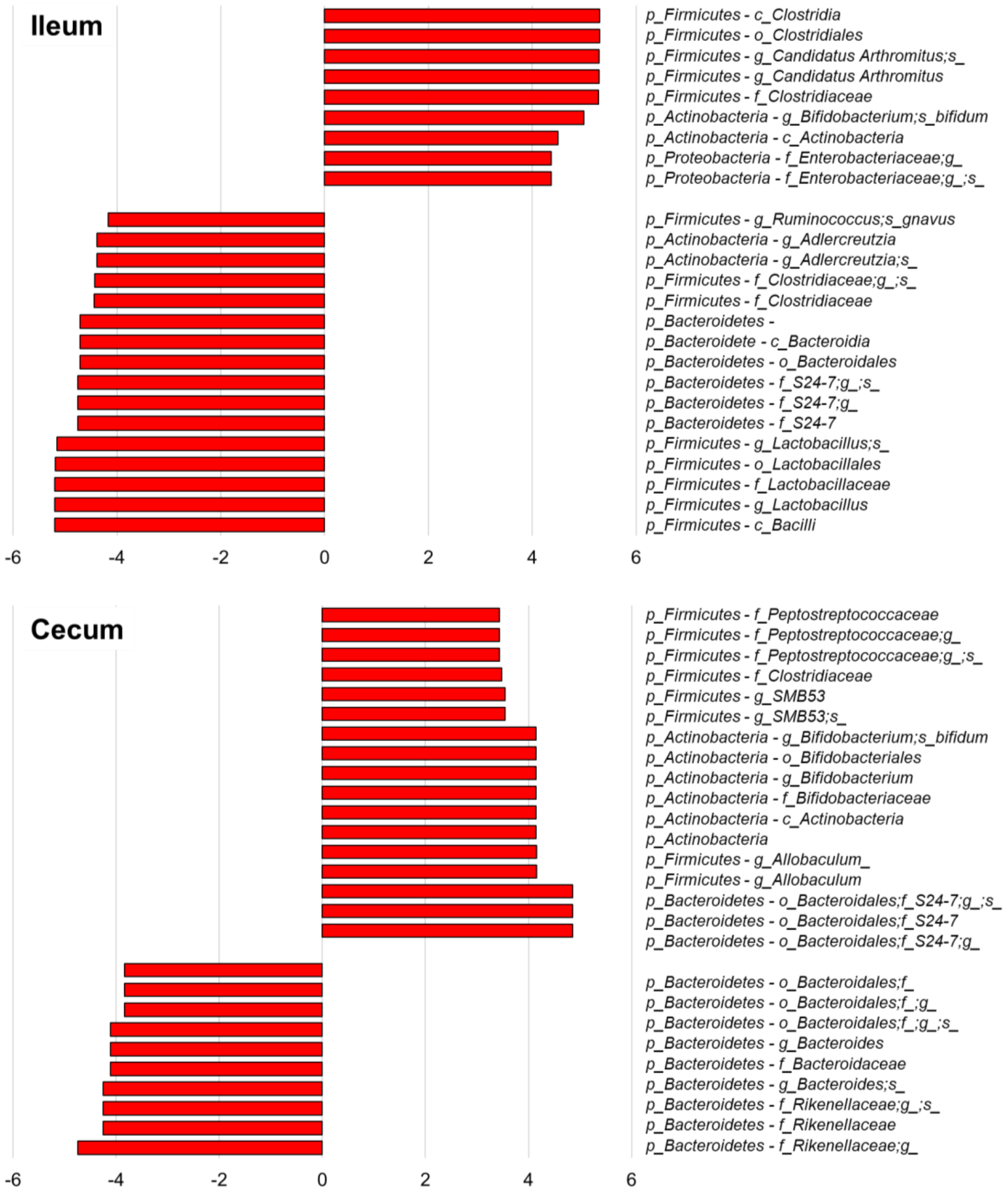

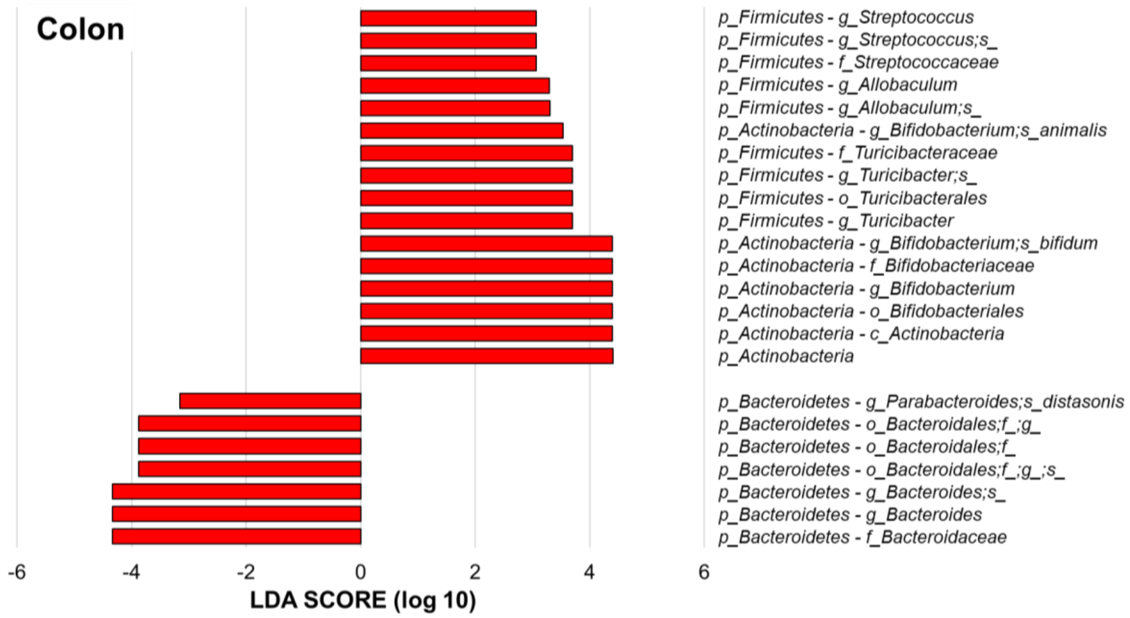
Colon
p_Firmicutes - g_Streptococcus
p_Firmicutes - g_Streptococcus;s_
p_Firmicutes - f_Streptococcaceae
p_Firmicutes - g_Allobaculum
p_Firmicutes - g_Allobaculum;s_
p_Actinobacteria - g_Bifidobacterium;s_animalis
p_Firmicutes - f_Turicibacteraceae
p_Firmicutes - g_Turicibacter;s_
p_Firmicutes - o_Turicibacterales
p_Firmicutes - g_Turicibacter
p_Actinobacteria - g_Bifidobacterium;s_bifidum
p_Actinobacteria - f_Bifidobacteriaceae
p_Actinobacteria - g_Bifidobacterium
p_Actinobacteria - o_Bifidobacteriales
p_Actinobacteria - c_Actinobacteria
p_Actinobacteria
p_Bacteroidetes - g_Parabacteroides;s_distasonis
p_Bacteroidetes - o_Bacteroidales;f_;g_
p_Bacteroidetes - o_Bacteroidales;f_
p_Bacteroidetes - o_Bacteroidales;f_;g_;s_
p_Bacteroidetes - g_Bacteroides;s_
p_Bacteroidetes - g_Bacteroides
p_Bacteroidetes - f_Bacteroidaceae
-6
-4
-2
0
2
4
6
LDA SCORE (log 10)

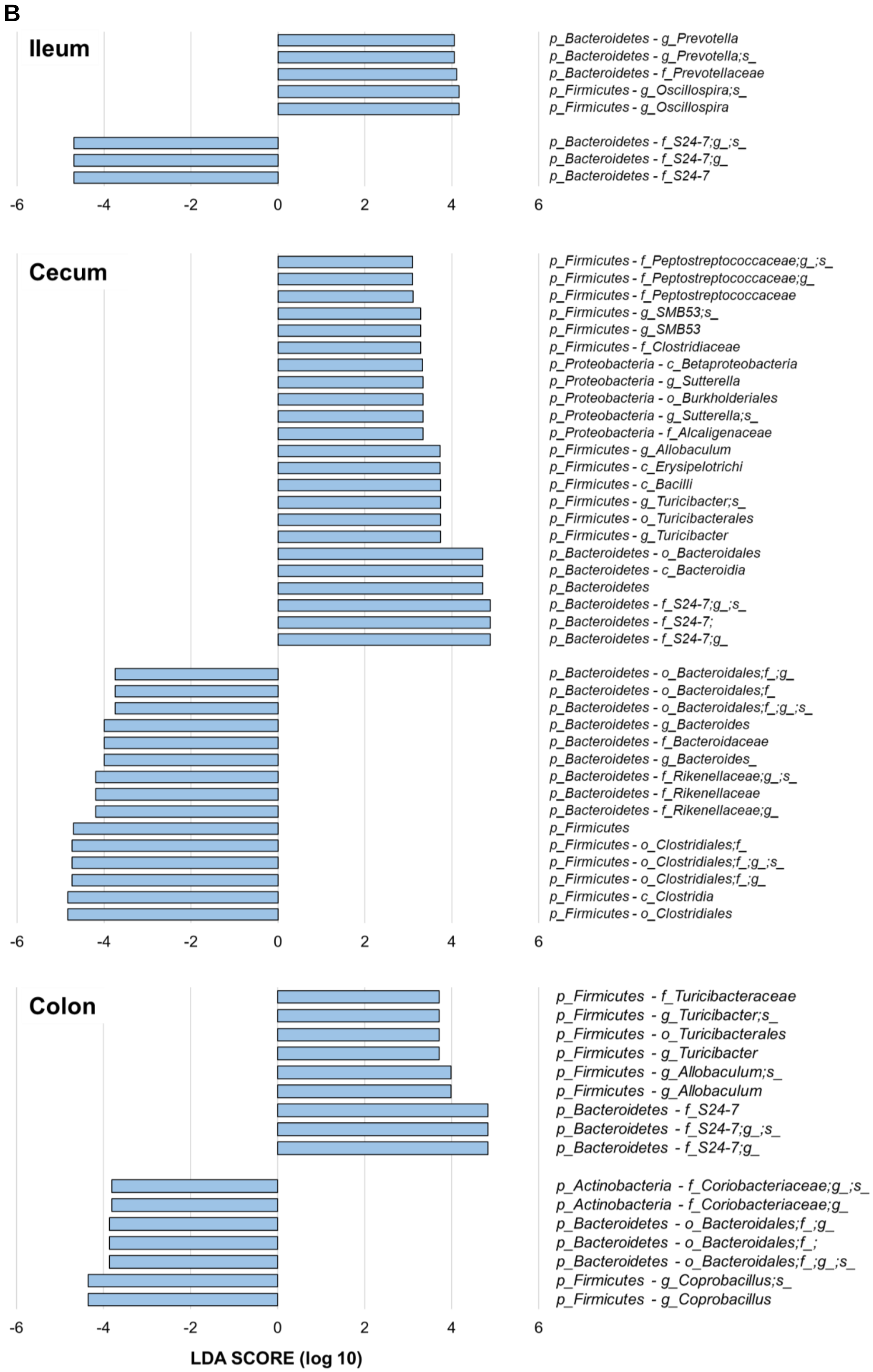
B
Ileum
p_Bacteroidetes - g_Prevotella
p_Bacteroidetes - g_Prevotella;s_
p_Bacteroidetes - f_Prevotellaceae
p_Firmicutes - g_Oscillospira;s_
p_Firmicutes - g_Oscillospira
p_Bacteroidetes - f_S24-7;g_;s_
p_Bacteroidetes - f_S24-7;g_
p_Bacteroidetes - f_S24-7
-6 -4 -2 0 2 4 6
Cecum
p_Firmicutes - f_Peptostreptococcaceae;g_;s_
p_Firmicutes - f_Peptostreptococcaceae;g_
p_Firmicutes - f_Peptostreptococcaceae
p_Firmicutes - g_SMB53;s_
p_Firmicutes - g_SMB53
p_Firmicutes - f_Clostridiaceae
p_Proteobacteria - c_Betaproteobacteria
p_Proteobacteria - g_Sutterella
p_Proteobacteria - o_Burkholderiales
p_Proteobacteria - g_Sutterella;s_
p_Proteobacteria - f_Alcaligenaceae
p_Firmicutes - g_Allobaculum
p_Firmicutes - c_Erysipelotrichi
p_Firmicutes - c_Bacilli
p_Firmicutes - g_Turicibacter;s_
p_Firmicutes - o_Turicibacterales
p_Firmicutes - g_Turicibacter
p_Bacteroidetes - o_Bacteroidales
p_Bacteroidetes - c_Bacteroidia
p_Bacteroidetes
p_Bacteroidetes - f_S24-7;g_;s_
p_Bacteroidetes - f_S24-7;
p_Bacteroidetes - f_S24-7;g_
p_Bacteroidetes - o_Bacteroidales;f_;g_
p_Bacteroidetes - o_Bacteroidales;f_
p_Bacteroidetes - o_Bacteroidales;f_;g_;s_
p_Bacteroidetes - g_Bacteroides
p_Bacteroidetes - f_Bacteroidaceae
p_Bacteroidetes - g_Bacteroides_
p_Bacteroidetes - f_Rikenellaceae;g_;s_
p_Bacteroidetes - f_Rikenellaceae
p_Bacteroidetes - f_Rikenellaceae;g_
p_Firmicutes
p_Firmicutes - o_Clostridiales;f_
p_Firmicutes - o_Clostridiales;f_;g_;s_
p_Firmicutes - o_Clostridiales;f_;g_
p_Firmicutes - c_Clostridia
p_Firmicutes - o_Clostridiales
-6 -4 -2 0 2 4 6
Colon
p_Firmicutes - f_Turicibacteraceae
p_Firmicutes - g_Turicibacter;s_
p_Firmicutes - o_Turicibacterales
p_Firmicutes - g_Turicibacter
p_Firmicutes - g_Allobaculum;s_
p_Firmicutes - g_Allobaculum
p_Bacteroidetes - f_S24-7
p_Bacteroidetes - f_S24-7;g_;s_
p_Bacteroidetes - f_S24-7;g_
p_Actinobacteria - f_Coriobacteriaceae;g_;s_
p_Actinobacteria - f_Coriobacteriaceae;g_
p_Bacteroidetes - o_Bacteroidales;f_;g_
p_Bacteroidetes - o_Bacteroidales;f_;
p_Bacteroidetes - o_Bacteroidales;f_;g_;s_
p_Firmicutes - g_Coprobacillus;s_
p_Firmicutes - g_Coprobacillus
-6 -4 -2 0 2 4 6
LDA SCORE (log 10)

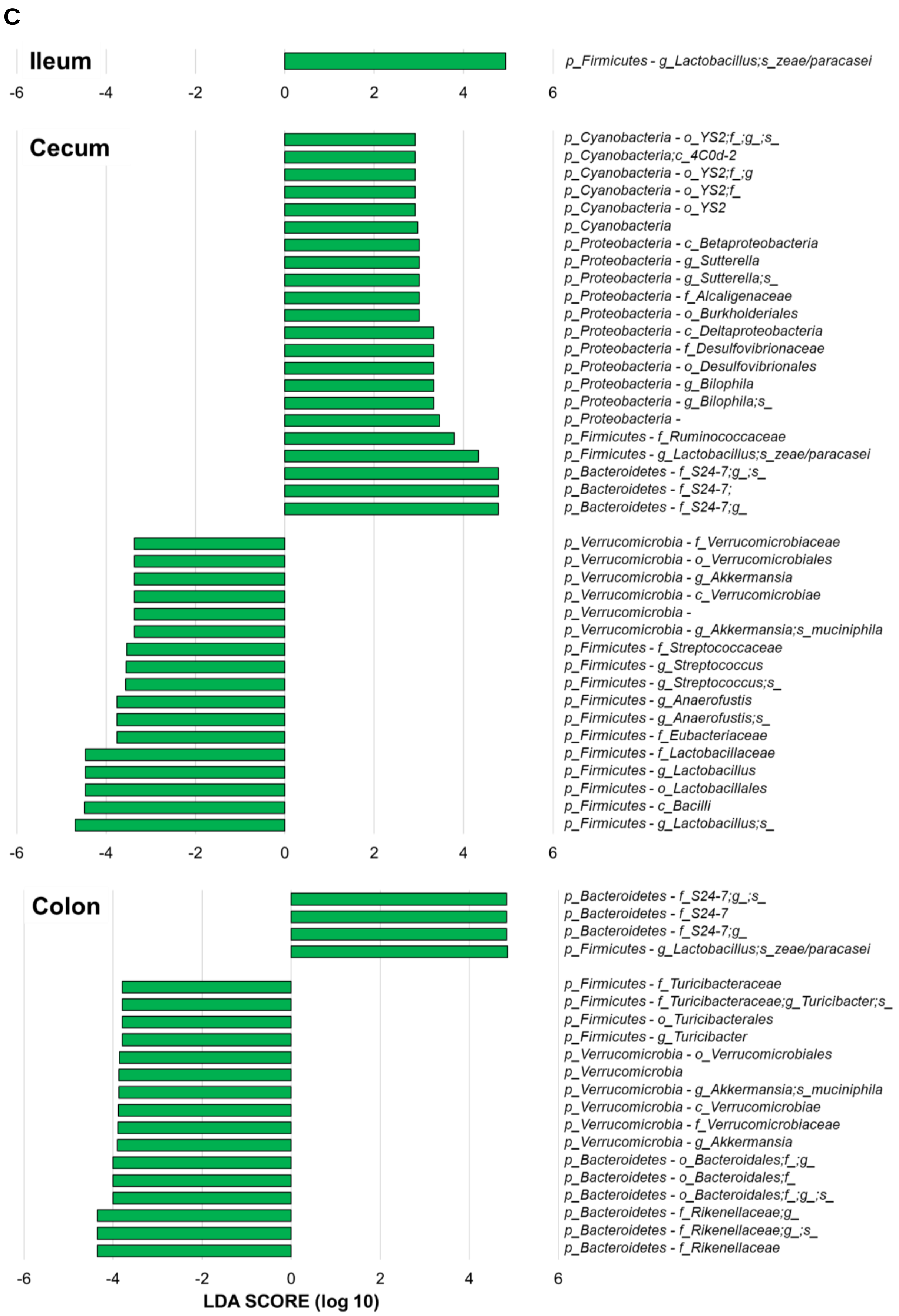
C
Ileum
p_Firmicutes - g_Lactobacillus;s_zeae/paracasei
-6
-4
-2
0
2
4
6
Cecum
p_Cyanobacteria - o_YS2;f_;g_;s_
p_Cyanobacteria;c_4C0d-2
p_Cyanobacteria - o_YS2;f_;g
p_Cyanobacteria - o_YS2;f_
p_Cyanobacteria - o_YS2
p_Cyanobacteria
p_Proteobacteria - c_Betaproteobacteria
p_Proteobacteria - g_Sutterella
p_Proteobacteria - g_Sutterella;s_
p_Proteobacteria - f_Alcaligenaceae
p_Proteobacteria - o_Burkholderiales
p_Proteobacteria - c_Deltaproteobacteria
p_Proteobacteria - f_Desulfovibrionaceae
p_Proteobacteria - o_Desulfovibrionales
p_Proteobacteria - g_Bilophila
p_Proteobacteria - g_Bilophila;s_
p_Proteobacteria -
p_Firmicutes - f_Ruminococcaceae
p_Firmicutes - g_Lactobacillus;s_zeae/paracasei
p_Bacteroidetes - f_S24-7;g_;s_
p_Bacteroidetes - f_S24-7;
p_Bacteroidetes - f_S24-7;g_
p_Verrucomicrobia - f_Verrucomicrobiaceae
p_Verrucomicrobia - o_Verrucomicrobiales
p_Verrucomicrobia - g_Akkermansia
p_Verrucomicrobia - c_Verrucomicrobiae
p_Verrucomicrobia -
p_Verrucomicrobia - g_Akkermansia;s_muciniphila
p_Firmicutes - f_Streptococcaceae
p_Firmicutes - g_Streptococcus
p_Firmicutes - g_Streptococcus;s_
p_Firmicutes - g_Anaerofustis
p_Firmicutes - g_Anaerofustis;s_
p_Firmicutes - f_Eubacteriaceae
p_Firmicutes - f_Lactobacillaceae
p_Firmicutes - g_Lactobacillus
p_Firmicutes - o_Lactobacillales
p_Firmicutes - c_Bacilli
p_Firmicutes - g_Lactobacillus;s_
-6
-4
-2
0
2
4
6
Colon
p_Bacteroidetes - f_S24-7;g_;s_
p_Bacteroidetes - f_S24-7
p_Bacteroidetes - f_S24-7;g_
p_Firmicutes - g_Lactobacillus;s_zeae/paracasei
p_Firmicutes - f_Turicibacteraceae
p_Firmicutes - f_Turicibacteraceae;g_Turicibacter;s_
p_Firmicutes - o_Turicibacterales
p_Firmicutes - g_Turicibacter
p_Verrucomicrobia - o_Verrucomicrobiales
p_Verrucomicrobia
p_Verrucomicrobia - g_Akkermansia;s_muciniphila
p_Verrucomicrobia - c_Verrucomicrobiae
p_Verrucomicrobia - f_Verrucomicrobiaceae
p_Verrucomicrobia - g_Akkermansia
p_Bacteroidetes - o_Bacteroidales;f_;g_
p_Bacteroidetes - o_Bacteroidales;f_
p_Bacteroidetes - o_Bacteroidales;f_;g_;s_
p_Bacteroidetes - f_Rikenellaceae;g_
p_Bacteroidetes - f_Rikenellaceae;g_;s_
p_Bacteroidetes - f_Rikenellaceae
-6
-4
-2
0
2
4
6
LDA SCORE (log 10)

Fig. S4. Operational taxonomic units (OTUs) distinguishing PBS- from probiotic-gavaged mice determined by using the DESeq2 negative binomial distribution method on 16S rRNA gene profiling data. **A**, *B. bifidum* MIMBb23sg. **B**, *L. helveticus* MIMLh5. **C**, *L. paracasei* DG. The colors in the black/yellow heatmap represents the mean of normalized relative abundances of the reported OTUs. The taxonomic lineage of each taxon is shown; p, phylum; c, class; o, order; f, family; g, genus; s, species. Positive fold changes (shown on a red background) indicate OTUs whose relative abundance is significantly higher in bacteria-gavaged mice; negative fold changes (blue background) indicate OTUs whose relative abundance is higher in PBS-gavaged mice.

A

Mice gavaged with *Bifidobacterium bifidum* MIMBb23sg

Ileum

OTU nr.	Taxonomy	Mean normalized abundance PBS	Mean normalized abundance 23sg	log2 Fold Change	padj
**365385**	***p_Actinobacteria;c_Actinobacteria;o_Bifidobacteriales;f_Bifidobacteriaceae;g_Bifidobacterium;s_bifidum* (strain MIMBb23sg)**			**2.32**	**8.33E-03**
168047	*p_Actinobacteria;c_Coriobacteriia;o_Coriobacteriales;f_Coriobacteriaceae;g_Adlercreutzia;s_*			**-3.77**	5.22E-04
276151	*p_Actinobacteria;c_Coriobacteriia;o_Coriobacteriales;f_Coriobacteriaceae;g_Adlercreutzia;s_*			**-2.51**	5.38E-03
631764	*p_Actinobacteria;c_Coriobacteriia;o_Coriobacteriales;f_Coriobacteriaceae;g_Adlercreutzia;s_*			**-2.55**	5.82E-03
315485	*p_Actinobacteria;c_Coriobacteriia;o_Coriobacteriales;f_Coriobacteriaceae;g_Adlercreutzia;s_*			**-2.49**	8.90E-03
193279	*p_Actinobacteria;c_Coriobacteriia;o_Coriobacteriales;f_Coriobacteriaceae;g_Adlercreutzia;s_*			**-2.23**	1.30E-02
275974	*p_Actinobacteria;c_Coriobacteriia;o_Coriobacteriales;f_Coriobacteriaceae;g_Adlercreutzia;s_*			**-2.04**	2.37E-02
339905	*p_Bacteroidetes;c_Bacteroidia;o_Bacteroidales;f_S24-7;g_;s_*			**-6.15**	7.66E-08
348038	*p_Bacteroidetes;c_Bacteroidia;o_Bacteroidales;f_S24-7;g_;s_*			**-4.34**	4.88E-06
356226	*p_Bacteroidetes;c_Bacteroidia;o_Bacteroidales;f_S24-7;g_;s_*			**-4.44**	7.77E-06
801260	*p_Bacteroidetes;c_Bacteroidia;o_Bacteroidales;f_S24-7;g_;s_*			**-4.82**	1.17E-05
430194	*p_Bacteroidetes;c_Bacteroidia;o_Bacteroidales;f_S24-7;g_;s_*			**-4.77**	1.17E-05
277120	*p_Bacteroidetes;c_Bacteroidia;o_Bacteroidales;f_S24-7;g_;s_*			**-4.74**	1.81E-05
233587	*p_Bacteroidetes;c_Bacteroidia;o_Bacteroidales;f_S24-7;g_;s_*			**-4.63**	2.76E-05
416078	*p_Bacteroidetes;c_Bacteroidia;o_Bacteroidales;f_S24-7;g_;s_*			**-4.29**	5.09E-05
188410	*p_Bacteroidetes;c_Bacteroidia;o_Bacteroidales;f_S24-7;g_;s_*			**-4.57**	7.60E-05
423455	*p_Bacteroidetes;c_Bacteroidia;o_Bacteroidales;f_S24-7;g_;s_*			**-4.17**	1.97E-04
320169	*p_Bacteroidetes;c_Bacteroidia;o_Bacteroidales;f_S24-7;g_;s_*			**-3.79**	1.97E-04
263111	*p_Bacteroidetes;c_Bacteroidia;o_Bacteroidales;f_S24-7;g_;s_*			**-3.59**	1.97E-04
175706	*p_Bacteroidetes;c_Bacteroidia;o_Bacteroidales;f_S24-7;g_;s_*			**-4.14**	2.42E-04
331043	*p_Bacteroidetes;c_Bacteroidia;o_Bacteroidales;f_S24-7;g_;s_*			**-3.73**	2.80E-04
338258	*p_Bacteroidetes;c_Bacteroidia;o_Bacteroidales;f_S24-7;g_;s_*			**-3.31**	5.84E-04
228730	*p_Bacteroidetes;c_Bacteroidia;o_Bacteroidales;f_S24-7;g_;s_*			**-3.76**	6.55E-04
261350	*p_Bacteroidetes;c_Bacteroidia;o_Bacteroidales;f_S24-7;g_;s_*			**-3.57**	7.21E-04
191568	*p_Bacteroidetes;c_Bacteroidia;o_Bacteroidales;f_S24-7;g_;s_*			**-3.55**	7.21E-04
196385	*p_Bacteroidetes;c_Bacteroidia;o_Bacteroidales;f_S24-7;g_;s_*			**-3.48**	7.21E-04
184381	*p_Bacteroidetes;c_Bacteroidia;o_Bacteroidales;f_S24-7;g_;s_*			**-3.29**	7.96E-04
353012	*p_Bacteroidetes;c_Bacteroidia;o_Bacteroidales;f_S24-7;g_;s_*			**-3.96**	9.65E-04
264657	*p_Bacteroidetes;c_Bacteroidia;o_Bacteroidales;f_S24-7;g_;s_*			**-3.26**	1.98E-03
263479	*p_Bacteroidetes;c_Bacteroidia;o_Bacteroidales;f_S24-7;g_;s_*			**-3.01**	1.98E-03
196578	*p_Bacteroidetes;c_Bacteroidia;o_Bacteroidales;f_S24-7;g_;s_*			**-2.99**	2.89E-03
353453	*p_Bacteroidetes;c_Bacteroidia;o_Bacteroidales;f_S24-7;g_;s_*			**-3.03**	3.43E-03
345126	*p_Bacteroidetes;c_Bacteroidia;o_Bacteroidales;f_S24-7;g_;s_*			**-2.81**	4.90E-03
389282	*p_Bacteroidetes;c_Bacteroidia;o_Bacteroidales;f_S24-7;g_;s_*			**-2.69**	5.36E-03
334040	*p_Bacteroidetes;c_Bacteroidia;o_Bacteroidales;f_S24-7;g_;s_*			**-2.62**	5.44E-03
3231096	*p_Bacteroidetes;c_Bacteroidia;o_Bacteroidales;f_S24-7;g_;s_*			**-2.64**	7.02E-03
342962	*p_Bacteroidetes;c_Bacteroidia;o_Bacteroidales;f_S24-7;g_;s_*			**-2.72**	8.15E-03
258485	*p_Bacteroidetes;c_Bacteroidia;o_Bacteroidales;f_S24-7;g_;s_*			**-2.59**	8.20E-03
266860	*p_Bacteroidetes;c_Bacteroidia;o_Bacteroidales;f_S24-7;g_;s_*			**-2.37**	8.20E-03
192494	*p_Bacteroidetes;c_Bacteroidia;o_Bacteroidales;f_S24-7;g_;s_*			**-2.59**	9.67E-03
188427	*p_Bacteroidetes;c_Bacteroidia;o_Bacteroidales;f_S24-7;g_;s_*			**-2.14**	1.45E-02
264734	*p_Bacteroidetes;c_Bacteroidia;o_Bacteroidales;f_S24-7;g_;s_*			**-2.07**	2.14E-02
182849	*p_Bacteroidetes;c_Bacteroidia;o_Bacteroidales;f_S24-7;g_;s_*			**-2.04**	2.22E-02
313499	*p_Bacteroidetes;c_Bacteroidia;o_Bacteroidales;f_S24-7;g_;s_*			**-1.89**	2.68E-02
331772	*p_Bacteroidetes;c_Bacteroidia;o_Bacteroidales;f_S24-7;g_;s_*			**-1.97**	4.09E-02
372368	*p_Bacteroidetes;c_Bacteroidia;o_Bacteroidales;f_S24-7;g_;s_*			**-1.98**	4.77E-02
463794	*p_Firmicutes;c_Bacilli;o_Lactobacillales;f_Lactobacillaceae;g_Lactobacillus;s_*			**-3.06**	1.25E-04
182758	*p_Firmicutes;c_Bacilli;o_Lactobacillales;f_Lactobacillaceae;g_Lactobacillus;s_*			**-2.83**	6.97E-04
452823	*p_Firmicutes;c_Bacilli;o_Lactobacillales;f_Lactobacillaceae;g_Lactobacillus;s_*			**-2.82**	1.20E-03
333178	*p_Firmicutes;c_Bacilli;o_Lactobacillales;f_Lactobacillaceae;g_Lactobacillus;s_*			**-2.64**	1.98E-03
242917	*p_Firmicutes;c_Bacilli;o_Lactobacillales;f_Lactobacillaceae;g_Lactobacillus;s_*			**-2.71**	4.06E-03
588197	*p_Firmicutes;c_Bacilli;o_Lactobacillales;f_Lactobacillaceae;g_Lactobacillus;s_*			**-2.98**	4.46E-03
329402	*p_Firmicutes;c_Bacilli;o_Lactobacillales;f_Lactobacillaceae;g_Lactobacillus;s_*			**-2.66**	5.61E-03

259111	*p_Firmicutes;c_Bacilli;o_Lactobacillales;f_Lactobacillaceae;g_Lactobacillus;s_*			**-2.18**	1.04E-02
851794	*p_Firmicutes;c_Bacilli;o_Lactobacillales;f_Lactobacillaceae;g_Lactobacillus;s_*			**-2.38**	1.33E-02
198641	*p_Firmicutes;c_Bacilli;o_Lactobacillales;f_Lactobacillaceae;g_Lactobacillus;s_*			**-2.46**	1.48E-02
259372	*p_Firmicutes;c_Bacilli;o_Lactobacillales;f_Lactobacillaceae;g_Lactobacillus;s_*			**-2.13**	1.67E-02
322250	*p_Firmicutes;c_Bacilli;o_Lactobacillales;f_Lactobacillaceae;g_Lactobacillus;s_*			**-2.21**	2.20E-02
164664	*p_Firmicutes;c_Bacilli;o_Lactobacillales;f_Lactobacillaceae;g_Lactobacillus;s_*			**-1.91**	3.46E-02
3946926	*p_Firmicutes;c_Bacilli;o_Lactobacillales;f_Lactobacillaceae;g_Lactobacillus;s_*			**-1.88**	3.57E-02
350242	*p_Firmicutes;c_Bacilli;o_Lactobacillales;f_Lactobacillaceae;g_Lactobacillus;s_*			**-1.79**	3.63E-02
582884	*p_Firmicutes;c_Bacilli;o_Lactobacillales;f_Lactobacillaceae;g_Lactobacillus;s_*			**-1.85**	4.41E-02
342787	*p_Firmicutes;c_Bacilli;o_Lactobacillales;f_Lactobacillaceae;g_Lactobacillus;s_*			**-1.73**	4.43E-02
187233	*p_Firmicutes;c_Bacilli;o_Lactobacillales;f_Lactobacillaceae;g_Lactobacillus;s_*			**-1.66**	4.43E-02
411486	*p_Firmicutes;c_Bacilli;o_Lactobacillales;f_Lactobacillaceae;g_Lactobacillus;s_reuteri*			**-3.22**	2.29E-03
433676	*p_Firmicutes;c_Bacilli;o_Lactobacillales;f_Lactobacillaceae;g_Lactobacillus;s_reuteri*			**-2.92**	7.42E-03
356144	*p_Firmicutes;c_Bacilli;o_Lactobacillales;f_Lactobacillaceae;g_Lactobacillus;s_reuteri*			**-2.82**	8.90E-03
343431	*p_Firmicutes;c_Bacilli;o_Lactobacillales;f_Lactobacillaceae;g_Lactobacillus;s_reuteri*			**-2.13**	1.99E-02
348336	*p_Firmicutes;c_Clostridia;o_Clostridiales;f_;g_;s_*			**-4.16**	1.93E-04
342786	*p_Firmicutes;c_Clostridia;o_Clostridiales;f_;g_;s_*			**-3.07**	3.57E-03
4402077	*p_Firmicutes;c_Clostridia;o_Clostridiales;f_;g_;s_*			**-2.71**	5.36E-03
169845	*p_Firmicutes;c_Clostridia;o_Clostridiales;f_;g_;s_*			**-2.65**	8.15E-03
323526	*p_Firmicutes;c_Clostridia;o_Clostridiales;f_Clostridiaceae;g_;s_*			**-4.72**	5.09E-05
396697	*p_Firmicutes;c_Clostridia;o_Clostridiales;f_Clostridiaceae;g_Clostridium;s_perfringens*			**-3.98**	3.65E-04
555945	*p_Firmicutes;c_Clostridia;o_Clostridiales;f_Clostridiaceae;g_SMB53;s_*			**-3.01**	3.12E-03
340189	*p_Firmicutes;c_Clostridia;o_Clostridiales;f_Lachnospiraceae;g_;s_*			**-3.70**	7.45E-04
330116	*p_Firmicutes;c_Clostridia;o_Clostridiales;f_Lachnospiraceae;g_[Ruminococcus];s_gnavus*			**-5.20**	3.12E-05
383971	*p_Firmicutes;c_Clostridia;o_Clostridiales;f_Lachnospiraceae;g_[Ruminococcus];s_gnavus*			**-4.13**	1.83E-04
295075	*p_Firmicutes;c_Clostridia;o_Clostridiales;f_Lachnospiraceae;g_[Ruminococcus];s_gnavus*			**-3.40**	1.07E-03
278931	*p_Firmicutes;c_Clostridia;o_Clostridiales;f_Lachnospiraceae;g_Dorea;s_*			**-2.48**	1.08E-02
276478	*p_Firmicutes;c_Clostridia;o_Clostridiales;f_Peptostreptococcaceae;g_;s_*			**-3.06**	3.82E-03
340794	*p_Firmicutes;c_Clostridia;o_Clostridiales;f_Ruminococcaceae;g_Ruminococcus;s_*			**3.32**	1.88E-03
797229	*p_Proteobacteria;c_Gammaproteobacteria;o_Enterobacteriales;f_Enterobacteriaceae;g_;s_*			**2.84**	1.75E-03
768553	*p_Proteobacteria;c_Gammaproteobacteria;o_Enterobacteriales;f_Enterobacteriaceae;g_Proteus;s_*			**-3.85**	7.21E-04
4440970	*p_TM7;c_TM7-3;o_CW040;f_F16;g_;s_*			**-2.27**	1.38E-02

Cecum

**OTU nr.**	**Taxonomy**	**Mean normalized abundance**		**log2 Fold Change**	**padj**
		**PBS**	**23sg**		
365385	***p_Actinobacteria;c_Actinobacteria;o_Bifidobacteriales;f_Bifidobacteriaceae;g_Bifidobacterium;s_bifidum* (strain MIMBb23sg)**			**9.60**	**2.22E-41**
275974	*p_Actinobacteria;c_Coriobacteriia;o_Coriobacteriales;f_Coriobacteriaceae;g_Adlercreutzia;s_*			**1.55**	4.72E-02
3013444	*p_Bacteroidetes;c_Bacteroidia;o_Bacteroidales;f_;g_;s_*			**-1.93**	1.70E-02
270391	*p_Bacteroidetes;c_Bacteroidia;o_Bacteroidales;f_Rikenellaceae;g_;s_*			**-1.87**	2.47E-04
355746	*p_Bacteroidetes;c_Bacteroidia;o_Bacteroidales;f_S24-7;g_;s_*			**5.32**	1.45E-30
331772	*p_Bacteroidetes;c_Bacteroidia;o_Bacteroidales;f_S24-7;g_;s_*			**4.89**	2.05E-18
421792	*p_Bacteroidetes;c_Bacteroidia;o_Bacteroidales;f_S24-7;g_;s_*			**5.51**	1.83E-17
215214	*p_Bacteroidetes;c_Bacteroidia;o_Bacteroidales;f_S24-7;g_;s_*			**5.43**	3.33E-15
339549	*p_Bacteroidetes;c_Bacteroidia;o_Bacteroidales;f_S24-7;g_;s_*			**4.32**	5.74E-15
206790	*p_Bacteroidetes;c_Bacteroidia;o_Bacteroidales;f_S24-7;g_;s_*			**4.98**	1.49E-13
276218	*p_Bacteroidetes;c_Bacteroidia;o_Bacteroidales;f_S24-7;g_;s_*			**5.69**	1.49E-13
210665	*p_Bacteroidetes;c_Bacteroidia;o_Bacteroidales;f_S24-7;g_;s_*			**4.58**	9.42E-13
264298	*p_Bacteroidetes;c_Bacteroidia;o_Bacteroidales;f_S24-7;g_;s_*			**5.07**	9.85E-13
217100	*p_Bacteroidetes;c_Bacteroidia;o_Bacteroidales;f_S24-7;g_;s_*			**4.80**	1.39E-12
195919	*p_Bacteroidetes;c_Bacteroidia;o_Bacteroidales;f_S24-7;g_;s_*			**4.68**	1.43E-12
215495	*p_Bacteroidetes;c_Bacteroidia;o_Bacteroidales;f_S24-7;g_;s_*			**4.21**	2.10E-12
330772	*p_Bacteroidetes;c_Bacteroidia;o_Bacteroidales;f_S24-7;g_;s_*			**3.76**	1.80E-11
275339	*p_Bacteroidetes;c_Bacteroidia;o_Bacteroidales;f_S24-7;g_;s_*			**3.65**	2.70E-10
341913	*p_Bacteroidetes;c_Bacteroidia;o_Bacteroidales;f_S24-7;g_;s_*			**4.08**	7.98E-10
2212505	*p_Bacteroidetes;c_Bacteroidia;o_Bacteroidales;f_S24-7;g_;s_*			**3.94**	5.52E-09
191789	*p_Bacteroidetes;c_Bacteroidia;o_Bacteroidales;f_S24-7;g_;s_*			**4.27**	1.31E-08
189730	*p_Bacteroidetes;c_Bacteroidia;o_Bacteroidales;f_S24-7;g_;s_*			**4.22**	2.63E-08
343853	*p_Bacteroidetes;c_Bacteroidia;o_Bacteroidales;f_S24-7;g_;s_*			**3.74**	3.32E-08
233435	*p_Bacteroidetes;c_Bacteroidia;o_Bacteroidales;f_S24-7;g_;s_*			**3.65**	3.40E-08
389282	*p_Bacteroidetes;c_Bacteroidia;o_Bacteroidales;f_S24-7;g_;s_*			**3.19**	5.84E-08
203605	*p_Bacteroidetes;c_Bacteroidia;o_Bacteroidales;f_S24-7;g_;s_*			**3.85**	3.94E-07
198644	*p_Bacteroidetes;c_Bacteroidia;o_Bacteroidales;f_S24-7;g_;s_*			**3.90**	3.99E-07
174587	*p_Bacteroidetes;c_Bacteroidia;o_Bacteroidales;f_S24-7;g_;s_*			**3.70**	5.14E-07
346870	*p_Bacteroidetes;c_Bacteroidia;o_Bacteroidales;f_S24-7;g_;s_*			**3.84**	8.52E-07
247715	*p_Bacteroidetes;c_Bacteroidia;o_Bacteroidales;f_S24-7;g_;s_*			**3.45**	1.47E-06
194048	*p_Bacteroidetes;c_Bacteroidia;o_Bacteroidales;f_S24-7;g_;s_*			**3.80**	4.07E-06
210383	*p_Bacteroidetes;c_Bacteroidia;o_Bacteroidales;f_S24-7;g_;s_*			**3.39**	1.37E-05
379505	*p_Bacteroidetes;c_Bacteroidia;o_Bacteroidales;f_S24-7;g_;s_*			**3.62**	1.67E-05
416078	*p_Bacteroidetes;c_Bacteroidia;o_Bacteroidales;f_S24-7;g_;s_*			**2.43**	7.71E-05
264734	*p_Bacteroidetes;c_Bacteroidia;o_Bacteroidales;f_S24-7;g_;s_*			**3.00**	7.71E-05
204171	*p_Bacteroidetes;c_Bacteroidia;o_Bacteroidales;f_S24-7;g_;s_*			**3.19**	1.47E-04
270158	*p_Bacteroidetes;c_Bacteroidia;o_Bacteroidales;f_S24-7;g_;s_*			**3.32**	2.17E-04
191749	*p_Bacteroidetes;c_Bacteroidia;o_Bacteroidales;f_S24-7;g_;s_*			**2.97**	2.47E-04
208409	*p_Bacteroidetes;c_Bacteroidia;o_Bacteroidales;f_S24-7;g_;s_*			**3.37**	2.47E-04
175080	*p_Bacteroidetes;c_Bacteroidia;o_Bacteroidales;f_S24-7;g_;s_*			**3.01**	2.87E-04
209408	*p_Bacteroidetes;c_Bacteroidia;o_Bacteroidales;f_S24-7;g_;s_*			**3.00**	2.93E-04
215897	*p_Bacteroidetes;c_Bacteroidia;o_Bacteroidales;f_S24-7;g_;s_*			**3.00**	5.31E-04
348088	*p_Bacteroidetes;c_Bacteroidia;o_Bacteroidales;f_S24-7;g_;s_*			**1.80**	2.16E-03
304408	*p_Bacteroidetes;c_Bacteroidia;o_Bacteroidales;f_S24-7;g_;s_*			**2.68**	2.16E-03
192494	*p_Bacteroidetes;c_Bacteroidia;o_Bacteroidales;f_S24-7;g_;s_*			**1.97**	3.25E-03
204088	*p_Bacteroidetes;c_Bacteroidia;o_Bacteroidales;f_S24-7;g_;s_*			**2.48**	3.96E-03

177269	p_Bacteroidetes;c_Bacteroidia;o_Bacteroidales;f_S24-7;g_;s_	**2.46**	4.16E-03
320169	p_Bacteroidetes;c_Bacteroidia;o_Bacteroidales;f_S24-7;g_;s_	**1.77**	4.21E-03
372368	p_Bacteroidetes;c_Bacteroidia;o_Bacteroidales;f_S24-7;g_;s_	**1.78**	7.99E-03
338258	p_Bacteroidetes;c_Bacteroidia;o_Bacteroidales;f_S24-7;g_;s_	**1.80**	8.49E-03
316629	p_Bacteroidetes;c_Bacteroidia;o_Bacteroidales;f_S24-7;g_;s_	**2.20**	8.49E-03
205981	p_Bacteroidetes;c_Bacteroidia;o_Bacteroidales;f_S24-7;g_;s_	**2.30**	8.49E-03
326095	p_Bacteroidetes;c_Bacteroidia;o_Bacteroidales;f_S24-7;g_;s_	**2.74**	8.49E-03
216495	p_Bacteroidetes;c_Bacteroidia;o_Bacteroidales;f_S24-7;g_;s_	**1.93**	9.19E-03
206817	p_Bacteroidetes;c_Bacteroidia;o_Bacteroidales;f_S24-7;g_;s_	**2.23**	1.07E-02
174754	p_Bacteroidetes;c_Bacteroidia;o_Bacteroidales;f_S24-7;g_;s_	**2.15**	1.20E-02
211494	p_Bacteroidetes;c_Bacteroidia;o_Bacteroidales;f_S24-7;g_;s_	**1.72**	1.89E-02
269726	p_Bacteroidetes;c_Bacteroidia;o_Bacteroidales;f_S24-7;g_;s_	**1.79**	1.99E-02
352789	p_Bacteroidetes;c_Bacteroidia;o_Bacteroidales;f_S24-7;g_;s_	**1.86**	1.99E-02
801260	p_Bacteroidetes;c_Bacteroidia;o_Bacteroidales;f_S24-7;g_;s_	**1.99**	2.85E-02
175458	p_Bacteroidetes;c_Bacteroidia;o_Bacteroidales;f_S24-7;g_;s_	**1.40**	4.42E-02
214919	p_Firmicutes;c_Bacilli;o_Turicibacterales;f_Turicibacteraceae;g_Turicibacter;s_	**2.34**	2.87E-02
353657	p_Firmicutes;c_Clostridia;o_Clostridiales;f_;g_;s_	**3.38**	9.33E-04
185334	p_Firmicutes;c_Clostridia;o_Clostridiales;f_;g_;s_	**3.13**	3.11E-03
348398	p_Firmicutes;c_Clostridia;o_Clostridiales;f_;g_;s_	**2.83**	4.16E-03
276312	p_Firmicutes;c_Clostridia;o_Clostridiales;f_;g_;s_	**3.17**	6.02E-03
831409	p_Firmicutes;c_Clostridia;o_Clostridiales;f_;g_;s_	**2.57**	7.46E-03
329712	p_Firmicutes;c_Clostridia;o_Clostridiales;f_;g_;s_	**2.81**	7.67E-03
351593	p_Firmicutes;c_Clostridia;o_Clostridiales;f_;g_;s_	**2.86**	9.19E-03
432444	p_Firmicutes;c_Clostridia;o_Clostridiales;f_;g_;s_	**2.35**	1.28E-02
390820	p_Firmicutes;c_Clostridia;o_Clostridiales;f_;g_;s_	**2.55**	1.34E-02
188697	p_Firmicutes;c_Clostridia;o_Clostridiales;f_;g_;s_	**2.40**	1.89E-02
298723	p_Firmicutes;c_Clostridia;o_Clostridiales;f_;g_;s_	**2.49**	1.97E-02
272516	p_Firmicutes;c_Clostridia;o_Clostridiales;f_;g_;s_	**2.09**	1.99E-02
675863	p_Firmicutes;c_Clostridia;o_Clostridiales;f_;g_;s_	**2.61**	2.87E-02
180671	p_Firmicutes;c_Clostridia;o_Clostridiales;f_;g_;s_	**2.32**	3.24E-02
229562	p_Firmicutes;c_Clostridia;o_Clostridiales;f_;g_;s_	**2.23**	3.29E-02
352049	p_Firmicutes;c_Clostridia;o_Clostridiales;f_;g_;s_	**2.57**	3.60E-02
176172	p_Firmicutes;c_Clostridia;o_Clostridiales;f_;g_;s_	**1.85**	3.73E-02
184584	p_Firmicutes;c_Clostridia;o_Clostridiales;f_;g_;s_	**1.80**	4.61E-02
4365109	p_Firmicutes;c_Clostridia;o_Clostridiales;f_;g_;s_	**-3.70**	3.73E-03
199532	p_Firmicutes;c_Clostridia;o_Clostridiales;f_;g_;s_	**-2.76**	9.61E-03
178738	p_Firmicutes;c_Clostridia;o_Clostridiales;f_Clostridiaceae;g_;s_	**1.89**	4.61E-02
555945	p_Firmicutes;c_Clostridia;o_Clostridiales;f_Clostridiaceae;g_SMB53;s_	**2.63**	8.86E-03
514988	p_Firmicutes;c_Clostridia;o_Clostridiales;f_Clostridiaceae;g_SMB53;s_	**2.22**	1.40E-02
345709	p_Firmicutes;c_Clostridia;o_Clostridiales;f_Lachnospiraceae;g_;s_	**2.45**	1.57E-02
260752	p_Firmicutes;c_Clostridia;o_Clostridiales;f_Lachnospiraceae;g_;s_	**1.88**	3.99E-02
2120784	p_Firmicutes;c_Clostridia;o_Clostridiales;f_Lachnospiraceae;g_;s_	**-2.98**	2.65E-03
194858	p_Firmicutes;c_Clostridia;o_Clostridiales;f_Lachnospiraceae;g_[Ruminococcus];s_gnavus	**2.23**	8.03E-03
267298	p_Firmicutes;c_Clostridia;o_Clostridiales;f_Lachnospiraceae;g_[Ruminococcus];s_gnavus	**2.33**	1.57E-02
266726	p_Firmicutes;c_Clostridia;o_Clostridiales;f_Lachnospiraceae;g_Coprococcus;s_	**2.52**	5.33E-03
339791	p_Firmicutes;c_Clostridia;o_Clostridiales;f_Lachnospiraceae;g_Coprococcus;s_	**-2.60**	6.37E-03
232283	p_Firmicutes;c_Clostridia;o_Clostridiales;f_Lachnospiraceae;g_Coprococcus;s_	**-1.97**	2.28E-02
276478	p_Firmicutes;c_Clostridia;o_Clostridiales;f_Peptostreptococcaceae;g_;s_	**2.54**	1.36E-02
4439489	p_Firmicutes;c_Clostridia;o_Clostridiales;f_Ruminococcaceae;g_;s_	**2.41**	6.24E-04
837859	p_Firmicutes;c_Clostridia;o_Clostridiales;f_Ruminococcaceae;g_;s_	**2.74**	4.07E-03
262258	p_Firmicutes;c_Clostridia;o_Clostridiales;f_Ruminococcaceae;g_;s_	**2.63**	7.46E-03
583134	p_Firmicutes;c_Clostridia;o_Clostridiales;f_Ruminococcaceae;g_;s_	**2.23**	1.46E-02
4338227	p_Firmicutes;c_Clostridia;o_Clostridiales;f_Ruminococcaceae;g_;s_	**2.05**	1.99E-02
171396	p_Firmicutes;c_Clostridia;o_Clostridiales;f_Ruminococcaceae;g_;s_	**2.06**	3.02E-02
443620	p_Firmicutes;c_Clostridia;o_Clostridiales;f_Ruminococcaceae;g_Oscillospira;s_	**2.70**	2.93E-04
1569569	p_Firmicutes;c_Clostridia;o_Clostridiales;f_Ruminococcaceae;g_Oscillospira;s_	**2.30**	1.57E-02
270303	p_Firmicutes;c_Clostridia;o_Clostridiales;f_Ruminococcaceae;g_Oscillospira;s_	**1.66**	3.24E-02
323403	p_Firmicutes;c_Clostridia;o_Clostridiales;f_Ruminococcaceae;g_Oscillospira;s_	**-3.14**	3.34E-03
327900	p_Firmicutes;c_Clostridia;o_Clostridiales;f_Ruminococcaceae;g_Ruminococcus;s_	**2.23**	3.29E-02
262101	p_Firmicutes;c_Erysipelotrichi;o_Erysipelotrichales;f_Erysipelotrichaceae;g_Allobaculum;s_	**4.29**	5.59E-06
437137	p_Proteobacteria;c_Betaproteobacteria;o_Burkholderiales;f_Alcaligenaceae;g_Sutterella;s_	**2.15**	5.41E-03
593043	p_Verrucomicrobia;c_Verrucomicrobiae;o_Verrucomicrobiales;f_Verrucomicrobiaceae;g_Akkermansia;s_muciniphila	**2.51**	1.59E-02
363731	p_Verrucomicrobia;c_Verrucomicrobiae;o_Verrucomicrobiales;f_Verrucomicrobiaceae;g_Akkermansia;s_muciniphila	**2.61**	2.64E-02

Colon

OTU nr.	Taxonomy	Mean normalized abundance PBS	Mean normalized abundance 23sg	log2 Fold Change	padj
**365385**	**p_Actinobacteria;c_Actinobacteria;o_Bifidobacteriales;f_Bifidobacteriaceae;g_Bifidobacterium;s_bifidum (strain MIMBb23sg)**			**10.02**	5.39E-47
3013444	p_Bacteroidetes;c_Bacteroidia;o_Bacteroidales;f_;g_;s_			**-2.32**	9.15E-04
421792	p_Bacteroidetes;c_Bacteroidia;o_Bacteroidales;f_S24-7;g_;s_			**5.31**	3.22E-14
331772	p_Bacteroidetes;c_Bacteroidia;o_Bacteroidales;f_S24-7;g_;s_			**5.25**	8.25E-14
215214	p_Bacteroidetes;c_Bacteroidia;o_Bacteroidales;f_S24-7;g_;s_			**5.36**	9.07E-13
276218	p_Bacteroidetes;c_Bacteroidia;o_Bacteroidales;f_S24-7;g_;s_			**5.55**	1.13E-11
339549	p_Bacteroidetes;c_Bacteroidia;o_Bacteroidales;f_S24-7;g_;s_			**4.35**	1.70E-11
355746	p_Bacteroidetes;c_Bacteroidia;o_Bacteroidales;f_S24-7;g_;s_			**4.38**	1.21E-10
210665	p_Bacteroidetes;c_Bacteroidia;o_Bacteroidales;f_S24-7;g_;s_			**4.60**	1.54E-10
233435	p_Bacteroidetes;c_Bacteroidia;o_Bacteroidales;f_S24-7;g_;s_			**3.85**	2.03E-10
343853	p_Bacteroidetes;c_Bacteroidia;o_Bacteroidales;f_S24-7;g_;s_			**3.91**	5.95E-10
2212505	p_Bacteroidetes;c_Bacteroidia;o_Bacteroidales;f_S24-7;g_;s_			**4.39**	3.27E-09

217100	*p_Bacteroidetes;c_Bacteroidia;o_Bacteroidales;f_S24-7;g_;s_*	**4.72**	5.81E-09
330772	*p_Bacteroidetes;c_Bacteroidia;o_Bacteroidales;f_S24-7;g_;s_*	**3.72**	7.25E-09
341913	*p_Bacteroidetes;c_Bacteroidia;o_Bacteroidales;f_S24-7;g_;s_*	**3.72**	4.47E-08
215495	*p_Bacteroidetes;c_Bacteroidia;o_Bacteroidales;f_S24-7;g_;s_*	**4.21**	6.35E-08
206790	*p_Bacteroidetes;c_Bacteroidia;o_Bacteroidales;f_S24-7;g_;s_*	**4.72**	6.35E-08
275339	*p_Bacteroidetes;c_Bacteroidia;o_Bacteroidales;f_S24-7;g_;s_*	**3.65**	1.88E-07
346870	*p_Bacteroidetes;c_Bacteroidia;o_Bacteroidales;f_S24-7;g_;s_*	**4.00**	2.38E-07
264298	*p_Bacteroidetes;c_Bacteroidia;o_Bacteroidales;f_S24-7;g_;s_*	**4.26**	4.50E-07
326095	*p_Bacteroidetes;c_Bacteroidia;o_Bacteroidales;f_S24-7;g_;s_*	**3.59**	2.05E-06
195919	*p_Bacteroidetes;c_Bacteroidia;o_Bacteroidales;f_S24-7;g_;s_*	**3.79**	2.05E-06
174587	*p_Bacteroidetes;c_Bacteroidia;o_Bacteroidales;f_S24-7;g_;s_*	**4.08**	2.05E-06
264734	*p_Bacteroidetes;c_Bacteroidia;o_Bacteroidales;f_S24-7;g_;s_*	**2.83**	7.61E-06
189730	*p_Bacteroidetes;c_Bacteroidia;o_Bacteroidales;f_S24-7;g_;s_*	**3.53**	1.80E-05
191789	*p_Bacteroidetes;c_Bacteroidia;o_Bacteroidales;f_S24-7;g_;s_*	**3.59**	2.55E-05
389282	*p_Bacteroidetes;c_Bacteroidia;o_Bacteroidales;f_S24-7;g_;s_*	**2.76**	7.62E-05
247715	*p_Bacteroidetes;c_Bacteroidia;o_Bacteroidales;f_S24-7;g_;s_*	**3.06**	8.91E-05
203605	*p_Bacteroidetes;c_Bacteroidia;o_Bacteroidales;f_S24-7;g_;s_*	**3.69**	1.58E-04
198644	*p_Bacteroidetes;c_Bacteroidia;o_Bacteroidales;f_S24-7;g_;s_*	**3.68**	2.47E-04
194048	*p_Bacteroidetes;c_Bacteroidia;o_Bacteroidales;f_S24-7;g_;s_*	**3.31**	5.51E-04
379505	*p_Bacteroidetes;c_Bacteroidia;o_Bacteroidales;f_S24-7;g_;s_*	**3.05**	9.20E-04
210383	*p_Bacteroidetes;c_Bacteroidia;o_Bacteroidales;f_S24-7;g_;s_*	**2.91**	9.30E-04
209408	*p_Bacteroidetes;c_Bacteroidia;o_Bacteroidales;f_S24-7;g_;s_*	**2.78**	1.82E-03
316629	*p_Bacteroidetes;c_Bacteroidia;o_Bacteroidales;f_S24-7;g_;s_*	**2.83**	1.96E-03
352789	*p_Bacteroidetes;c_Bacteroidia;o_Bacteroidales;f_S24-7;g_;s_*	**2.20**	3.36E-03
215897	*p_Bacteroidetes;c_Bacteroidia;o_Bacteroidales;f_S24-7;g_;s_*	**2.61**	3.36E-03
270158	*p_Bacteroidetes;c_Bacteroidia;o_Bacteroidales;f_S24-7;g_;s_*	**3.06**	6.72E-03
177269	*p_Bacteroidetes;c_Bacteroidia;o_Bacteroidales;f_S24-7;g_;s_*	**2.51**	7.76E-03
269726	*p_Bacteroidetes;c_Bacteroidia;o_Bacteroidales;f_S24-7;g_;s_*	**2.31**	8.91E-03
189778	*p_Bacteroidetes;c_Bacteroidia;o_Bacteroidales;f_S24-7;g_;s_*	**2.50**	8.97E-03
191749	*p_Bacteroidetes;c_Bacteroidia;o_Bacteroidales;f_S24-7;g_;s_*	**2.64**	1.21E-02
204171	*p_Bacteroidetes;c_Bacteroidia;o_Bacteroidales;f_S24-7;g_;s_*	**2.32**	1.84E-02
416078	*p_Bacteroidetes;c_Bacteroidia;o_Bacteroidales;f_S24-7;g_;s_*	**1.91**	2.21E-02
338258	*p_Bacteroidetes;c_Bacteroidia;o_Bacteroidales;f_S24-7;g_;s_*	**2.00**	2.77E-02
304408	*p_Bacteroidetes;c_Bacteroidia;o_Bacteroidales;f_S24-7;g_;s_*	**2.11**	3.10E-02
216495	*p_Bacteroidetes;c_Bacteroidia;o_Bacteroidales;f_S24-7;g_;s_*	**1.92**	3.16E-02
204088	*p_Bacteroidetes;c_Bacteroidia;o_Bacteroidales;f_S24-7;g_;s_*	**2.02**	3.16E-02
206817	*p_Bacteroidetes;c_Bacteroidia;o_Bacteroidales;f_S24-7;g_;s_*	**2.09**	3.30E-02
349175	*p_Bacteroidetes;c_Bacteroidia;o_Bacteroidales;f_S24-7;g_;s_*	**1.69**	3.79E-02
206456	*p_Bacteroidetes;c_Bacteroidia;o_Bacteroidales;f_S24-7;g_;s_*	**1.70**	4.11E-02
175706	*p_Bacteroidetes;c_Bacteroidia;o_Bacteroidales;f_S24-7;g_;s_*	**-1.97**	8.17E-03
318320	*p_Firmicutes;c_Bacilli;o_Lactobacillales;f_Lactobacillaceae;g_Lactobacillus;s_*	**1.68**	3.01E-02
277512	*p_Firmicutes;c_Clostridia;o_Clostridiales;f_;g_;s_*	**2.85**	1.01E-02
264489	*p_Firmicutes;c_Clostridia;o_Clostridiales;f_;g_;s_*	**2.14**	3.16E-02
4365109	*p_Firmicutes;c_Clostridia;o_Clostridiales;f_;g_;s_*	**-3.31**	2.17E-03
4402077	*p_Firmicutes;c_Clostridia;o_Clostridiales;f_;g_;s_*	**-2.80**	8.15E-03
310748	*p_Firmicutes;c_Clostridia;o_Clostridiales;f_;g_;s_*	**-2.48**	2.36E-02
354957	*p_Firmicutes;c_Clostridia;o_Clostridiales;f_;g_;s_*	**-2.16**	3.16E-02
313724	*p_Firmicutes;c_Clostridia;o_Clostridiales;f_;g_;s_*	**-2.10**	3.64E-02
332854	*p_Firmicutes;c_Clostridia;o_Clostridiales;f_;g_;s_*	**-1.98**	4.58E-02
195691	*p_Firmicutes;c_Clostridia;o_Clostridiales;f_Lachnospiraceae;g_;s_*	**3.65**	3.20E-04
450047	*p_Firmicutes;c_Clostridia;o_Clostridiales;f_Lachnospiraceae;g_;s_*	**2.06**	4.58E-02
2120784	*p_Firmicutes;c_Clostridia;o_Clostridiales;f_Lachnospiraceae;g_;s_*	**-2.95**	4.81E-03
323154	*p_Firmicutes;c_Clostridia;o_Clostridiales;f_Lachnospiraceae;g_;s_*	**-2.34**	2.64E-02
295075	*p_Firmicutes;c_Clostridia;o_Clostridiales;f_Lachnospiraceae;g_[Ruminococcus];s_gnavus*	**-2.72**	4.24E-03
339791	*p_Firmicutes;c_Clostridia;o_Clostridiales;f_Lachnospiraceae;g_Coprococcus;s_*	**-2.85**	1.13E-02
232283	*p_Firmicutes;c_Clostridia;o_Clostridiales;f_Lachnospiraceae;g_Coprococcus;s_*	**-2.27**	3.64E-02
441494	*p_Firmicutes;c_Clostridia;o_Clostridiales;f_Ruminococcaceae;g_;s_*	**2.84**	5.07E-04
193139	*p_Firmicutes;c_Clostridia;o_Clostridiales;f_Ruminococcaceae;g_;s_*	**-2.82**	1.13E-02
326609	*p_Firmicutes;c_Clostridia;o_Clostridiales;f_Ruminococcaceae;g_;s_*	**-1.84**	4.24E-02
163153	*p_Firmicutes;c_Clostridia;o_Clostridiales;f_Ruminococcaceae;g_Oscillospira;s_*	**2.20**	1.50E-02
262101	*p_Firmicutes;c_Erysipelotrichi;o_Erysipelotrichales;f_Erysipelotrichaceae;g_Allobaculum;s_*	**5.15**	1.09E-08
363731	*p_Verrucomicrobia;c_Verrucomicrobiae;o_Verrucomicrobiales;f_Verrucomicrobiaceae;g_Akkermansia;s_muciniphila*	**2.64**	3.66E-02

min **max** **-6.2** **+10.0**

B

Mice gavaged with *Lactobacillus helveticus* MIMLh5

Ileum

OTU nr.	Taxonomy	Mean normalized abundance PBS	Mean normalized abundance DG	log2 Fold Change	padj
276151	*p_Actinobacteria;c_Coriobacteriia;o_Coriobacteriales;f_Coriobacteriaceae;g_Adlercreutzia;s_*			**-2.42**	1.72E-02
228601	*p_Bacteroidetes;c_Bacteroidia;o_Bacteroidales;f_Bacteroidaceae;g_Bacteroides;s_*			**3.14**	5.14E-03
233587	*p_Bacteroidetes;c_Bacteroidia;o_Bacteroidales;f_S24-7;g_;s_*			**-3.66**	1.35E-03
339905	*p_Bacteroidetes;c_Bacteroidia;o_Bacteroidales;f_S24-7;g_;s_*			**-3.51**	3.81E-03
175706	*p_Bacteroidetes;c_Bacteroidia;o_Bacteroidales;f_S24-7;g_;s_*			**-3.47**	5.14E-03
423455	*p_Bacteroidetes;c_Bacteroidia;o_Bacteroidales;f_S24-7;g_;s_*			**-3.23**	5.14E-03
342962	*p_Bacteroidetes;c_Bacteroidia;o_Bacteroidales;f_S24-7;g_;s_*			**-3.19**	5.14E-03
331043	*p_Bacteroidetes;c_Bacteroidia;o_Bacteroidales;f_S24-7;g_;s_*			**-3.19**	3.81E-03
353012	*p_Bacteroidetes;c_Bacteroidia;o_Bacteroidales;f_S24-7;g_;s_*			**-2.91**	2.55E-02
188410	*p_Bacteroidetes;c_Bacteroidia;o_Bacteroidales;f_S24-7;g_;s_*			**-2.85**	1.89E-02
277120	*p_Bacteroidetes;c_Bacteroidia;o_Bacteroidales;f_S24-7;g_;s_*			**-2.76**	2.50E-02
334040	*p_Bacteroidetes;c_Bacteroidia;o_Bacteroidales;f_S24-7;g_;s_*			**-2.73**	1.08E-02
261350	*p_Bacteroidetes;c_Bacteroidia;o_Bacteroidales;f_S24-7;g_;s_*			**-2.69**	1.72E-02
356226	*p_Bacteroidetes;c_Bacteroidia;o_Bacteroidales;f_S24-7;g_;s_*			**-2.65**	1.98E-02
184381	*p_Bacteroidetes;c_Bacteroidia;o_Bacteroidales;f_S24-7;g_;s_*			**-2.63**	2.04E-02
430194	*p_Bacteroidetes;c_Bacteroidia;o_Bacteroidales;f_S24-7;g_;s_*			**-2.63**	2.09E-02
264657	*p_Bacteroidetes;c_Bacteroidia;o_Bacteroidales;f_S24-7;g_;s_*			**-2.53**	2.50E-02
353453	*p_Bacteroidetes;c_Bacteroidia;o_Bacteroidales;f_S24-7;g_;s_*			**-2.43**	4.16E-02
348038	*p_Bacteroidetes;c_Bacteroidia;o_Bacteroidales;f_S24-7;g_;s_*			**-2.37**	3.62E-02
263111	*p_Bacteroidetes;c_Bacteroidia;o_Bacteroidales;f_S24-7;g_;s_*			**-2.35**	2.55E-02
3231096	*p_Bacteroidetes;c_Bacteroidia;o_Bacteroidales;f_S24-7;g_;s_*			**-2.30**	2.77E-02
266860	*p_Bacteroidetes;c_Bacteroidia;o_Bacteroidales;f_S24-7;g_;s_*			**-2.27**	2.55E-02
263479	*p_Bacteroidetes;c_Bacteroidia;o_Bacteroidales;f_S24-7;g_;s_*			**-2.16**	4.83E-02
188427	*p_Bacteroidetes;c_Bacteroidia;o_Bacteroidales;f_S24-7;g_;s_*			**-2.06**	3.62E-02
182758	*p_Firmicutes;c_Bacilli;o_Lactobacillales;f_Lactobacillaceae;g_Lactobacillus;s_*			**-3.96**	1.15E-04
333178	*p_Firmicutes;c_Bacilli;o_Lactobacillales;f_Lactobacillaceae;g_Lactobacillus;s_*			**-3.92**	8.18E-04
463794	*p_Firmicutes;c_Bacilli;o_Lactobacillales;f_Lactobacillaceae;g_Lactobacillus;s_*			**-3.80**	1.03E-03
259111	*p_Firmicutes;c_Bacilli;o_Lactobacillales;f_Lactobacillaceae;g_Lactobacillus;s_*			**-3.22**	2.24E-03
452823	*p_Firmicutes;c_Bacilli;o_Lactobacillales;f_Lactobacillaceae;g_Lactobacillus;s_*			**-3.19**	2.24E-03
164664	*p_Firmicutes;c_Bacilli;o_Lactobacillales;f_Lactobacillaceae;g_Lactobacillus;s_*			**-2.71**	9.57E-03
242917	*p_Firmicutes;c_Bacilli;o_Lactobacillales;f_Lactobacillaceae;g_Lactobacillus;s_*			**-2.25**	2.55E-02
**807795**	***p_Firmicutes;c_Bacilli;o_Lactobacillales;f_Lactobacillaceae;g_Lactobacillus;s_helveticus* (strain MIMLh5)**			**4.49**	NA

Cecum

OTU nr.	Taxonomy	Mean normalized abundance PBS	Mean normalized abundance DG	log2 Fold Change	padj
178735	*p_Actinobacteria;c_Coriobacteriia;o_Coriobacteriales;f_Coriobacteriaceae;g_Adlercreutzia;s_*			**1.63**	2.33E-03
275974	*p_Actinobacteria;c_Coriobacteriia;o_Coriobacteriales;f_Coriobacteriaceae;g_Adlercreutzia;s_*			**1.40**	3.19E-02
3013444	*p_Bacteroidetes;c_Bacteroidia;o_Bacteroidales;f_;g_;s_*			**-1.78**	2.76E-03
583117	*p_Bacteroidetes;c_Bacteroidia;o_Bacteroidales;f_Bacteroidaceae;g_Bacteroides;s_*			**1.97**	1.03E-04
4378740	*p_Bacteroidetes;c_Bacteroidia;o_Bacteroidales;f_Prevotellaceae;g_Prevotella;s_*			**1.31**	1.96E-02
276218	*p_Bacteroidetes;c_Bacteroidia;o_Bacteroidales;f_S24-7;g_;s_*			**6.41**	4.32E-18
355746	*p_Bacteroidetes;c_Bacteroidia;o_Bacteroidales;f_S24-7;g_;s_*			**5.80**	4.57E-39
195919	*p_Bacteroidetes;c_Bacteroidia;o_Bacteroidales;f_S24-7;g_;s_*			**5.13**	1.72E-27
208409	*p_Bacteroidetes;c_Bacteroidia;o_Bacteroidales;f_S24-7;g_;s_*			**5.02**	2.84E-15
191789	*p_Bacteroidetes;c_Bacteroidia;o_Bacteroidales;f_S24-7;g_;s_*			**4.69**	1.12E-15
339549	*p_Bacteroidetes;c_Bacteroidia;o_Bacteroidales;f_S24-7;g_;s_*			**4.64**	7.12E-17
2212505	*p_Bacteroidetes;c_Bacteroidia;o_Bacteroidales;f_S24-7;g_;s_*			**4.49**	1.17E-11
331772	*p_Bacteroidetes;c_Bacteroidia;o_Bacteroidales;f_S24-7;g_;s_*			**4.36**	6.49E-20
421792	*p_Bacteroidetes;c_Bacteroidia;o_Bacteroidales;f_S24-7;g_;s_*			**4.35**	3.01E-11
264298	*p_Bacteroidetes;c_Bacteroidia;o_Bacteroidales;f_S24-7;g_;s_*			**4.29**	1.76E-12
215214	*p_Bacteroidetes;c_Bacteroidia;o_Bacteroidales;f_S24-7;g_;s_*			**4.24**	8.16E-10
174587	*p_Bacteroidetes;c_Bacteroidia;o_Bacteroidales;f_S24-7;g_;s_*			**4.18**	3.92E-13
217100	*p_Bacteroidetes;c_Bacteroidia;o_Bacteroidales;f_S24-7;g_;s_*			**4.18**	7.56E-12
206790	*p_Bacteroidetes;c_Bacteroidia;o_Bacteroidales;f_S24-7;g_;s_*			**3.98**	4.45E-14
330772	*p_Bacteroidetes;c_Bacteroidia;o_Bacteroidales;f_S24-7;g_;s_*			**3.72**	1.19E-13
275339	*p_Bacteroidetes;c_Bacteroidia;o_Bacteroidales;f_S24-7;g_;s_*			**3.56**	2.28E-12
215495	*p_Bacteroidetes;c_Bacteroidia;o_Bacteroidales;f_S24-7;g_;s_*			**3.53**	1.86E-10
247715	*p_Bacteroidetes;c_Bacteroidia;o_Bacteroidales;f_S24-7;g_;s_*			**3.46**	6.92E-07
341913	*p_Bacteroidetes;c_Bacteroidia;o_Bacteroidales;f_S24-7;g_;s_*			**3.39**	6.64E-09
189730	*p_Bacteroidetes;c_Bacteroidia;o_Bacteroidales;f_S24-7;g_;s_*			**3.37**	1.97E-06
343853	*p_Bacteroidetes;c_Bacteroidia;o_Bacteroidales;f_S24-7;g_;s_*			**3.34**	1.46E-07
346870	*p_Bacteroidetes;c_Bacteroidia;o_Bacteroidales;f_S24-7;g_;s_*			**3.31**	1.05E-06
198644	*p_Bacteroidetes;c_Bacteroidia;o_Bacteroidales;f_S24-7;g_;s_*			**3.30**	6.92E-07
191749	*p_Bacteroidetes;c_Bacteroidia;o_Bacteroidales;f_S24-7;g_;s_*			**3.28**	5.62E-06
175080	*p_Bacteroidetes;c_Bacteroidia;o_Bacteroidales;f_S24-7;g_;s_*			**3.25**	1.69E-05
210665	*p_Bacteroidetes;c_Bacteroidia;o_Bacteroidales;f_S24-7;g_;s_*			**3.20**	4.77E-07
210383	*p_Bacteroidetes;c_Bacteroidia;o_Bacteroidales;f_S24-7;g_;s_*			**3.16**	2.75E-05
233435	*p_Bacteroidetes;c_Bacteroidia;o_Bacteroidales;f_S24-7;g_;s_*			**3.11**	6.92E-07
384555	*p_Bacteroidetes;c_Bacteroidia;o_Bacteroidales;f_S24-7;g_;s_*			**3.09**	6.79E-06
389282	*p_Bacteroidetes;c_Bacteroidia;o_Bacteroidales;f_S24-7;g_;s_*			**2.95**	8.00E-10
203605	*p_Bacteroidetes;c_Bacteroidia;o_Bacteroidales;f_S24-7;g_;s_*			**2.88**	8.21E-06
194048	*p_Bacteroidetes;c_Bacteroidia;o_Bacteroidales;f_S24-7;g_;s_*			**2.66**	6.48E-04

264734	p_Bacteroidetes;c_Bacteroidia;o_Bacteroidales;f_S24-7;g_;s_	**2.59**	2.28E-05
208280	p_Bacteroidetes;c_Bacteroidia;o_Bacteroidales;f_S24-7;g_;s_	**2.45**	2.07E-03
326095	p_Bacteroidetes;c_Bacteroidia;o_Bacteroidales;f_S24-7;g_;s_	**2.44**	9.10E-03
316629	p_Bacteroidetes;c_Bacteroidia;o_Bacteroidales;f_S24-7;g_;s_	**2.39**	2.00E-03
204171	p_Bacteroidetes;c_Bacteroidia;o_Bacteroidales;f_S24-7;g_;s_	**2.35**	7.09E-03
177435	p_Bacteroidetes;c_Bacteroidia;o_Bacteroidales;f_S24-7;g_;s_	**2.25**	1.11E-02
215897	p_Bacteroidetes;c_Bacteroidia;o_Bacteroidales;f_S24-7;g_;s_	**2.20**	1.28E-02
352789	p_Bacteroidetes;c_Bacteroidia;o_Bacteroidales;f_S24-7;g_;s_	**2.19**	4.52E-04
177269	p_Bacteroidetes;c_Bacteroidia;o_Bacteroidales;f_S24-7;g_;s_	**2.17**	8.13E-03
372368	p_Bacteroidetes;c_Bacteroidia;o_Bacteroidales;f_S24-7;g_;s_	**2.14**	2.35E-05
801260	p_Bacteroidetes;c_Bacteroidia;o_Bacteroidales;f_S24-7;g_;s_	**2.10**	4.87E-04
269673	p_Bacteroidetes;c_Bacteroidia;o_Bacteroidales;f_S24-7;g_;s_	**2.03**	2.33E-03
209446	p_Bacteroidetes;c_Bacteroidia;o_Bacteroidales;f_S24-7;g_;s_	**2.02**	1.55E-02
416078	p_Bacteroidetes;c_Bacteroidia;o_Bacteroidales;f_S24-7;g_;s_	**1.91**	1.10E-04
174754	p_Bacteroidetes;c_Bacteroidia;o_Bacteroidales;f_S24-7;g_;s_	**1.89**	1.32E-02
338258	p_Bacteroidetes;c_Bacteroidia;o_Bacteroidales;f_S24-7;g_;s_	**1.88**	8.92E-04
427241	p_Bacteroidetes;c_Bacteroidia;o_Bacteroidales;f_S24-7;g_;s_	**1.87**	1.96E-02
211494	p_Bacteroidetes;c_Bacteroidia;o_Bacteroidales;f_S24-7;g_;s_	**1.85**	4.61E-04
192494	p_Bacteroidetes;c_Bacteroidia;o_Bacteroidales;f_S24-7;g_;s_	**1.82**	1.56E-03
206456	p_Bacteroidetes;c_Bacteroidia;o_Bacteroidales;f_S24-7;g_;s_	**1.81**	1.22E-04
269726	p_Bacteroidetes;c_Bacteroidia;o_Bacteroidales;f_S24-7;g_;s_	**1.76**	3.21E-03
216495	p_Bacteroidetes;c_Bacteroidia;o_Bacteroidales;f_S24-7;g_;s_	**1.75**	8.12E-03
174791	p_Bacteroidetes;c_Bacteroidia;o_Bacteroidales;f_S24-7;g_;s_	**1.71**	4.48E-02
320169	p_Bacteroidetes;c_Bacteroidia;o_Bacteroidales;f_S24-7;g_;s_	**1.71**	1.65E-03
348088	p_Bacteroidetes;c_Bacteroidia;o_Bacteroidales;f_S24-7;g_;s_	**1.70**	3.43E-04
349175	p_Bacteroidetes;c_Bacteroidia;o_Bacteroidales;f_S24-7;g_;s_	**1.65**	9.09E-03
398255	p_Bacteroidetes;c_Bacteroidia;o_Bacteroidales;f_S24-7;g_;s_	**1.63**	8.12E-03
263479	p_Bacteroidetes;c_Bacteroidia;o_Bacteroidales;f_S24-7;g_;s_	**1.43**	3.34E-02
319525	p_Bacteroidetes;c_Bacteroidia;o_Bacteroidales;f_S24-7;g_;s_	**1.37**	3.31E-02
196385	p_Bacteroidetes;c_Bacteroidia;o_Bacteroidales;f_S24-7;g_;s_	**1.25**	1.92E-02
182849	p_Bacteroidetes;c_Bacteroidia;o_Bacteroidales;f_S24-7;g_;s_	**1.12**	3.19E-02
175706	p_Bacteroidetes;c_Bacteroidia;o_Bacteroidales;f_S24-7;g_;s_	**-1.21**	4.48E-02
188410	p_Bacteroidetes;c_Bacteroidia;o_Bacteroidales;f_S24-7;g_;s_	**-1.43**	1.37E-02
381666	p_Cyanobacteria;c_4C0d-2;o_YS2;f_;g_;s_	**2.34**	8.76E-03
813944	p_Firmicutes;c_Bacilli;o_Lactobacillales;f_Lactobacillaceae;g_Lactobacillus;s_	**8.62**	7.65E-38
549991	p_Firmicutes;c_Bacilli;o_Lactobacillales;f_Lactobacillaceae;g_Lactobacillus;s_	**7.95**	1.62E-31
538223	p_Firmicutes;c_Bacilli;o_Lactobacillales;f_Lactobacillaceae;g_Lactobacillus;s_	**7.83**	2.36E-33
302975	p_Firmicutes;c_Bacilli;o_Lactobacillales;f_Lactobacillaceae;g_Lactobacillus;s_	**6.78**	4.08E-23
146935	p_Firmicutes;c_Bacilli;o_Lactobacillales;f_Lactobacillaceae;g_Lactobacillus;s_	**5.90**	1.41E-16
589114	p_Firmicutes;c_Bacilli;o_Lactobacillales;f_Lactobacillaceae;g_Lactobacillus;s_	**4.80**	3.01E-11
255367	p_Firmicutes;c_Bacilli;o_Lactobacillales;f_Lactobacillaceae;g_Lactobacillus;s_	**4.61**	3.79E-10
806179	p_Firmicutes;c_Bacilli;o_Lactobacillales;f_Lactobacillaceae;g_Lactobacillus;s_	**4.61**	3.59E-10
584571	p_Firmicutes;c_Bacilli;o_Lactobacillales;f_Lactobacillaceae;g_Lactobacillus;s_	**4.46**	6.64E-09
456393	p_Firmicutes;c_Bacilli;o_Lactobacillales;f_Lactobacillaceae;g_Lactobacillus;s_	**3.81**	5.60E-07
749329	p_Firmicutes;c_Bacilli;o_Lactobacillales;f_Lactobacillaceae;g_Lactobacillus;s_	**3.75**	5.91E-07
809106	p_Firmicutes;c_Bacilli;o_Lactobacillales;f_Lactobacillaceae;g_Lactobacillus;s_	**3.49**	7.59E-06
821597	p_Firmicutes;c_Bacilli;o_Lactobacillales;f_Lactobacillaceae;g_Lactobacillus;s_	**3.46**	7.59E-06
819507	p_Firmicutes;c_Bacilli;o_Lactobacillales;f_Lactobacillaceae;g_Lactobacillus;s_	**3.25**	1.01E-04
851794	p_Firmicutes;c_Bacilli;o_Lactobacillales;f_Lactobacillaceae;g_Lactobacillus;s_	**3.25**	1.42E-07
586093	p_Firmicutes;c_Bacilli;o_Lactobacillales;f_Lactobacillaceae;g_Lactobacillus;s_	**3.18**	6.16E-05
463361	p_Firmicutes;c_Bacilli;o_Lactobacillales;f_Lactobacillaceae;g_Lactobacillus;s_	**3.14**	1.32E-04
823948	p_Firmicutes;c_Bacilli;o_Lactobacillales;f_Lactobacillaceae;g_Lactobacillus;s_	**3.10**	7.53E-05
131702	p_Firmicutes;c_Bacilli;o_Lactobacillales;f_Lactobacillaceae;g_Lactobacillus;s_	**3.01**	1.59E-04
537734	p_Firmicutes;c_Bacilli;o_Lactobacillales;f_Lactobacillaceae;g_Lactobacillus;s_	**2.75**	8.63E-04
137580	p_Firmicutes;c_Bacilli;o_Lactobacillales;f_Lactobacillaceae;g_Lactobacillus;s_	**2.40**	4.53E-03
4321285	p_Firmicutes;c_Bacilli;o_Lactobacillales;f_Lactobacillaceae;g_Lactobacillus;s_	**2.04**	2.24E-02
274016	p_Firmicutes;c_Bacilli;o_Lactobacillales;f_Lactobacillaceae;g_Lactobacillus;s_	**-2.07**	5.10E-03
1107027	p_Firmicutes;c_Bacilli;o_Lactobacillales;f_Lactobacillaceae;g_Lactobacillus;s_	**-2.15**	1.12E-02
452823	p_Firmicutes;c_Bacilli;o_Lactobacillales;f_Lactobacillaceae;g_Lactobacillus;s_	**-2.21**	4.67E-04
463794	p_Firmicutes;c_Bacilli;o_Lactobacillales;f_Lactobacillaceae;g_Lactobacillus;s_	**-2.56**	1.36E-06
259111	p_Firmicutes;c_Bacilli;o_Lactobacillales;f_Lactobacillaceae;g_Lactobacillus;s_	**-2.65**	3.57E-04
182758	p_Firmicutes;c_Bacilli;o_Lactobacillales;f_Lactobacillaceae;g_Lactobacillus;s_	**-2.70**	6.34E-06
333178	p_Firmicutes;c_Bacilli;o_Lactobacillales;f_Lactobacillaceae;g_Lactobacillus;s_	**-2.72**	5.70E-07
164664	p_Firmicutes;c_Bacilli;o_Lactobacillales;f_Lactobacillaceae;g_Lactobacillus;s_	**-2.80**	2.05E-04
**807795**	***p_Firmicutes;c_Bacilli;o_Lactobacillales;f_Lactobacillaceae;g_Lactobacillus;s_helveticus*** **(strain MIMLh5)**	**9.41**	1.97E-109
214919	p_Firmicutes;c_Bacilli;o_Turicibacterales;f_Turicibacteraceae;g_Turicibacter;s_	**2.82**	1.57E-03
276305	p_Firmicutes;c_Clostridia;o_Clostridiales;f_;g_;s_	**6.13**	7.12E-17
330460	p_Firmicutes;c_Clostridia;o_Clostridiales;f_;g_;s_	**4.86**	8.01E-09
675863	p_Firmicutes;c_Clostridia;o_Clostridiales;f_;g_;s_	**4.55**	5.62E-09
276770	p_Firmicutes;c_Clostridia;o_Clostridiales;f_;g_;s_	**4.30**	1.36E-06
354501	p_Firmicutes;c_Clostridia;o_Clostridiales;f_;g_;s_	**3.86**	6.52E-06
353657	p_Firmicutes;c_Clostridia;o_Clostridiales;f_;g_;s_	**3.80**	7.18E-06
185334	p_Firmicutes;c_Clostridia;o_Clostridiales;f_;g_;s_	**3.52**	5.49E-06
352049	p_Firmicutes;c_Clostridia;o_Clostridiales;f_;g_;s_	**3.35**	2.29E-05
275139	p_Firmicutes;c_Clostridia;o_Clostridiales;f_;g_;s_	**3.26**	2.02E-06
277068	p_Firmicutes;c_Clostridia;o_Clostridiales;f_;g_;s_	**3.23**	3.61E-06
833816	p_Firmicutes;c_Clostridia;o_Clostridiales;f_;g_;s_	**3.18**	2.34E-04
4383135	p_Firmicutes;c_Clostridia;o_Clostridiales;f_;g_;s_	**3.12**	5.88E-05
354032	p_Firmicutes;c_Clostridia;o_Clostridiales;f_;g_;s_	**3.05**	9.35E-05
272080	p_Firmicutes;c_Clostridia;o_Clostridiales;f_;g_;s_	**2.88**	8.63E-04
229562	p_Firmicutes;c_Clostridia;o_Clostridiales;f_;g_;s_	**2.87**	3.35E-03
351593	p_Firmicutes;c_Clostridia;o_Clostridiales;f_;g_;s_	**2.80**	1.57E-03
324865	p_Firmicutes;c_Clostridia;o_Clostridiales;f_;g_;s_	**2.71**	3.21E-03
276674	p_Firmicutes;c_Clostridia;o_Clostridiales;f_;g_;s_	**2.66**	5.53E-04

275180	*p_Firmicutes;c_Clostridia;o_Clostridiales;f_;g_;s_*	**2.60**	4.53E-03
199215	*p_Firmicutes;c_Clostridia;o_Clostridiales;f_;g_;s_*	**2.56**	8.82E-05
340706	*p_Firmicutes;c_Clostridia;o_Clostridiales;f_;g_;s_*	**2.49**	8.25E-04
329712	*p_Firmicutes;c_Clostridia;o_Clostridiales;f_;g_;s_*	**2.48**	7.48E-05
830189	*p_Firmicutes;c_Clostridia;o_Clostridiales;f_;g_;s_*	**2.30**	1.66E-02
354662	*p_Firmicutes;c_Clostridia;o_Clostridiales;f_;g_;s_*	**2.30**	2.64E-03
4480555	*p_Firmicutes;c_Clostridia;o_Clostridiales;f_;g_;s_*	**2.23**	5.40E-03
610842	*p_Firmicutes;c_Clostridia;o_Clostridiales;f_;g_;s_*	**2.19**	1.29E-02
194787	*p_Firmicutes;c_Clostridia;o_Clostridiales;f_;g_;s_*	**2.07**	1.87E-02
269035	*p_Firmicutes;c_Clostridia;o_Clostridiales;f_;g_;s_*	**2.05**	2.20E-03
831409	*p_Firmicutes;c_Clostridia;o_Clostridiales;f_;g_;s_*	**2.04**	1.74E-02
1107461	*p_Firmicutes;c_Clostridia;o_Clostridiales;f_;g_;s_*	**2.02**	2.16E-02
460611	*p_Firmicutes;c_Clostridia;o_Clostridiales;f_;g_;s_*	**1.90**	1.38E-02
352612	*p_Firmicutes;c_Clostridia;o_Clostridiales;f_;g_;s_*	**1.89**	1.24E-02
390820	*p_Firmicutes;c_Clostridia;o_Clostridiales;f_;g_;s_*	**1.83**	4.27E-02
2883968	*p_Firmicutes;c_Clostridia;o_Clostridiales;f_;g_;s_*	**1.81**	3.64E-02
273479	*p_Firmicutes;c_Clostridia;o_Clostridiales;f_;g_;s_*	**1.81**	3.19E-02
1107674	*p_Firmicutes;c_Clostridia;o_Clostridiales;f_;g_;s_*	**1.76**	2.60E-02
183211	*p_Firmicutes;c_Clostridia;o_Clostridiales;f_;g_;s_*	**1.68**	3.75E-02
50208	*p_Firmicutes;c_Clostridia;o_Clostridiales;f_;g_;s_*	**1.57**	2.67E-02
314810	*p_Firmicutes;c_Clostridia;o_Clostridiales;f_;g_;s_*	**1.57**	1.30E-02
298408	*p_Firmicutes;c_Clostridia;o_Clostridiales;f_;g_;s_*	**1.54**	3.24E-02
309249	*p_Firmicutes;c_Clostridia;o_Clostridiales;f_;g_;s_*	**1.52**	4.02E-02
197790	*p_Firmicutes;c_Clostridia;o_Clostridiales;f_;g_;s_*	**1.51**	3.19E-02
339886	*p_Firmicutes;c_Clostridia;o_Clostridiales;f_;g_;s_*	**1.50**	1.53E-02
173739	*p_Firmicutes;c_Clostridia;o_Clostridiales;f_;g_;s_*	**1.46**	3.15E-02
354957	*p_Firmicutes;c_Clostridia;o_Clostridiales;f_;g_;s_*	**-1.41**	3.44E-02
353923	*p_Firmicutes;c_Clostridia;o_Clostridiales;f_;g_;s_*	**-1.49**	1.29E-02
336276	*p_Firmicutes;c_Clostridia;o_Clostridiales;f_;g_;s_*	**-1.63**	4.20E-02
330064	*p_Firmicutes;c_Clostridia;o_Clostridiales;f_;g_;s_*	**-1.83**	3.44E-02
335267	*p_Firmicutes;c_Clostridia;o_Clostridiales;f_;g_;s_*	**-1.84**	1.24E-02
310748	*p_Firmicutes;c_Clostridia;o_Clostridiales;f_;g_;s_*	**-2.14**	3.02E-02
665703	*p_Firmicutes;c_Clostridia;o_Clostridiales;f_;g_;s_*	**-2.26**	2.77E-02
1844565	*p_Firmicutes;c_Clostridia;o_Clostridiales;f_;g_;s_*	**-2.31**	4.77E-03
797021	*p_Firmicutes;c_Clostridia;o_Clostridiales;f_;g_;s_*	**-2.32**	1.27E-02
331117	*p_Firmicutes;c_Clostridia;o_Clostridiales;f_;g_;s_*	**-2.44**	6.36E-03
182079	*p_Firmicutes;c_Clostridia;o_Clostridiales;f_;g_;s_*	**-2.49**	8.12E-03
198271	*p_Firmicutes;c_Clostridia;o_Clostridiales;f_;g_;s_*	**-2.57**	1.39E-02
138338	*p_Firmicutes;c_Clostridia;o_Clostridiales;f_;g_;s_*	**-2.58**	1.83E-03
212732	*p_Firmicutes;c_Clostridia;o_Clostridiales;f_;g_;s_*	**-2.66**	4.77E-03
261097	*p_Firmicutes;c_Clostridia;o_Clostridiales;f_;g_;s_*	**-4.07**	1.72E-04
199532	*p_Firmicutes;c_Clostridia;o_Clostridiales;f_;g_;s_*	**-4.31**	3.37E-06
4365109	*p_Firmicutes;c_Clostridia;o_Clostridiales;f_;g_;s_*	**-5.74**	2.35E-08
178738	*p_Firmicutes;c_Clostridia;o_Clostridiales;f_Clostridiaceae;g_;s_*	**1.76**	1.24E-02
261084	*p_Firmicutes;c_Clostridia;o_Clostridiales;f_Clostridiaceae;g_;s_*	**1.56**	3.92E-02
581463	*p_Firmicutes;c_Clostridia;o_Clostridiales;f_Clostridiaceae;g_Clostridium;s_*	**1.64**	4.42E-02
555945	*p_Firmicutes;c_Clostridia;o_Clostridiales;f_Clostridiaceae;g_SMB53;s_*	**2.34**	5.25E-03
514988	*p_Firmicutes;c_Clostridia;o_Clostridiales;f_Clostridiaceae;g_SMB53;s_*	**1.75**	2.75E-02
265018	*p_Firmicutes;c_Clostridia;o_Clostridiales;f_Lachnospiraceae;g_;s_*	**3.89**	5.35E-05
838200	*p_Firmicutes;c_Clostridia;o_Clostridiales;f_Lachnospiraceae;g_;s_*	**3.75**	9.92E-07
4471525	*p_Firmicutes;c_Clostridia;o_Clostridiales;f_Lachnospiraceae;g_;s_*	**3.19**	7.48E-05
450047	*p_Firmicutes;c_Clostridia;o_Clostridiales;f_Lachnospiraceae;g_;s_*	**2.57**	3.44E-03
305177	*p_Firmicutes;c_Clostridia;o_Clostridiales;f_Lachnospiraceae;g_;s_*	**2.43**	5.88E-03
310760	*p_Firmicutes;c_Clostridia;o_Clostridiales;f_Lachnospiraceae;g_;s_*	**2.16**	5.50E-04
351881	*p_Firmicutes;c_Clostridia;o_Clostridiales;f_Lachnospiraceae;g_;s_*	**2.14**	3.30E-03
4440360	*p_Firmicutes;c_Clostridia;o_Clostridiales;f_Lachnospiraceae;g_;s_*	**2.13**	1.57E-02
345709	*p_Firmicutes;c_Clostridia;o_Clostridiales;f_Lachnospiraceae;g_;s_*	**1.87**	2.01E-02
268538	*p_Firmicutes;c_Clostridia;o_Clostridiales;f_Lachnospiraceae;g_;s_*	**1.53**	4.71E-02
658370	*p_Firmicutes;c_Clostridia;o_Clostridiales;f_Lachnospiraceae;g_;s_*	**1.40**	4.73E-02
269657	*p_Firmicutes;c_Clostridia;o_Clostridiales;f_Lachnospiraceae;g_;s_*	**1.29**	4.14E-02
258169	*p_Firmicutes;c_Clostridia;o_Clostridiales;f_Lachnospiraceae;g_;s_*	**-1.66**	1.11E-02
334619	*p_Firmicutes;c_Clostridia;o_Clostridiales;f_Lachnospiraceae;g_;s_*	**-1.92**	2.46E-02
339229	*p_Firmicutes;c_Clostridia;o_Clostridiales;f_Lachnospiraceae;g_;s_*	**-2.92**	2.03E-03
267298	*p_Firmicutes;c_Clostridia;o_Clostridiales;f_Lachnospiraceae;g_[Ruminococcus];s_gnavus*	**2.83**	1.03E-04
1107057	*p_Firmicutes;c_Clostridia;o_Clostridiales;f_Lachnospiraceae;g_Coprococcus;s_*	**3.45**	2.01E-06
338926	*p_Firmicutes;c_Clostridia;o_Clostridiales;f_Lachnospiraceae;g_Dorea;s_*	**-1.62**	4.71E-02
276478	*p_Firmicutes;c_Clostridia;o_Clostridiales;f_Peptostreptococcaceae;g_;s_*	**2.32**	5.45E-03
227622	*p_Firmicutes;c_Clostridia;o_Clostridiales;f_Ruminococcaceae;g_;s_*	**2.78**	1.82E-04
171396	*p_Firmicutes;c_Clostridia;o_Clostridiales;f_Ruminococcaceae;g_;s_*	**2.73**	1.10E-04
176582	*p_Firmicutes;c_Clostridia;o_Clostridiales;f_Ruminococcaceae;g_;s_*	**2.21**	1.17E-03
4338227	*p_Firmicutes;c_Clostridia;o_Clostridiales;f_Ruminococcaceae;g_;s_*	**2.18**	5.28E-03
331737	*p_Firmicutes;c_Clostridia;o_Clostridiales;f_Ruminococcaceae;g_;s_*	**2.12**	3.87E-03
1111350	*p_Firmicutes;c_Clostridia;o_Clostridiales;f_Ruminococcaceae;g_;s_*	**2.12**	3.34E-03
583134	*p_Firmicutes;c_Clostridia;o_Clostridiales;f_Ruminococcaceae;g_;s_*	**1.92**	1.58E-02
321960	*p_Firmicutes;c_Clostridia;o_Clostridiales;f_Ruminococcaceae;g_;s_*	**1.81**	3.19E-02
278901	*p_Firmicutes;c_Clostridia;o_Clostridiales;f_Ruminococcaceae;g_;s_*	**1.68**	3.34E-02
336691	*p_Firmicutes;c_Clostridia;o_Clostridiales;f_Ruminococcaceae;g_;s_*	**1.55**	3.64E-02
443620	*p_Firmicutes;c_Clostridia;o_Clostridiales;f_Ruminococcaceae;g_Oscillospira;s_*	**2.68**	8.55E-06
1569569	*p_Firmicutes;c_Clostridia;o_Clostridiales;f_Ruminococcaceae;g_Oscillospira;s_*	**2.09**	8.76E-03
327808	*p_Firmicutes;c_Clostridia;o_Clostridiales;f_Ruminococcaceae;g_Oscillospira;s_*	**1.67**	1.83E-03
330333	*p_Firmicutes;c_Clostridia;o_Clostridiales;f_Ruminococcaceae;g_Oscillospira;s_*	**1.39**	4.73E-02
350404	*p_Firmicutes;c_Clostridia;o_Clostridiales;f_Ruminococcaceae;g_Oscillospira;s_*	**1.34**	1.87E-02
329790	*p_Firmicutes;c_Clostridia;o_Clostridiales;f_Ruminococcaceae;g_Oscillospira;s_*	**1.26**	4.21E-02
408513	*p_Firmicutes;c_Clostridia;o_Clostridiales;f_Ruminococcaceae;g_Oscillospira;s_*	**1.23**	2.21E-02
827592	*p_Firmicutes;c_Clostridia;o_Clostridiales;f_Ruminococcaceae;g_Ruminococcus;s_*	**1.68**	1.09E-02

828435	*p_Firmicutes;c_Erysipelotrichi;o_Erysipelotrichales;f_Erysipelotrichaceae;g_;s_*			**1.57**	8.13E-03
262101	*p_Firmicutes;c_Erysipelotrichi;o_Erysipelotrichales;f_Erysipelotrichaceae;g_Allobaculum;s_*			**3.30**	8.78E-06
437137	*p_Proteobacteria;c_Betaproteobacteria;o_Burkholderiales;f_Alcaligenaceae;g_Sutterella;s_*			**3.40**	1.05E-08
359809	*p_Proteobacteria;c_Betaproteobacteria;o_Burkholderiales;f_Alcaligenaceae;g_Sutterella;s_*			**1.47**	2.92E-02

Colon

OTU nr.	Taxonomy	Mean normalized abundance PBS	Mean normalized abundance DG	log2 Fold Change	padj
3013444	*p_Bacteroidetes;c_Bacteroidia;o_Bacteroidales;f_;g_;s_*			**-1.46**	3.83E-02
3028318	*p_Bacteroidetes;c_Bacteroidia;o_Bacteroidales;f_Rikenellaceae;g_;s_*			**1.60**	4.42E-02
276218	*p_Bacteroidetes;c_Bacteroidia;o_Bacteroidales;f_S24-7;g_;s_*			**5.91**	4.77E-13
331772	*p_Bacteroidetes;c_Bacteroidia;o_Bacteroidales;f_S24-7;g_;s_*			**5.74**	1.09E-16
217100	*p_Bacteroidetes;c_Bacteroidia;o_Bacteroidales;f_S24-7;g_;s_*			**4.93**	9.26E-10
215214	*p_Bacteroidetes;c_Bacteroidia;o_Bacteroidales;f_S24-7;g_;s_*			**4.80**	7.64E-12
2212505	*p_Bacteroidetes;c_Bacteroidia;o_Bacteroidales;f_S24-7;g_;s_*			**4.61**	1.77E-09
174587	*p_Bacteroidetes;c_Bacteroidia;o_Bacteroidales;f_S24-7;g_;s_*			**4.61**	7.65E-12
355746	*p_Bacteroidetes;c_Bacteroidia;o_Bacteroidales;f_S24-7;g_;s_*			**4.57**	9.01E-14
215495	*p_Bacteroidetes;c_Bacteroidia;o_Bacteroidales;f_S24-7;g_;s_*			**4.57**	6.30E-10
421792	*p_Bacteroidetes;c_Bacteroidia;o_Bacteroidales;f_S24-7;g_;s_*			**4.56**	3.44E-13
208409	*p_Bacteroidetes;c_Bacteroidia;o_Bacteroidales;f_S24-7;g_;s_*			**4.48**	9.78E-09
206790	*p_Bacteroidetes;c_Bacteroidia;o_Bacteroidales;f_S24-7;g_;s_*			**4.42**	4.88E-10
339549	*p_Bacteroidetes;c_Bacteroidia;o_Bacteroidales;f_S24-7;g_;s_*			**4.39**	6.83E-12
195919	*p_Bacteroidetes;c_Bacteroidia;o_Bacteroidales;f_S24-7;g_;s_*			**4.27**	7.64E-12
233435	*p_Bacteroidetes;c_Bacteroidia;o_Bacteroidales;f_S24-7;g_;s_*			**4.26**	3.61E-10
343853	*p_Bacteroidetes;c_Bacteroidia;o_Bacteroidales;f_S24-7;g_;s_*			**4.24**	1.93E-10
346870	*p_Bacteroidetes;c_Bacteroidia;o_Bacteroidales;f_S24-7;g_;s_*			**4.23**	1.69E-08
264298	*p_Bacteroidetes;c_Bacteroidia;o_Bacteroidales;f_S24-7;g_;s_*			**4.17**	3.37E-09
341913	*p_Bacteroidetes;c_Bacteroidia;o_Bacteroidales;f_S24-7;g_;s_*			**4.13**	9.92E-09
326095	*p_Bacteroidetes;c_Bacteroidia;o_Bacteroidales;f_S24-7;g_;s_*			**4.05**	5.15E-08
189730	*p_Bacteroidetes;c_Bacteroidia;o_Bacteroidales;f_S24-7;g_;s_*			**4.00**	5.28E-06
191789	*p_Bacteroidetes;c_Bacteroidia;o_Bacteroidales;f_S24-7;g_;s_*			**3.99**	5.78E-10
330772	*p_Bacteroidetes;c_Bacteroidia;o_Bacteroidales;f_S24-7;g_;s_*			**3.89**	1.50E-11
210665	*p_Bacteroidetes;c_Bacteroidia;o_Bacteroidales;f_S24-7;g_;s_*			**3.88**	4.36E-07
384555	*p_Bacteroidetes;c_Bacteroidia;o_Bacteroidales;f_S24-7;g_;s_*			**3.75**	1.56E-05
198644	*p_Bacteroidetes;c_Bacteroidia;o_Bacteroidales;f_S24-7;g_;s_*			**3.69**	2.62E-05
203605	*p_Bacteroidetes;c_Bacteroidia;o_Bacteroidales;f_S24-7;g_;s_*			**3.56**	3.48E-05
275339	*p_Bacteroidetes;c_Bacteroidia;o_Bacteroidales;f_S24-7;g_;s_*			**3.50**	1.21E-08
264734	*p_Bacteroidetes;c_Bacteroidia;o_Bacteroidales;f_S24-7;g_;s_*			**3.35**	1.67E-06
389282	*p_Bacteroidetes;c_Bacteroidia;o_Bacteroidales;f_S24-7;g_;s_*			**3.35**	6.16E-06
247715	*p_Bacteroidetes;c_Bacteroidia;o_Bacteroidales;f_S24-7;g_;s_*			**3.32**	2.19E-05
194048	*p_Bacteroidetes;c_Bacteroidia;o_Bacteroidales;f_S24-7;g_;s_*			**3.26**	3.05E-04
175272	*p_Bacteroidetes;c_Bacteroidia;o_Bacteroidales;f_S24-7;g_;s_*			**2.95**	4.62E-03
175646	*p_Bacteroidetes;c_Bacteroidia;o_Bacteroidales;f_S24-7;g_;s_*			**2.92**	1.14E-03
210383	*p_Bacteroidetes;c_Bacteroidia;o_Bacteroidales;f_S24-7;g_;s_*			**2.87**	4.59E-04
352789	*p_Bacteroidetes;c_Bacteroidia;o_Bacteroidales;f_S24-7;g_;s_*			**2.85**	7.80E-06
269726	*p_Bacteroidetes;c_Bacteroidia;o_Bacteroidales;f_S24-7;g_;s_*			**2.83**	4.13E-04
316629	*p_Bacteroidetes;c_Bacteroidia;o_Bacteroidales;f_S24-7;g_;s_*			**2.82**	2.23E-03
191749	*p_Bacteroidetes;c_Bacteroidia;o_Bacteroidales;f_S24-7;g_;s_*			**2.67**	1.17E-03
174805	*p_Bacteroidetes;c_Bacteroidia;o_Bacteroidales;f_S24-7;g_;s_*			**2.64**	9.94E-03
177917	*p_Bacteroidetes;c_Bacteroidia;o_Bacteroidales;f_S24-7;g_;s_*			**2.58**	8.86E-03
175080	*p_Bacteroidetes;c_Bacteroidia;o_Bacteroidales;f_S24-7;g_;s_*			**2.51**	4.75E-03
269673	*p_Bacteroidetes;c_Bacteroidia;o_Bacteroidales;f_S24-7;g_;s_*			**2.40**	2.94E-03
177269	*p_Bacteroidetes;c_Bacteroidia;o_Bacteroidales;f_S24-7;g_;s_*			**2.38**	1.24E-02
349175	*p_Bacteroidetes;c_Bacteroidia;o_Bacteroidales;f_S24-7;g_;s_*			**2.29**	3.48E-03
801260	*p_Bacteroidetes;c_Bacteroidia;o_Bacteroidales;f_S24-7;g_;s_*			**2.16**	3.33E-03
338258	*p_Bacteroidetes;c_Bacteroidia;o_Bacteroidales;f_S24-7;g_;s_*			**2.16**	6.77E-03
206456	*p_Bacteroidetes;c_Bacteroidia;o_Bacteroidales;f_S24-7;g_;s_*			**2.16**	1.89E-03
177435	*p_Bacteroidetes;c_Bacteroidia;o_Bacteroidales;f_S24-7;g_;s_*			**2.13**	2.74E-02
215897	*p_Bacteroidetes;c_Bacteroidia;o_Bacteroidales;f_S24-7;g_;s_*			**2.10**	2.83E-02
209408	*p_Bacteroidetes;c_Bacteroidia;o_Bacteroidales;f_S24-7;g_;s_*			**2.01**	4.04E-02
204088	*p_Bacteroidetes;c_Bacteroidia;o_Bacteroidales;f_S24-7;g_;s_*			**1.95**	3.97E-02
379505	*p_Bacteroidetes;c_Bacteroidia;o_Bacteroidales;f_S24-7;g_;s_*			**1.95**	3.99E-02
211494	*p_Bacteroidetes;c_Bacteroidia;o_Bacteroidales;f_S24-7;g_;s_*			**1.87**	1.70E-02
348088	*p_Bacteroidetes;c_Bacteroidia;o_Bacteroidales;f_S24-7;g_;s_*			**1.83**	3.73E-03
372368	*p_Bacteroidetes;c_Bacteroidia;o_Bacteroidales;f_S24-7;g_;s_*			**1.82**	4.62E-03
216495	*p_Bacteroidetes;c_Bacteroidia;o_Bacteroidales;f_S24-7;g_;s_*			**1.67**	3.51E-02
175458	*p_Bacteroidetes;c_Bacteroidia;o_Bacteroidales;f_S24-7;g_;s_*			**1.59**	4.83E-02
192494	*p_Bacteroidetes;c_Bacteroidia;o_Bacteroidales;f_S24-7;g_;s_*			**1.58**	2.56E-02
416078	*p_Bacteroidetes;c_Bacteroidia;o_Bacteroidales;f_S24-7;g_;s_*			**1.57**	8.85E-03
196385	*p_Bacteroidetes;c_Bacteroidia;o_Bacteroidales;f_S24-7;g_;s_*			**1.21**	4.79E-02
381666	*p_Cyanobacteria;c_4C0d-2;o_YS2;f_;g_;s_*			**2.69**	2.60E-02
813944	*p_Firmicutes;c_Bacilli;o_Lactobacillales;f_Lactobacillaceae;g_Lactobacillus;s_*			**8.27**	6.60E-26
549991	*p_Firmicutes;c_Bacilli;o_Lactobacillales;f_Lactobacillaceae;g_Lactobacillus;s_*			**7.97**	1.69E-26
538223	*p_Firmicutes;c_Bacilli;o_Lactobacillales;f_Lactobacillaceae;g_Lactobacillus;s_*			**7.12**	1.54E-28
302975	*p_Firmicutes;c_Bacilli;o_Lactobacillales;f_Lactobacillaceae;g_Lactobacillus;s_*			**6.60**	2.95E-16
146935	*p_Firmicutes;c_Bacilli;o_Lactobacillales;f_Lactobacillaceae;g_Lactobacillus;s_*			**5.79**	3.98E-12
589114	*p_Firmicutes;c_Bacilli;o_Lactobacillales;f_Lactobacillaceae;g_Lactobacillus;s_*			**4.95**	8.54E-09
806179	*p_Firmicutes;c_Bacilli;o_Lactobacillales;f_Lactobacillaceae;g_Lactobacillus;s_*			**4.78**	8.73E-08
584571	*p_Firmicutes;c_Bacilli;o_Lactobacillales;f_Lactobacillaceae;g_Lactobacillus;s_*			**4.62**	8.70E-08
255367	*p_Firmicutes;c_Bacilli;o_Lactobacillales;f_Lactobacillaceae;g_Lactobacillus;s_*			**4.58**	1.24E-07
456393	*p_Firmicutes;c_Bacilli;o_Lactobacillales;f_Lactobacillaceae;g_Lactobacillus;s_*			**3.95**	6.28E-06
749329	*p_Firmicutes;c_Bacilli;o_Lactobacillales;f_Lactobacillaceae;g_Lactobacillus;s_*			**3.65**	6.17E-05

809106	*p_Firmicutes;c_Bacilli;o_Lactobacillales;f_Lactobacillaceae;g_Lactobacillus;s_*	**3.49**	7.73E-05
823948	*p_Firmicutes;c_Bacilli;o_Lactobacillales;f_Lactobacillaceae;g_Lactobacillus;s_*	**3.32**	1.69E-04
819507	*p_Firmicutes;c_Bacilli;o_Lactobacillales;f_Lactobacillaceae;g_Lactobacillus;s_*	**3.25**	2.73E-04
463361	*p_Firmicutes;c_Bacilli;o_Lactobacillales;f_Lactobacillaceae;g_Lactobacillus;s_*	**3.20**	3.15E-04
821597	*p_Firmicutes;c_Bacilli;o_Lactobacillales;f_Lactobacillaceae;g_Lactobacillus;s_*	**3.01**	8.82E-04
537734	*p_Firmicutes;c_Bacilli;o_Lactobacillales;f_Lactobacillaceae;g_Lactobacillus;s_*	**2.95**	1.15E-03
586093	*p_Firmicutes;c_Bacilli;o_Lactobacillales;f_Lactobacillaceae;g_Lactobacillus;s_*	**2.92**	1.32E-03
851794	*p_Firmicutes;c_Bacilli;o_Lactobacillales;f_Lactobacillaceae;g_Lactobacillus;s_*	**2.59**	4.71E-04
131702	*p_Firmicutes;c_Bacilli;o_Lactobacillales;f_Lactobacillaceae;g_Lactobacillus;s_*	**2.29**	1.70E-02
164664	*p_Firmicutes;c_Bacilli;o_Lactobacillales;f_Lactobacillaceae;g_Lactobacillus;s_*	**-2.23**	8.90E-03
452823	*p_Firmicutes;c_Bacilli;o_Lactobacillales;f_Lactobacillaceae;g_Lactobacillus;s_*	**-2.48**	2.99E-03
259111	*p_Firmicutes;c_Bacilli;o_Lactobacillales;f_Lactobacillaceae;g_Lactobacillus;s_*	**-2.56**	5.79E-03
333178	*p_Firmicutes;c_Bacilli;o_Lactobacillales;f_Lactobacillaceae;g_Lactobacillus;s_*	**-2.92**	5.59E-06
463794	*p_Firmicutes;c_Bacilli;o_Lactobacillales;f_Lactobacillaceae;g_Lactobacillus;s_*	**-2.94**	1.53E-05
182758	*p_Firmicutes;c_Bacilli;o_Lactobacillales;f_Lactobacillaceae;g_Lactobacillus;s_*	**-3.24**	3.21E-06
**807795**	***p_Firmicutes;c_Bacilli;o_Lactobacillales;f_Lactobacillaceae;g_Lactobacillus;s_helveticus*** **(strain MIMLh5)**	**8.40**	**1.80E-36**
276305	*p_Firmicutes;c_Clostridia;o_Clostridiales;f_;g_;s_*	**4.87**	9.04E-06
276770	*p_Firmicutes;c_Clostridia;o_Clostridiales;f_;g_;s_*	**4.85**	1.38E-05
277068	*p_Firmicutes;c_Clostridia;o_Clostridiales;f_;g_;s_*	**4.29**	1.01E-04
351593	*p_Firmicutes;c_Clostridia;o_Clostridiales;f_;g_;s_*	**3.57**	6.69E-04
275366	*p_Firmicutes;c_Clostridia;o_Clostridiales;f_;g_;s_*	**3.52**	1.92E-03
353657	*p_Firmicutes;c_Clostridia;o_Clostridiales;f_;g_;s_*	**3.38**	1.20E-03
4480555	*p_Firmicutes;c_Clostridia;o_Clostridiales;f_;g_;s_*	**2.99**	8.30E-03
675863	*p_Firmicutes;c_Clostridia;o_Clostridiales;f_;g_;s_*	**2.91**	9.14E-03
833816	*p_Firmicutes;c_Clostridia;o_Clostridiales;f_;g_;s_*	**2.87**	9.42E-03
173739	*p_Firmicutes;c_Clostridia;o_Clostridiales;f_;g_;s_*	**2.47**	4.45E-02
273479	*p_Firmicutes;c_Clostridia;o_Clostridiales;f_;g_;s_*	**2.41**	1.85E-02
314810	*p_Firmicutes;c_Clostridia;o_Clostridiales;f_;g_;s_*	**2.29**	3.77E-02
4455677	*p_Firmicutes;c_Clostridia;o_Clostridiales;f_;g_;s_*	**-2.17**	2.52E-02
665703	*p_Firmicutes;c_Clostridia;o_Clostridiales;f_;g_;s_*	**-2.54**	2.63E-02
4402077	*p_Firmicutes;c_Clostridia;o_Clostridiales;f_;g_;s_*	**-2.74**	5.19E-03
261097	*p_Firmicutes;c_Clostridia;o_Clostridiales;f_;g_;s_*	**-4.12**	2.43E-04
4365109	*p_Firmicutes;c_Clostridia;o_Clostridiales;f_;g_;s_*	**-4.59**	1.04E-05
449353	*p_Firmicutes;c_Clostridia;o_Clostridiales;f_Dehalobacteriaceae;g_Dehalobacterium;s_*	**2.69**	9.55E-03
838200	*p_Firmicutes;c_Clostridia;o_Clostridiales;f_Lachnospiraceae;g_;s_*	**3.11**	7.15E-03
351881	*p_Firmicutes;c_Clostridia;o_Clostridiales;f_Lachnospiraceae;g_;s_*	**2.90**	2.96E-03
450047	*p_Firmicutes;c_Clostridia;o_Clostridiales;f_Lachnospiraceae;g_;s_*	**2.58**	1.30E-02
355822	*p_Firmicutes;c_Clostridia;o_Clostridiales;f_Lachnospiraceae;g_;s_*	**1.99**	4.12E-02
336691	*p_Firmicutes;c_Clostridia;o_Clostridiales;f_Ruminococcaceae;g_;s_*	**2.32**	2.56E-02
1111350	*p_Firmicutes;c_Clostridia;o_Clostridiales;f_Ruminococcaceae;g_;s_*	**1.97**	3.15E-02
163153	*p_Firmicutes;c_Clostridia;o_Clostridiales;f_Ruminococcaceae;g_Oscillospira;s_*	**3.45**	1.89E-03
327808	*p_Firmicutes;c_Clostridia;o_Clostridiales;f_Ruminococcaceae;g_Oscillospira;s_*	**2.94**	9.54E-04
443620	*p_Firmicutes;c_Clostridia;o_Clostridiales;f_Ruminococcaceae;g_Oscillospira;s_*	**2.26**	3.51E-02
272188	*p_Firmicutes;c_Clostridia;o_Clostridiales;f_Ruminococcaceae;g_Oscillospira;s_*	**2.25**	2.12E-02
585073	*p_Firmicutes;c_Clostridia;o_Clostridiales;f_Ruminococcaceae;g_Oscillospira;s_*	**2.13**	4.34E-02
350404	*p_Firmicutes;c_Clostridia;o_Clostridiales;f_Ruminococcaceae;g_Oscillospira;s_*	**2.12**	3.23E-02
317633	*p_Firmicutes;c_Clostridia;o_Clostridiales;f_Ruminococcaceae;g_Oscillospira;s_*	**2.05**	3.97E-02
387615	*p_Firmicutes;c_Clostridia;o_Clostridiales;f_Ruminococcaceae;g_Oscillospira;s_*	**1.94**	4.79E-02
262101	*p_Firmicutes;c_Erysipelotrichi;o_Erysipelotrichales;f_Erysipelotrichaceae;g_Allobaculum;s_*	**3.26**	3.38E-04
437137	*p_Proteobacteria;c_Betaproteobacteria;o_Burkholderiales;f_Alcaligenaceae;g_Sutterella;s_*	**2.50**	3.40E-03
169901	*p_TM7;c_TM7-3;o_CW040;f_F16;g_;s_*	**-2.30**	1.97E-02

min **max** **-5.7** **+9.4**

C

Mice gavaged with *Lacticaseibacillus paracasei* DG

Ileum

OTU nr.	Taxonomy	Mean normalized abundance PBS	Mean normalized abundance DG	log2 Fold Change	padj
168047	*p_Actinobacteria;c_Coriobacteriia;o_Coriobacteriales;f_Coriobacteriaceae;g_Adlercreutzia;s_*			**-3.56**	4.45E-03
339013	*p_Bacteroidetes;c_Bacteroidia;o_Bacteroidales;f_Bacteroidaceae;g_Bacteroides;s_*			**-2.56**	4.67E-02
276149	*p_Bacteroidetes;c_Bacteroidia;o_Bacteroidales;f_Porphyromonadaceae;g_Parabacteroides;s_*			**2.50**	3.13E-02
276218	*p_Bacteroidetes;c_Bacteroidia;o_Bacteroidales;f_S24-7;g_;s_*			**4.35**	4.59E-04
192283	*p_Bacteroidetes;c_Bacteroidia;o_Bacteroidales;f_S24-7;g_;s_*			**4.38**	4.59E-04
356226	*p_Bacteroidetes;c_Bacteroidia;o_Bacteroidales;f_S24-7;g_;s_*			**-3.65**	1.59E-03
353012	*p_Bacteroidetes;c_Bacteroidia;o_Bacteroidales;f_S24-7;g_;s_*			**-4.17**	2.42E-03
342962	*p_Bacteroidetes;c_Bacteroidia;o_Bacteroidales;f_S24-7;g_;s_*			**-3.41**	5.24E-03
263111	*p_Bacteroidetes;c_Bacteroidia;o_Bacteroidales;f_S24-7;g_;s_*			**-2.91**	8.21E-03
331043	*p_Bacteroidetes;c_Bacteroidia;o_Bacteroidales;f_S24-7;g_;s_*			**-2.54**	3.13E-02
233587	*p_Bacteroidetes;c_Bacteroidia;o_Bacteroidales;f_S24-7;g_;s_*			**-2.69**	3.66E-02
339905	*p_Bacteroidetes;c_Bacteroidia;o_Bacteroidales;f_S24-7;g_;s_*			**-2.75**	4.78E-02
3231096	*p_Bacteroidetes;c_Bacteroidia;o_Bacteroidales;f_S24-7;g_;s_*			**-2.25**	4.85E-02
452823	*p_Firmicutes;c_Bacilli;o_Lactobacillales;f_Lactobacillaceae;g_Lactobacillus;s_*			**-3.28**	2.68E-03
463794	*p_Firmicutes;c_Bacilli;o_Lactobacillales;f_Lactobacillaceae;g_Lactobacillus;s_*			**-3.26**	4.60E-03
182758	*p_Firmicutes;c_Bacilli;o_Lactobacillales;f_Lactobacillaceae;g_Lactobacillus;s_*			**-3.10**	4.92E-03
259111	*p_Firmicutes;c_Bacilli;o_Lactobacillales;f_Lactobacillaceae;g_Lactobacillus;s_*			**-2.61**	1.58E-02
333178	*p_Firmicutes;c_Bacilli;o_Lactobacillales;f_Lactobacillaceae;g_Lactobacillus;s_*			**-2.89**	1.70E-02
289918	*p_Firmicutes;c_Bacilli;o_Lactobacillales;f_Lactobacillaceae;g_Lactobacillus;s_*			**-2.36**	3.13E-02
581474	*p_Firmicutes;c_Bacilli;o_Lactobacillales;f_Lactobacillaceae;g_Lactobacillus;s_*			**-2.49**	3.56E-02
259150	*p_Firmicutes;c_Bacilli;o_Lactobacillales;f_Lactobacillaceae;g_Lactobacillus;s_*			**-2.42**	3.72E-02
350242	*p_Firmicutes;c_Bacilli;o_Lactobacillales;f_Lactobacillaceae;g_Lactobacillus;s_*			**-2.26**	3.99E-02
164187	*p_Firmicutes;c_Bacilli;o_Lactobacillales;f_Lactobacillaceae;g_Lactobacillus;s_*			**-1.94**	4.85E-02
549756	*p_Firmicutes;c_Bacilli;o_Lactobacillales;f_Lactobacillaceae;g_Lactobacillus;s_*			**-2.28**	4.85E-02
354905	*p_Firmicutes;c_Bacilli;o_Lactobacillales;f_Lactobacillaceae;g_Lactobacillus;s_*			**-2.28**	4.85E-02
164664	*p_Firmicutes;c_Bacilli;o_Lactobacillales;f_Lactobacillaceae;g_Lactobacillus;s_*			**-2.08**	4.85E-02
187233	*p_Firmicutes;c_Bacilli;o_Lactobacillales;f_Lactobacillaceae;g_Lactobacillus;s_*			**-1.90**	4.85E-02
**751601**	***p_Firmicutes;c_Bacilli;o_Lactobacillales;f_Lactobacillaceae;g_Lactobacillus;s_zeae/paracasei*** **(strain DG)**			**6.33**	**NA**
214919	*p_Firmicutes;c_Bacilli;o_Turicibacterales;f_Turicibacteraceae;g_Turicibacter;s_*			**-2.89**	4.86E-02
4402077	*p_Firmicutes;c_Clostridia;o_Clostridiales;f_;g_;s_*			**-2.16**	4.86E-02
340189	*p_Firmicutes;c_Clostridia;o_Clostridiales;f_Lachnospiraceae;g_;s_*			**-2.49**	4.85E-02
330116	*p_Firmicutes;c_Clostridia;o_Clostridiales;f_Lachnospiraceae;g_[Ruminococcus];s_gnavus*			**-4.33**	2.42E-03
1918929	*p_Proteobacteria;c_Alphaproteobacteria;o_Rickettsiales;f_mitochondria;g_Zea;s_luxurians*			**-2.17**	4.85E-02
768553	*p_Proteobacteria;c_Gammaproteobacteria;o_Enterobacteriales;f_Enterobacteriaceae;g_Proteus;s_*			**-3.77**	4.60E-03

Cecum

OTU nr.	Taxonomy	Mean normalized abundance PBS	Mean normalized abundance DG	log2 Fold Change	padj
168047	*p_Actinobacteria;c_Coriobacteriia;o_Coriobacteriales;f_Coriobacteriaceae;g_Adlercreutzia;s_*			**-2.82**	3.70E-04
193279	*p_Actinobacteria;c_Coriobacteriia;o_Coriobacteriales;f_Coriobacteriaceae;g_Adlercreutzia;s_*			**-1.77**	3.94E-02
583117	*p_Bacteroidetes;c_Bacteroidia;o_Bacteroidales;f_Bacteroidaceae;g_Bacteroides;s_*			**1.44**	3.94E-02
192283	*p_Bacteroidetes;c_Bacteroidia;o_Bacteroidales;f_S24-7;g_;s_*			**8.28**	6.48E-37
276218	*p_Bacteroidetes;c_Bacteroidia;o_Bacteroidales;f_S24-7;g_;s_*			**8.23**	1.17E-32
272209	*p_Bacteroidetes;c_Bacteroidia;o_Bacteroidales;f_S24-7;g_;s_*			**8.69**	6.09E-32
267046	*p_Bacteroidetes;c_Bacteroidia;o_Bacteroidales;f_S24-7;g_;s_*			**6.20**	5.96E-15
339156	*p_Bacteroidetes;c_Bacteroidia;o_Bacteroidales;f_S24-7;g_;s_*			**5.54**	6.32E-14
270158	*p_Bacteroidetes;c_Bacteroidia;o_Bacteroidales;f_S24-7;g_;s_*			**5.43**	2.25E-11
191994	*p_Bacteroidetes;c_Bacteroidia;o_Bacteroidales;f_S24-7;g_;s_*			**4.54**	1.59E-08
208409	*p_Bacteroidetes;c_Bacteroidia;o_Bacteroidales;f_S24-7;g_;s_*			**4.53**	2.89E-08
331772	*p_Bacteroidetes;c_Bacteroidia;o_Bacteroidales;f_S24-7;g_;s_*			**2.81**	6.07E-06
217100	*p_Bacteroidetes;c_Bacteroidia;o_Bacteroidales;f_S24-7;g_;s_*			**2.81**	5.06E-05
313499	*p_Bacteroidetes;c_Bacteroidia;o_Bacteroidales;f_S24-7;g_;s_*			**2.20**	3.21E-04
334040	*p_Bacteroidetes;c_Bacteroidia;o_Bacteroidales;f_S24-7;g_;s_*			**2.05**	3.24E-04
353453	*p_Bacteroidetes;c_Bacteroidia;o_Bacteroidales;f_S24-7;g_;s_*			**2.02**	4.08E-04
384555	*p_Bacteroidetes;c_Bacteroidia;o_Bacteroidales;f_S24-7;g_;s_*			**2.88**	5.27E-04
319525	*p_Bacteroidetes;c_Bacteroidia;o_Bacteroidales;f_S24-7;g_;s_*			**1.96**	2.35E-03
829302	*p_Bacteroidetes;c_Bacteroidia;o_Bacteroidales;f_S24-7;g_;s_*			**2.90**	2.66E-03
416078	*p_Bacteroidetes;c_Bacteroidia;o_Bacteroidales;f_S24-7;g_;s_*			**2.02**	3.17E-03
189730	*p_Bacteroidetes;c_Bacteroidia;o_Bacteroidales;f_S24-7;g_;s_*			**2.29**	4.24E-03
192494	*p_Bacteroidetes;c_Bacteroidia;o_Bacteroidales;f_S24-7;g_;s_*			**1.93**	4.46E-03
277120	*p_Bacteroidetes;c_Bacteroidia;o_Bacteroidales;f_S24-7;g_;s_*			**1.64**	6.46E-03
215495	*p_Bacteroidetes;c_Bacteroidia;o_Bacteroidales;f_S24-7;g_;s_*			**1.80**	1.55E-02
330772	*p_Bacteroidetes;c_Bacteroidia;o_Bacteroidales;f_S24-7;g_;s_*			**1.72**	1.57E-02
264657	*p_Bacteroidetes;c_Bacteroidia;o_Bacteroidales;f_S24-7;g_;s_*			**1.70**	2.41E-02
261350	*p_Bacteroidetes;c_Bacteroidia;o_Bacteroidales;f_S24-7;g_;s_*			**1.54**	2.44E-02
196385	*p_Bacteroidetes;c_Bacteroidia;o_Bacteroidales;f_S24-7;g_;s_*			**1.57**	2.56E-02
275339	*p_Bacteroidetes;c_Bacteroidia;o_Bacteroidales;f_S24-7;g_;s_*			**1.68**	2.72E-02
389282	*p_Bacteroidetes;c_Bacteroidia;o_Bacteroidales;f_S24-7;g_;s_*			**1.54**	2.78E-02
2435303	*p_Bacteroidetes;c_Bacteroidia;o_Bacteroidales;f_S24-7;g_;s_*			**1.54**	2.98E-02
185550	*p_Bacteroidetes;c_Bacteroidia;o_Bacteroidales;f_S24-7;g_;s_*			**1.52**	3.39E-02
270984	*p_Bacteroidetes;c_Bacteroidia;o_Bacteroidales;f_S24-7;g_;s_*			**1.49**	3.66E-02
247715	*p_Bacteroidetes;c_Bacteroidia;o_Bacteroidales;f_S24-7;g_;s_*			**1.83**	3.94E-02
4405128	*p_Cyanobacteria;c_4C0d-2;o_YS2;f_;g_;s_*			**2.66**	6.61E-04

389067	*p_Firmicutes;c_Bacilli;o_Lactobacillales;f_Lactobacillaceae;g_Lactobacillus;s_*			**3.34**	4.00E-05
807795	*p_Firmicutes;c_Bacilli;o_Lactobacillales;f_Lactobacillaceae;g_Lactobacillus;s_*			**2.06**	1.36E-03
851794	*p_Firmicutes;c_Bacilli;o_Lactobacillales;f_Lactobacillaceae;g_Lactobacillus;s_*			**2.05**	9.01E-03
**751601**	***p_Firmicutes;c_Bacilli;o_Lactobacillales;f_Lactobacillaceae;g_Lactobacillus;s_zeae/paracasei* (strain DG)**			**9.37**	**1.27E-47**
518033	*p_Firmicutes;c_Bacilli;o_Lactobacillales;f_Lactobacillaceae;g_Lactobacillus;s_zeae*			**6.82**	1.99E-21
770290	*p_Firmicutes;c_Bacilli;o_Lactobacillales;f_Lactobacillaceae;g_Lactobacillus;s_zeae*			**3.91**	1.31E-06
266330	*p_Firmicutes;c_Bacilli;o_Lactobacillales;f_Lactobacillaceae;g_Lactobacillus;s_zeae*			**2.44**	5.29E-03
214919	*p_Firmicutes;c_Bacilli;o_Turicibacterales;f_Turicibacteraceae;g_Turicibacter;s_*			**-2.45**	3.94E-02
276305	*p_Firmicutes;c_Clostridia;o_Clostridiales;f_;g_;s_*			**6.69**	1.24E-19
354501	*p_Firmicutes;c_Clostridia;o_Clostridiales;f_;g_;s_*			**5.26**	2.39E-09
675863	*p_Firmicutes;c_Clostridia;o_Clostridiales;f_;g_;s_*			**4.46**	1.48E-06
199532	*p_Firmicutes;c_Clostridia;o_Clostridiales;f_;g_;s_*			**-4.62**	1.57E-06
352049	*p_Firmicutes;c_Clostridia;o_Clostridiales;f_;g_;s_*			**3.92**	2.09E-05
185334	*p_Firmicutes;c_Clostridia;o_Clostridiales;f_;g_;s_*			**3.62**	3.22E-05
194787	*p_Firmicutes;c_Clostridia;o_Clostridiales;f_;g_;s_*			**3.81**	3.32E-05
324865	*p_Firmicutes;c_Clostridia;o_Clostridiales;f_;g_;s_*			**3.90**	4.00E-05
277068	*p_Firmicutes;c_Clostridia;o_Clostridiales;f_;g_;s_*			**4.04**	4.00E-05
353657	*p_Firmicutes;c_Clostridia;o_Clostridiales;f_;g_;s_*			**3.22**	5.20E-05
276674	*p_Firmicutes;c_Clostridia;o_Clostridiales;f_;g_;s_*			**3.20**	5.09E-04
329712	*p_Firmicutes;c_Clostridia;o_Clostridiales;f_;g_;s_*			**2.80**	5.53E-04
261097	*p_Firmicutes;c_Clostridia;o_Clostridiales;f_;g_;s_*			**-3.95**	5.70E-04
340706	*p_Firmicutes;c_Clostridia;o_Clostridiales;f_;g_;s_*			**3.13**	5.82E-04
132338	*p_Firmicutes;c_Clostridia;o_Clostridiales;f_;g_;s_*			**2.93**	9.99E-04
330460	*p_Firmicutes;c_Clostridia;o_Clostridiales;f_;g_;s_*			**3.02**	1.24E-03
269035	*p_Firmicutes;c_Clostridia;o_Clostridiales;f_;g_;s_*			**3.00**	1.36E-03
199215	*p_Firmicutes;c_Clostridia;o_Clostridiales;f_;g_;s_*			**2.49**	1.58E-03
1107461	*p_Firmicutes;c_Clostridia;o_Clostridiales;f_;g_;s_*			**2.57**	2.31E-03
198271	*p_Firmicutes;c_Clostridia;o_Clostridiales;f_;g_;s_*			**-3.17**	2.66E-03
351593	*p_Firmicutes;c_Clostridia;o_Clostridiales;f_;g_;s_*			**2.58**	3.39E-03
831409	*p_Firmicutes;c_Clostridia;o_Clostridiales;f_;g_;s_*			**2.73**	3.41E-03
333426	*p_Firmicutes;c_Clostridia;o_Clostridiales;f_;g_;s_*			**3.32**	3.47E-03
4399086	*p_Firmicutes;c_Clostridia;o_Clostridiales;f_;g_;s_*			**-3.33**	3.47E-03
212732	*p_Firmicutes;c_Clostridia;o_Clostridiales;f_;g_;s_*			**-2.71**	5.46E-03
354662	*p_Firmicutes;c_Clostridia;o_Clostridiales;f_;g_;s_*			**2.19**	5.46E-03
271431	*p_Firmicutes;c_Clostridia;o_Clostridiales;f_;g_;s_*			**2.38**	5.46E-03
276770	*p_Firmicutes;c_Clostridia;o_Clostridiales;f_;g_;s_*			**2.60**	7.97E-03
275139	*p_Firmicutes;c_Clostridia;o_Clostridiales;f_;g_;s_*			**2.57**	1.09E-02
182079	*p_Firmicutes;c_Clostridia;o_Clostridiales;f_;g_;s_*			**-2.39**	1.55E-02
833795	*p_Firmicutes;c_Clostridia;o_Clostridiales;f_;g_;s_*			**2.40**	1.93E-02
183838	*p_Firmicutes;c_Clostridia;o_Clostridiales;f_;g_;s_*			**2.10**	2.51E-02
273479	*p_Firmicutes;c_Clostridia;o_Clostridiales;f_;g_;s_*			**1.93**	2.89E-02
827689	*p_Firmicutes;c_Clostridia;o_Clostridiales;f_;g_;s_*			**1.87**	3.39E-02
355097	*p_Firmicutes;c_Clostridia;o_Clostridiales;f_;g_;s_*			**1.78**	3.45E-02
275180	*p_Firmicutes;c_Clostridia;o_Clostridiales;f_;g_;s_*			**1.93**	3.45E-02
833816	*p_Firmicutes;c_Clostridia;o_Clostridiales;f_;g_;s_*			**2.06**	3.46E-02
318162	*p_Firmicutes;c_Clostridia;o_Clostridiales;f_;g_;s_*			**1.47**	3.53E-02
268063	*p_Firmicutes;c_Clostridia;o_Clostridiales;f_;g_;s_*			**1.94**	3.75E-02
343264	*p_Firmicutes;c_Clostridia;o_Clostridiales;f_;g_;s_*			**1.99**	4.12E-02
4355739	*p_Firmicutes;c_Clostridia;o_Clostridiales;f_;g_;s_*			**1.78**	4.19E-02
581463	*p_Firmicutes;c_Clostridia;o_Clostridiales;f_Clostridiaceae;g_Clostridium;s_*			**2.49**	7.30E-03
265018	*p_Firmicutes;c_Clostridia;o_Clostridiales;f_Lachnospiraceae;g_;s_*			**6.14**	5.36E-15
450047	*p_Firmicutes;c_Clostridia;o_Clostridiales;f_Lachnospiraceae;g_;s_*			**4.15**	1.57E-06
4471525	*p_Firmicutes;c_Clostridia;o_Clostridiales;f_Lachnospiraceae;g_;s_*			**3.10**	5.07E-04
838200	*p_Firmicutes;c_Clostridia;o_Clostridiales;f_Lachnospiraceae;g_;s_*			**3.15**	8.27E-04
351881	*p_Firmicutes;c_Clostridia;o_Clostridiales;f_Lachnospiraceae;g_;s_*			**2.42**	7.10E-03
340189	*p_Firmicutes;c_Clostridia;o_Clostridiales;f_Lachnospiraceae;g_;s_*			**-2.02**	1.24E-02
310760	*p_Firmicutes;c_Clostridia;o_Clostridiales;f_Lachnospiraceae;g_;s_*			**2.42**	1.41E-02
269949	*p_Firmicutes;c_Clostridia;o_Clostridiales;f_Lachnospiraceae;g_;s_*			**2.08**	1.98E-02
345709	*p_Firmicutes;c_Clostridia;o_Clostridiales;f_Lachnospiraceae;g_;s_*			**2.11**	3.41E-02
258169	*p_Firmicutes;c_Clostridia;o_Clostridiales;f_Lachnospiraceae;g_;s_*			**-1.80**	3.45E-02
267298	*p_Firmicutes;c_Clostridia;o_Clostridiales;f_Lachnospiraceae;g_[Ruminococcus];s_gnavus*			**1.91**	3.39E-02
295370	*p_Firmicutes;c_Clostridia;o_Clostridiales;f_Lachnospiraceae;g_[Ruminococcus];s_gnavus*			**-2.52**	3.41E-02
301872	*p_Firmicutes;c_Clostridia;o_Clostridiales;f_Lachnospiraceae;g_[Ruminococcus];s_gnavus*			**-2.68**	3.86E-02
653533	*p_Firmicutes;c_Clostridia;o_Clostridiales;f_Lachnospiraceae;g_Coprococcus;s_*			**2.01**	3.41E-02
338926	*p_Firmicutes;c_Clostridia;o_Clostridiales;f_Lachnospiraceae;g_Dorea;s_*			**-1.90**	3.39E-02
531436	*p_Firmicutes;c_Clostridia;o_Clostridiales;f_Lachnospiraceae;g_Roseburia;s_*			**2.02**	3.45E-02
318370	*p_Firmicutes;c_Clostridia;o_Clostridiales;f_Ruminococcaceae;g_;s_*			**2.60**	9.89E-05
583134	*p_Firmicutes;c_Clostridia;o_Clostridiales;f_Ruminococcaceae;g_;s_*			**2.44**	2.71E-03
176582	*p_Firmicutes;c_Clostridia;o_Clostridiales;f_Ruminococcaceae;g_;s_*			**2.17**	7.03E-03
331737	*p_Firmicutes;c_Clostridia;o_Clostridiales;f_Ruminococcaceae;g_;s_*			**2.10**	7.97E-03
1111350	*p_Firmicutes;c_Clostridia;o_Clostridiales;f_Ruminococcaceae;g_;s_*			**2.03**	1.48E-02
4338227	*p_Firmicutes;c_Clostridia;o_Clostridiales;f_Ruminococcaceae;g_;s_*			**2.22**	1.52E-02
163153	*p_Firmicutes;c_Clostridia;o_Clostridiales;f_Ruminococcaceae;g_Oscillospira;s_*			**4.43**	1.15E-06
443620	*p_Firmicutes;c_Clostridia;o_Clostridiales;f_Ruminococcaceae;g_Oscillospira;s_*			**2.17**	5.09E-03
350404	*p_Firmicutes;c_Clostridia;o_Clostridiales;f_Ruminococcaceae;g_Oscillospira;s_*			**1.35**	4.81E-02
167420	*p_Firmicutes;c_Erysipelotrichi;o_Erysipelotrichales;f_Erysipelotrichaceae;g_;s_*			**2.38**	1.44E-02
437137	*p_Proteobacteria;c_Betaproteobacteria;o_Burkholderiales;f_Alcaligenaceae;g_Sutterella;s_*			**2.85**	5.07E-04
363731	*p_Verrucomicrobia;c_Verrucomicrobiae;o_Verrucomicrobiales;f_Verrucomicrobiaceae;g_Akkermansia;s_muciniphila*			**-3.95**	1.49E-05

Colon

OTU nr.	Taxonomy	Mean normalized abundance		log2 Fold Change	padj
		PBS	DG		
276151	*p_Actinobacteria;c_Coriobacteriia;o_Coriobacteriales;f_Coriobacteriaceae;g_Adlercreutzia;s_*			**1.95**	1.41E-02

275974	*p_Actinobacteria;c_Coriobacteriia;o_Coriobacteriales;f_Coriobacteriaceae;g_Adlercreutzia;s_*	**1.95**	2.78E-02
168047	*p_Actinobacteria;c_Coriobacteriia;o_Coriobacteriales;f_Coriobacteriaceae;g_Adlercreutzia;s_*	**-1.99**	3.19E-02
326662	*p_Bacteroidetes;c_Bacteroidia;o_Bacteroidales;f_Bacteroidaceae;g_Bacteroides;s_*	**2.18**	1.73E-04
276172	*p_Bacteroidetes;c_Bacteroidia;o_Bacteroidales;f_Rikenellaceae;g_;s_*	**-1.92**	4.23E-02
272209	*p_Bacteroidetes;c_Bacteroidia;o_Bacteroidales;f_S24-7;g_;s_*	**9.26**	4.20E-40
192283	*p_Bacteroidetes;c_Bacteroidia;o_Bacteroidales;f_S24-7;g_;s_*	**8.39**	1.10E-37
276218	*p_Bacteroidetes;c_Bacteroidia;o_Bacteroidales;f_S24-7;g_;s_*	**7.73**	4.65E-23
267046	*p_Bacteroidetes;c_Bacteroidia;o_Bacteroidales;f_S24-7;g_;s_*	**6.73**	3.64E-18
339156	*p_Bacteroidetes;c_Bacteroidia;o_Bacteroidales;f_S24-7;g_;s_*	**6.28**	1.64E-16
331772	*p_Bacteroidetes;c_Bacteroidia;o_Bacteroidales;f_S24-7;g_;s_*	**4.56**	2.68E-12
270158	*p_Bacteroidetes;c_Bacteroidia;o_Bacteroidales;f_S24-7;g_;s_*	**5.64**	5.80E-10
191994	*p_Bacteroidetes;c_Bacteroidia;o_Bacteroidales;f_S24-7;g_;s_*	**4.59**	2.90E-08
217100	*p_Bacteroidetes;c_Bacteroidia;o_Bacteroidales;f_S24-7;g_;s_*	**3.87**	5.59E-07
215495	*p_Bacteroidetes;c_Bacteroidia;o_Bacteroidales;f_S24-7;g_;s_*	**3.59**	1.98E-06
353453	*p_Bacteroidetes;c_Bacteroidia;o_Bacteroidales;f_S24-7;g_;s_*	**2.49**	1.01E-05
334040	*p_Bacteroidetes;c_Bacteroidia;o_Bacteroidales;f_S24-7;g_;s_*	**2.78**	1.90E-05
208409	*p_Bacteroidetes;c_Bacteroidia;o_Bacteroidales;f_S24-7;g_;s_*	**4.05**	4.01E-05
829302	*p_Bacteroidetes;c_Bacteroidia;o_Bacteroidales;f_S24-7;g_;s_*	**3.56**	6.59E-04
313499	*p_Bacteroidetes;c_Bacteroidia;o_Bacteroidales;f_S24-7;g_;s_*	**2.40**	6.98E-04
319525	*p_Bacteroidetes;c_Bacteroidia;o_Bacteroidales;f_S24-7;g_;s_*	**2.28**	7.28E-04
264734	*p_Bacteroidetes;c_Bacteroidia;o_Bacteroidales;f_S24-7;g_;s_*	**2.30**	8.55E-04
192494	*p_Bacteroidetes;c_Bacteroidia;o_Bacteroidales;f_S24-7;g_;s_*	**2.25**	1.04E-03
384555	*p_Bacteroidetes;c_Bacteroidia;o_Bacteroidales;f_S24-7;g_;s_*	**3.22**	1.16E-03
228730	*p_Bacteroidetes;c_Bacteroidia;o_Bacteroidales;f_S24-7;g_;s_*	**2.00**	1.81E-03
263479	*p_Bacteroidetes;c_Bacteroidia;o_Bacteroidales;f_S24-7;g_;s_*	**1.76**	2.32E-03
199118	*p_Bacteroidetes;c_Bacteroidia;o_Bacteroidales;f_S24-7;g_;s_*	**2.49**	3.83E-03
389282	*p_Bacteroidetes;c_Bacteroidia;o_Bacteroidales;f_S24-7;g_;s_*	**2.13**	3.83E-03
258485	*p_Bacteroidetes;c_Bacteroidia;o_Bacteroidales;f_S24-7;g_;s_*	**1.72**	4.33E-03
189730	*p_Bacteroidetes;c_Bacteroidia;o_Bacteroidales;f_S24-7;g_;s_*	**2.63**	5.46E-03
277120	*p_Bacteroidetes;c_Bacteroidia;o_Bacteroidales;f_S24-7;g_;s_*	**1.57**	6.32E-03
211595	*p_Bacteroidetes;c_Bacteroidia;o_Bacteroidales;f_S24-7;g_;s_*	**2.39**	1.16E-02
196385	*p_Bacteroidetes;c_Bacteroidia;o_Bacteroidales;f_S24-7;g_;s_*	**1.50**	1.23E-02
330772	*p_Bacteroidetes;c_Bacteroidia;o_Bacteroidales;f_S24-7;g_;s_*	**1.85**	1.26E-02
264657	*p_Bacteroidetes;c_Bacteroidia;o_Bacteroidales;f_S24-7;g_;s_*	**1.90**	1.26E-02
273164	*p_Bacteroidetes;c_Bacteroidia;o_Bacteroidales;f_S24-7;g_;s_*	**2.38**	1.42E-02
372368	*p_Bacteroidetes;c_Bacteroidia;o_Bacteroidales;f_S24-7;g_;s_*	**1.63**	1.42E-02
348038	*p_Bacteroidetes;c_Bacteroidia;o_Bacteroidales;f_S24-7;g_;s_*	**1.33**	1.42E-02
206456	*p_Bacteroidetes;c_Bacteroidia;o_Bacteroidales;f_S24-7;g_;s_*	**2.02**	1.42E-02
211876	*p_Bacteroidetes;c_Bacteroidia;o_Bacteroidales;f_S24-7;g_;s_*	**2.30**	1.42E-02
178068	*p_Bacteroidetes;c_Bacteroidia;o_Bacteroidales;f_S24-7;g_;s_*	**1.75**	1.73E-02
266860	*p_Bacteroidetes;c_Bacteroidia;o_Bacteroidales;f_S24-7;g_;s_*	**1.59**	1.79E-02
348088	*p_Bacteroidetes;c_Bacteroidia;o_Bacteroidales;f_S24-7;g_;s_*	**1.68**	1.81E-02
175272	*p_Bacteroidetes;c_Bacteroidia;o_Bacteroidales;f_S24-7;g_;s_*	**2.29**	1.84E-02
416078	*p_Bacteroidetes;c_Bacteroidia;o_Bacteroidales;f_S24-7;g_;s_*	**1.50**	1.87E-02
189327	*p_Bacteroidetes;c_Bacteroidia;o_Bacteroidales;f_S24-7;g_;s_*	**1.94**	1.94E-02
185550	*p_Bacteroidetes;c_Bacteroidia;o_Bacteroidales;f_S24-7;g_;s_*	**1.65**	2.09E-02
421792	*p_Bacteroidetes;c_Bacteroidia;o_Bacteroidales;f_S24-7;g_;s_*	**2.10**	2.11E-02
188427	*p_Bacteroidetes;c_Bacteroidia;o_Bacteroidales;f_S24-7;g_;s_*	**1.64**	2.24E-02
211494	*p_Bacteroidetes;c_Bacteroidia;o_Bacteroidales;f_S24-7;g_;s_*	**1.69**	2.34E-02
216495	*p_Bacteroidetes;c_Bacteroidia;o_Bacteroidales;f_S24-7;g_;s_*	**1.71**	3.79E-02
215214	*p_Bacteroidetes;c_Bacteroidia;o_Bacteroidales;f_S24-7;g_;s_*	**2.05**	3.93E-02
198966	*p_Bacteroidetes;c_Bacteroidia;o_Bacteroidales;f_S24-7;g_;s_*	**1.95**	4.26E-02
341913	*p_Bacteroidetes;c_Bacteroidia;o_Bacteroidales;f_S24-7;g_;s_*	**1.66**	4.49E-02
175458	*p_Bacteroidetes;c_Bacteroidia;o_Bacteroidales;f_S24-7;g_;s_*	**1.64**	4.88E-02
343853	*p_Bacteroidetes;c_Bacteroidia;o_Bacteroidales;f_S24-7;g_;s_*	**1.68**	4.88E-02
389067	*p_Firmicutes;c_Bacilli;o_Lactobacillales;f_Lactobacillaceae;g_Lactobacillus;s_*	**5.14**	6.64E-11
198641	*p_Firmicutes;c_Bacilli;o_Lactobacillales;f_Lactobacillaceae;g_Lactobacillus;s_*	**2.01**	3.92E-02
807795	*p_Firmicutes;c_Bacilli;o_Lactobacillales;f_Lactobacillaceae;g_Lactobacillus;s_*	**1.99**	4.34E-02
851794	*p_Firmicutes;c_Bacilli;o_Lactobacillales;f_Lactobacillaceae;g_Lactobacillus;s_*	**1.82**	4.61E-02
**751601**	***p_Firmicutes;c_Bacilli;o_Lactobacillales;f_Lactobacillaceae;g_Lactobacillus_s_zeae/paracasei* (strain DG)**	**11.00**	**9.30E-84**
518033	*p_Firmicutes;c_Bacilli;o_Lactobacillales;f_Lactobacillaceae;g_Lactobacillus;s_zeae*	**8.54**	1.98E-35
770290	*p_Firmicutes;c_Bacilli;o_Lactobacillales;f_Lactobacillaceae;g_Lactobacillus;s_zeae*	**5.43**	4.86E-12
266330	*p_Firmicutes;c_Bacilli;o_Lactobacillales;f_Lactobacillaceae;g_Lactobacillus;s_zeae*	**4.06**	6.38E-07
4042947	*p_Firmicutes;c_Bacilli;o_Lactobacillales;f_Lactobacillaceae;g_Lactobacillus;s_zeae*	**2.51**	6.34E-03
73609	*p_Firmicutes;c_Bacilli;o_Lactobacillales;f_Lactobacillaceae;g_Lactobacillus;s_zeae*	**2.25**	1.99E-02
214919	*p_Firmicutes;c_Bacilli;o_Turicibacterales;f_Turicibacteraceae;g_Turicibacter;s_*	**-3.08**	1.26E-02
261097	*p_Firmicutes;c_Clostridia;o_Clostridiales;f_;g_;s_*	**-4.58**	3.66E-05
269176	*p_Firmicutes;c_Clostridia;o_Clostridiales;f_;g_;s_*	**2.82**	1.10E-02
195622	*p_Firmicutes;c_Clostridia;o_Clostridiales;f_;g_;s_*	**2.76**	1.36E-02
827376	*p_Firmicutes;c_Clostridia;o_Clostridiales;f_;g_;s_*	**-2.34**	1.46E-02
831409	*p_Firmicutes;c_Clostridia;o_Clostridiales;f_;g_;s_*	**2.65**	2.11E-02
210218	*p_Firmicutes;c_Clostridia;o_Clostridiales;f_;g_;s_*	**2.56**	2.35E-02
275366	*p_Firmicutes;c_Clostridia;o_Clostridiales;f_;g_;s_*	**2.83**	2.57E-02
234912	*p_Firmicutes;c_Clostridia;o_Clostridiales;f_Clostridiaceae;g_Clostridium;s_*	**2.16**	2.98E-02
265018	*p_Firmicutes;c_Clostridia;o_Clostridiales;f_Lachnospiraceae;g_;s_*	**5.37**	5.17E-08
4440360	*p_Firmicutes;c_Clostridia;o_Clostridiales;f_Lachnospiraceae;g_;s_*	**-3.15**	1.53E-03
351881	*p_Firmicutes;c_Clostridia;o_Clostridiales;f_Lachnospiraceae;g_;s_*	**2.03**	3.60E-02
2120784	*p_Firmicutes;c_Clostridia;o_Clostridiales;f_Lachnospiraceae;g_;s_*	**-2.06**	3.60E-02
163153	*p_Firmicutes;c_Clostridia;o_Clostridiales;f_Ruminococcaceae;g_Oscillospira;s_*	**3.00**	4.51E-03
167420	*p_Firmicutes;c_Erysipelotrichi;o_Erysipelotrichales;f_Erysipelotrichaceae;g_;s_*	**2.06**	3.79E-02
2645488	*p_Firmicutes;c_Erysipelotrichi;o_Erysipelotrichales;f_Erysipelotrichaceae;g_;s_*	**2.22**	4.34E-02
437137	*p_Proteobacteria;c_Betaproteobacteria;o_Burkholderiales;f_Alcaligenaceae;g_Sutterella;s_*	**2.35**	1.42E-02
363731	*p_Verrucomicrobia;c_Verrucomicrobiae;o_Verrucomicrobiales;f_Verrucomicrobiaceae;g_Akkermansia;s_muciniphila*	**-4.39**	7.57E-06

min **max** **-4.6** **+11.0**

Fig. S5. Results of the gene expression analyses performed by RT-qPCR. Gavage with PBS has been used as control condition. FOI, fold of induction. Statistics was according to Mann-Whitney U test; **, P<0.01; *, P<0.05.

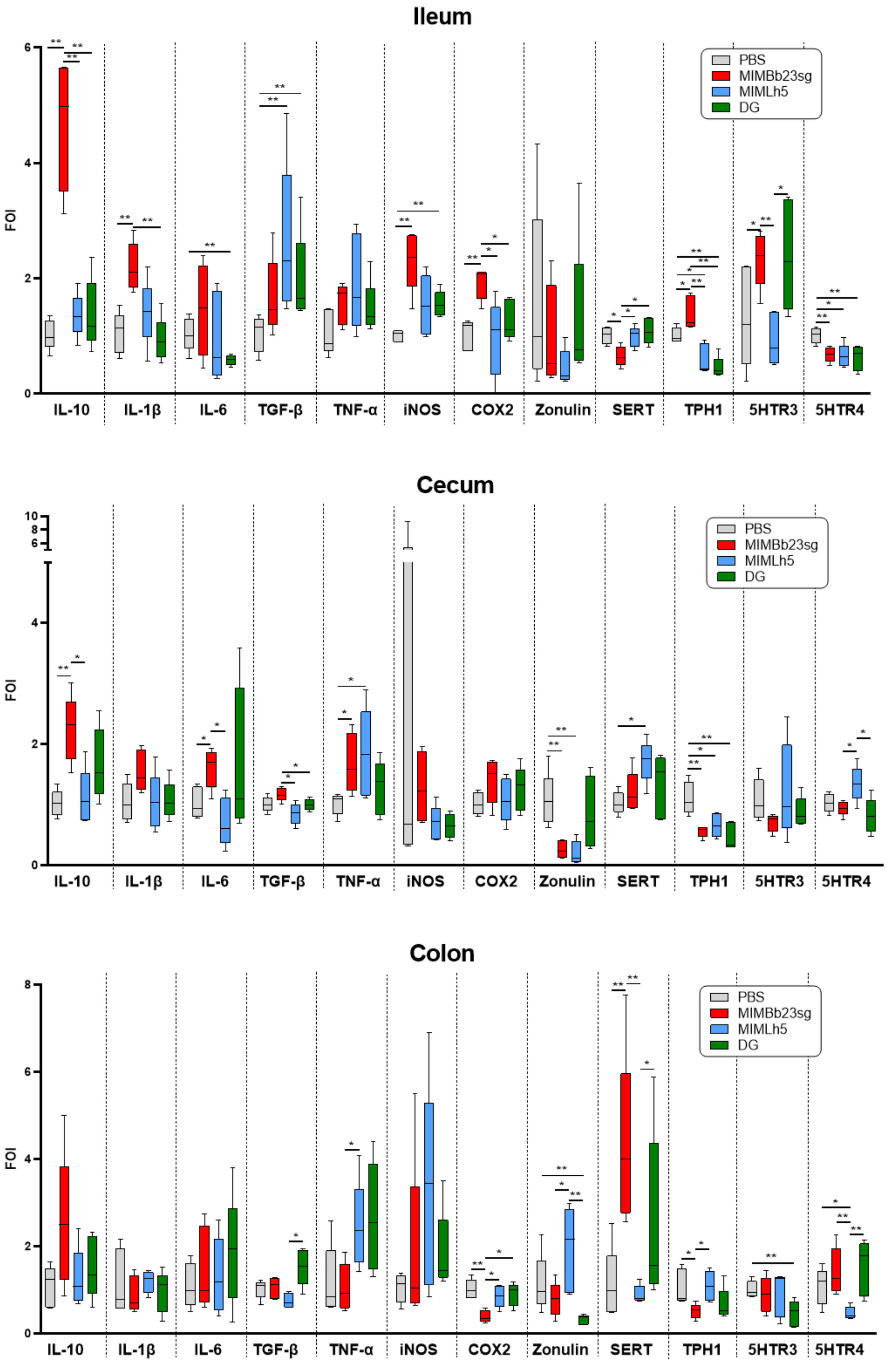

References

Chetty, N., Irving, H.R., and Coupar, I.M. (2006). Activation of 5-HT3 receptors in the rat and mouse intestinal tract: a comparative study. British journal of pharmacology *148*, 1012-1021. 10.1038/sj.bjp.0706802.

Haub, S., Ritze, Y., Bergheim, I., Pabst, O., Gershon, M.D., and Bischoff, S.C. (2010). Enhancement of intestinal inflammation in mice lacking interleukin 10 by deletion of the serotonin reuptake transporter. Neurogastroenterology and motility : the official journal of the European Gastrointestinal Motility Society *22*, 826-834, e229. 10.1111/j.1365-2982.2010.01479.x.

Montbarbon, M., Pichavant, M., Langlois, A., Erdual, E., Maggiotto, F., Neut, C., Mallevaey, T., Dharancy, S., Dubuquoy, L., Trottein, F., et al. (2013). Colonic inflammation in mice is improved by cigarette smoke through iNKT cells recruitment. PloS one *8*, e62208. 10.1371/journal.pone.0062208.

Muyzer, G., de Waal, E.C., and Uitterlinden, A.G. (1993). Profiling of complex microbial populations by denaturing gradient gel electrophoresis analysis of polymerase chain reaction-amplified genes coding for 16S rRNA. Applied and environmental microbiology *59*, 695-700.

Sato, T., Matsumoto, T., Kawano, H., Watanabe, T., Uematsu, Y., Sekine, K., Fukuda, T., Aihara, K., Krust, A., Yamada, T., et al. (2004). Brain masculinization requires androgen receptor function. Proceedings of the National Academy of Sciences of the United States of America *101*, 1673-1678. 10.1073/pnas.0305303101.

Taverniti, V., Stuknyte, M., Minuzzo, M., Arioli, S., De Noni, I., Scabiosi, C., Cordova, Z.M., Junttila, I., Hamalainen, S., Turpeinen, H., et al. (2013). S-layer protein mediates the stimulatory effect of Lactobacillus helveticus MIMLh5 on innate immunity. Applied and environmental microbiology *79*, 1221-1231. 10.1128/AEM.03056-12.
